# Effects of an over-the-counter lactic-acid containing intra-vaginal douching product on the vaginal microbiota

**DOI:** 10.1186/s12866-019-1545-0

**Published:** 2019-07-25

**Authors:** C. van der Veer, S. M. Bruisten, R. van Houdt, A. A. Matser, G. Tachedjian, J. H. H. M. van de Wijgert, H. J. C. de Vries, J. J. van der Helm

**Affiliations:** 1 Department of Infectious Diseases, Public Health Service, GGD, Amsterdam, the Netherlands; 2 Amsterdam Infection & Immunity Institute Academic Medical Centre, University of Amsterdam, Amsterdam, the Netherlands; 3 Department of Medical Microbiology and Infection prevention, VU University Medical Center, Amsterdam, the Netherlands; 4 Disease Elimination Program Life Sciences Discipline, Burnet Institute, Melbourne, Victoria Australia; 5 Department of Microbiology, Monash University, Clayton, Victoria Australia; 6 Department of Microbiology and Immunology at the Peter Doherty Institute for Infection and Immunity, The University of Melbourne, Melbourne, Victoria Australia; 7 Institute of Infection and Global Health, University of Liverpool, Liverpool, UK; 8 Julius Center for Health Science and Primary Care, University Medical Center Utrecht, Utrecht, the Netherlands; 9 Academic Medical Centers, Department of Dermatology, Amsterdam, the Netherlands

## Abstract

**Background:**

Over-the-counter intra-vaginal lactic-acid containing douches are marketed as vaginal hygiene products that support optimal vaginal pH balance. We report the effect of a commercially available douche (Etos®) on the vaginal microbiota (VM) in a prospective study.

**Results:**

Twenty-five healthy women were recruited through advertisements in 2015–2017 (ethical approval: METC-2014_413) and followed over three menstrual cycles. The participants had a median age of 24 years [IQR: 22–29], were mostly Dutch-Caucasian (88%), and 60% used combined oral contraceptives. All participants douched three times a week during the second cycle, starting on the first day of that cycle. Participants completed a questionnaire at baseline, kept a daily diary to report douching, menses, and sexual activity, self-collected vaginal swabs every other day during the first and third cycle and daily during the second cycle, and measured vaginal pH mid-cycle. A median of 44 vaginal swabs [inter-quartile range (IQR): 41–50] were assessed per participant by 16S rRNA gene (V3-V4 region) sequencing and a *Candida albicans* PCR was done at four time-points. At baseline, 21 participants (84%) had *Lactobacillus*-dominated VM (*Lactobacillus crispatus* (*n* = 14), *L. iners* (*n* = 6), or diverse *Lactobacillus* species (*n* = 1) and 4 participants (16%) had VM consisting of diverse anaerobes. In multinomial logistic regression models, a trend towards increased odds were observed for having diverse anaerobic VM in the second and third cycle, compared to the first cycle, after adjusting for menses [odds ratio (OR) = 1.4 (95% CI: 0.9–2.1) and OR = 1.7 (95% CI: 0.9–3.1), respectively] (*p* = 0.376). Douching did not affect vaginal pH. Menses increased the odds for having VM consisting of diverse anaerobes almost two-fold (OR = 1.7; 95% CI: 1.0–2.8), while douching during menses increased the odds 2.6 fold (OR = 2.6; 95% CI: 1.0–6.5), compared to not menstruating (*p* = 0.099). Participants were more likely to test positive for *C. albicans* after cycle 2, compared to cycle 1 [OR = 3.0 (95% CI: 1.2–7.2); *p* = 0.017].

**Conclusion:**

The Etos® douche did not significantly affect the vaginal pH or VM composition, although increased odds for having diverse anaerobic VM was observed, especially when douching during menses. Furthermore, douching may promote *C. albicans* infections.

**Electronic supplementary material:**

The online version of this article (10.1186/s12866-019-1545-0) contains supplementary material, which is available to authorized users.

## Introduction

Women worldwide engage in vaginal hygiene practices. The products that women use vary and range from plain water to water and soap, detergents, vinegar, or home-brewed herbal steam baths [1]. Some vaginal hygiene practices are embedded in cultural habits, yet the most common reasons for intra-vaginal cleansing are personal hygiene, aesthetics, cleansing after sex or menses and prevention of infections or pregnancy (reviewed in [1]). In westernized countries the use of over-the-counter vaginal douches – devices that spray liquid into the vagina – is common. Of female visitors to the sexually transmitted infections (STI) clinic in Amsterdam, the Netherlands, 31% reported the use of vaginal cleansing products including douches (unpublished data). Over-the-counter douches differ in composition; the most common active ingredients are antiseptic compounds and lactic acid. They also often contain surfactants as excipients.

Vaginal douching may affect the composition of the vaginal microbiota (VM). Healthy VM are characterized by a high abundance of *Lactobacilli*. *Lactobacillus* species produce antimicrobial peptides and acidify the vagina (pH < 4.5) through lactic acid production and thus create a hostile environment for most pathogens [2, 3]. A loss of *Lactobacilli* and an overgrowth of other anaerobes is indicative of bacterial vaginosis (BV) and is associated with increased risk of HIV acquisition and transmission, and preterm birth [4–8]. Several observational studies have reported an association between vaginal douching and prevalent BV [9–14], suggesting that douching may disrupt healthy VM compositions and predispose women to BV. Women with BV may also be more likely to douche in an effort to eliminate the (symptoms of) BV.

The effect that douching may have on the VM depends on the product that is used. In vitro and animal model studies have shown that products consisting mainly of water and vinegar have no effect on the growth of *Lactobacilli*, but vinegar inhibits the growth of a select few vaginal pathogens [15, 16], whereas spermicidal or antiseptic products with detergents inhibit the growth of *all* vaginal organisms [15–17] and also possibly cause irritation to mucosal surfaces by inducing pro-inflammatory responses [16, 17]. Studies in humans have shown that vaginal gels containing lactic acid, are well tolerated in the vagina as they do not induce irritation, though these products have not consistently been found to prevent or cure (recurrent) BV [18–20].

Despite increased public health concern for the effect of intra-vaginal douching on vaginal health, it remains an understudied topic bounded by inconclusive evidence. A few longitudinal studies have looked into the association between douching and BV, but have only studied natural douching behavior, as opposed to studying the effect of a specific type of product and/or frequency of douching [9, 10, 21, 22].

Over-the-counter intra-vaginal douches containing lactic acid are marketed as being safe to use and as effective in relieving vaginal symptoms, such as abnormal discharge or odor, by cleansing the vagina, and restoring the natural (pH) balance. We performed a pilot intervention study to evaluate the effect of an over-the-counter lactic-acid containing intra-vaginal douche (Etos®) on the composition of VM in a Dutch population of healthy women at reproductive-age.

## Results

### Study population

Twenty-nine participants were recruited, from September 2015 to January 2017. Two participants were excluded from the study after cycle 1 as they had forgotten to sample for more than 1 week. A further two participants were lost to follow-up, leaving a total of 25 participants who completed the study for analyses. Three participants interrupted sampling for one cycle (VH02, VH16 and VH25) during the study period due to vacationing overseas. Characteristics of the study population are shown in Table 1. The median age was 24 years (IQR: 22–29) and most participants were Dutch-Caucasian (*n* = 22; 88%); the others had either European/ African mixed, Eastern-European or Middle-Eastern ancestry. All participants were highly-educated and none had had children. Five participants (20%) were moderate smokers (less than five cigarettes per day) and the others did not smoke (*n* = 20; 80%). All except one participant reported regular menses, with a median self-reported cycle length of 28 days (IQR: 28–28). Just over half (*n* = 15; 60%) of the study population used hormonal contraception (in all cases combined oral contraceptive pills), and most participants reported a single sex partner (*n* = 20; 80%) in the last month. None of the participants had used an intra-vaginal douche in the last 6 months before study entry.

**Table 1:** Baseline characteristics of 25 study participants enrolled at the Public Health Service clinic in Amsterdam, the Netherlands, from September 2015 to January 2017

Demographics	*N* = 25 (%)
Age, median [IQR]	24 [22–29]
Ethnic background	
Dutch	22 (88)
Middle-Eastern	1 (4)
Eastern European	1 (4)
African (paternal heritage)	1 (4)
Has ever been pregnant	1 (4)
Has any children	0 (0)
Completed higher education	25 (100)
Smoking	5^a^ (20)
Medication use last 30 days	
Antibiotics use	0 (0)
Flucanozol	1 (4)
Corticosteroids	1 (4)
Valaciclovir	1 (4)
Combined oral contraceptives	15 (60)
Menstrual cycle	
Regular menses	24 (96)
Length of menstrual cycle, median [IQR] (*n* = 24)	28 [28–28]
Protection during menses	
Tampons	18 (72)
Sanitary pads	2 (8)
Tampons & sanitary pads	4 (16)
Menses cup	1 (4)
Sexual behavior	
Sex with men only	24 (96)
Sex with women only	1 (4)
Number of sex partners last 30 days	
0	5 (20)
1	20 (80)
(Regular) condom use	9 (36)
Self-reported urogenital symptoms	
Vaginal discharge	7 (28)
Pain during sex	1 (4)
Vaginal hygiene behavior last 6 months	
External vaginal cleansing product	
Only water	15 (60)
Water & lactic acid containing product	9 (36)
Water & soap	1 (4)
Tool used to apply cleansing product	
Only hands	23 (92)
Hands & washing cloth or tissue	2 (8)
Used an intra-vaginal cleansing product	0 (0)

^a^ < 5 cigarettes per day

### Intra-vaginal douching product

The lactic acid concentration of the neat Etos douching product was 0.45% and calculated to be 0.06% when diluted in water (1/7). The pH of the neat product was 3.42 and of the diluted product 3.50.

### Perception and reported experience of intra-vaginal douching

Nineteen participants completed the evaluation questionnaire. Six women reported feeling fresh and one woman reported reduced vaginal symptoms (vaginal itching, dryness, odor etc.) after douching, whereas five women reported no effects, five women reported dryness, and two women reported increased vaginal symptoms after douching. Furthermore, only three women reported douching as pleasant and the majority (*n* = 16/19) would not recommend douching to a friend or use the product themselves again in the future (Additional file 1: Figure S2).

### Clinical findings: changes in vaginal pH, Nugent score and BV by Amsel criteria

The vaginal pH was stably low (~pH 4) throughout the study period for most participants (*n* = 19; 76%), and stably high (~pH 5.5) for one participant. Five participants experienced pH shifts of more than one pH unit over the study period, though no significant association with douching was observed (Additional file 2: Tables S1 and S2). No increased odds for BV or intermediate microbiota as defined by Nugent score were observed during or after douching, compared with before douching (Additional file 2: Tables S1 and S3). None of the participants fulfilled the Amsel criteria for BV at the start or end of the study and only one participant fulfilled the Amsel criteria for BV at the start of cycle 2 (Additional file 2: Table S1).

### VM composition by 16 s rRNA gene targeted sequencing

**Fig. 1:**
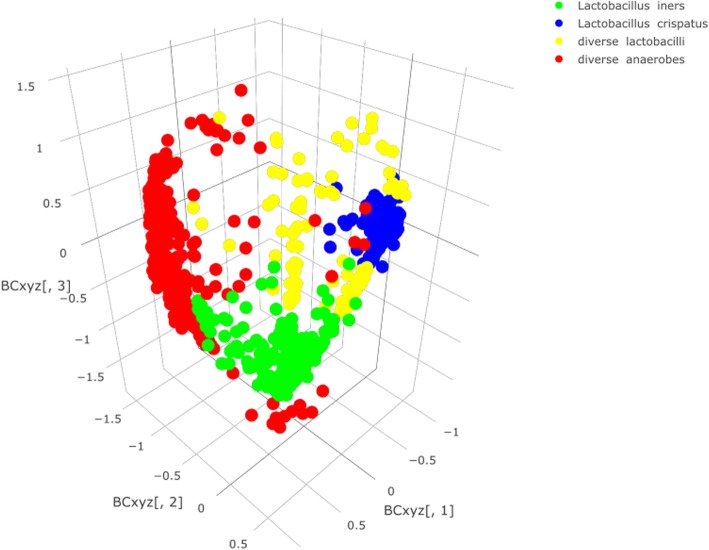
Non-metric multidimensional scaling (nMDS) of vaginal samples, colour coded by allocated VM group. nMDS values were calculated and visualized for three dimensions using the operational taxonomic unit (OTU)-based Bray-Curtis metric that assesses OTU presence/absence and OTU abundance (i.e. diversity). Dots closer together are more similar in microbial composition. Blue dots represent *L. crispatus*-dominated samples, green dots represent *L. iners-*dominated samples, red dots represent vaginal samples with diverse anaerobes and yellow dots represent samples with diverse lactobacilli

In total, a median of 64 (IQR: 62–70) vaginal swabs were collected per participant, with a median of 15 (IQR: 14–16) swabs in cycles 1 and 3 each and a median of 36 swabs (IQR: 31–40) in cycle 2. A median of 44 swabs (IQR: 41–50) were selected per participant for 16 s rRNA gene analyses. A total of 1100 samples were sequenced with a median of 20991 (IQR: 5901–43394) reads per sample; 39 samples (3.9%) were excluded as they produced < 100 reads. Overall, 562 OTUs were generated and allocated to 503 different taxonomies with a minimum likelihood > 0.8 (Additional file 1: Figure S3 for the pertaining heatmap). The samples belonged to the following VM groups: 1) *Lactobacillus crispatus-*dominated (*n* = 418 samples); 2) *Lactobacillus iners*-dominated (*n* = 290 samples); 3) VM containing several *Lactobacillus* species (named ‘Diverse Lactobacilli’, *n* = 114 samples); and 4) polybacterial VM - with or without *G. vaginalis* (named ‘Diverse anaerobes’, *n* = 239 samples) (more detailed descriptions in Additional file 2: Table S4). Visualization of the nMDS ordination values offered support for the grouping of samples into these four VM composition groups (Fig. 1).

### VM composition over time

**Fig. 2:**
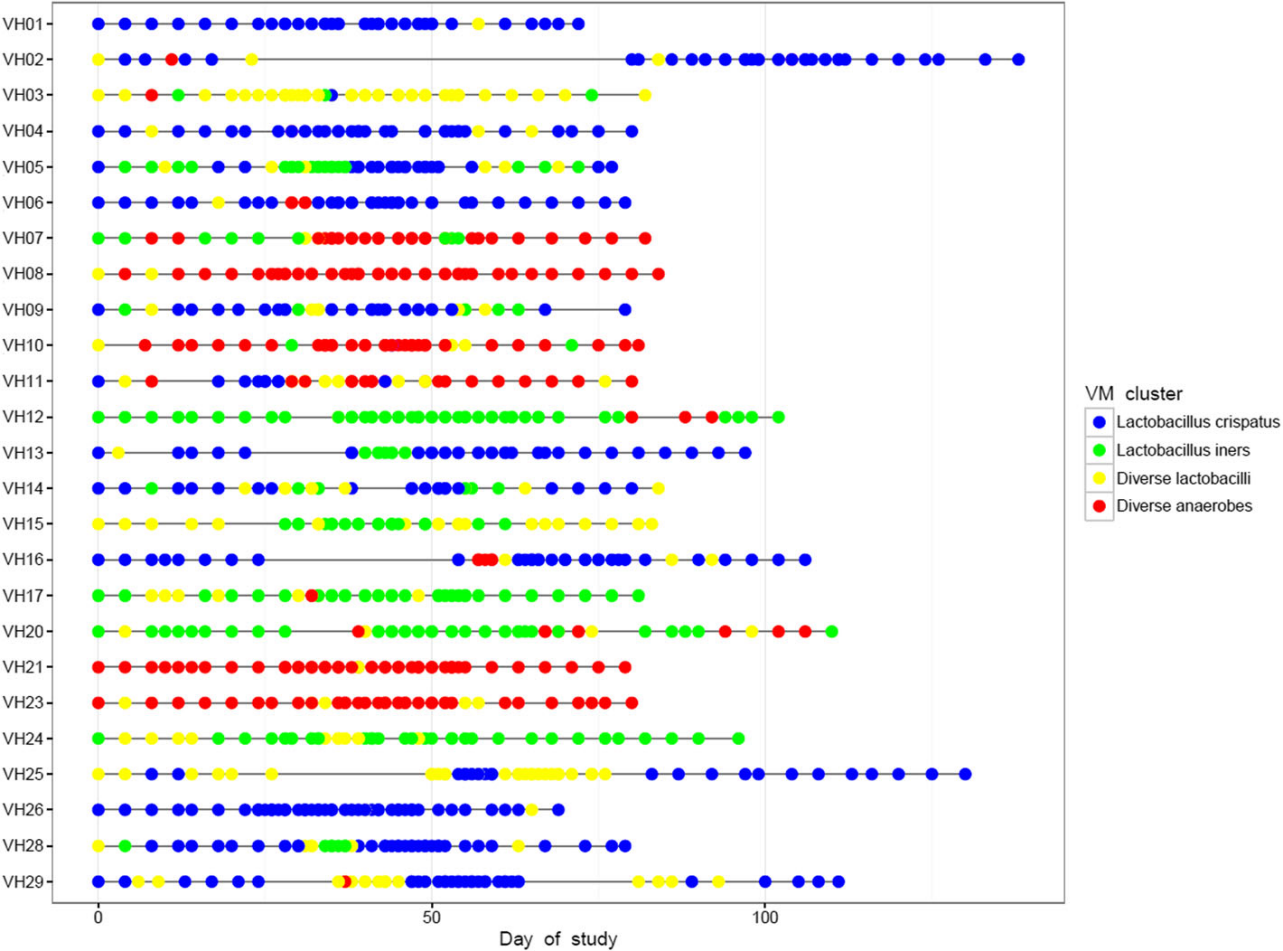
A Cleveland dot plot representing the allocated VM groups at each sampling point, per participant, over the entire study period. Blue dots represent *L. crispatus*-dominated samples, green dots represent *L. iners-*dominated samples, red dots represent vaginal samples with diverse anaerobes and yellow dots represent samples with diverse lactobacilli. Study duration depended on the length of the participant’s menstrual cycle during the study period. Three participants interrupted sampling for one cycle (VH02, VH16 and VH25), but recommenced the cycle thereafter

**Fig. 3:**
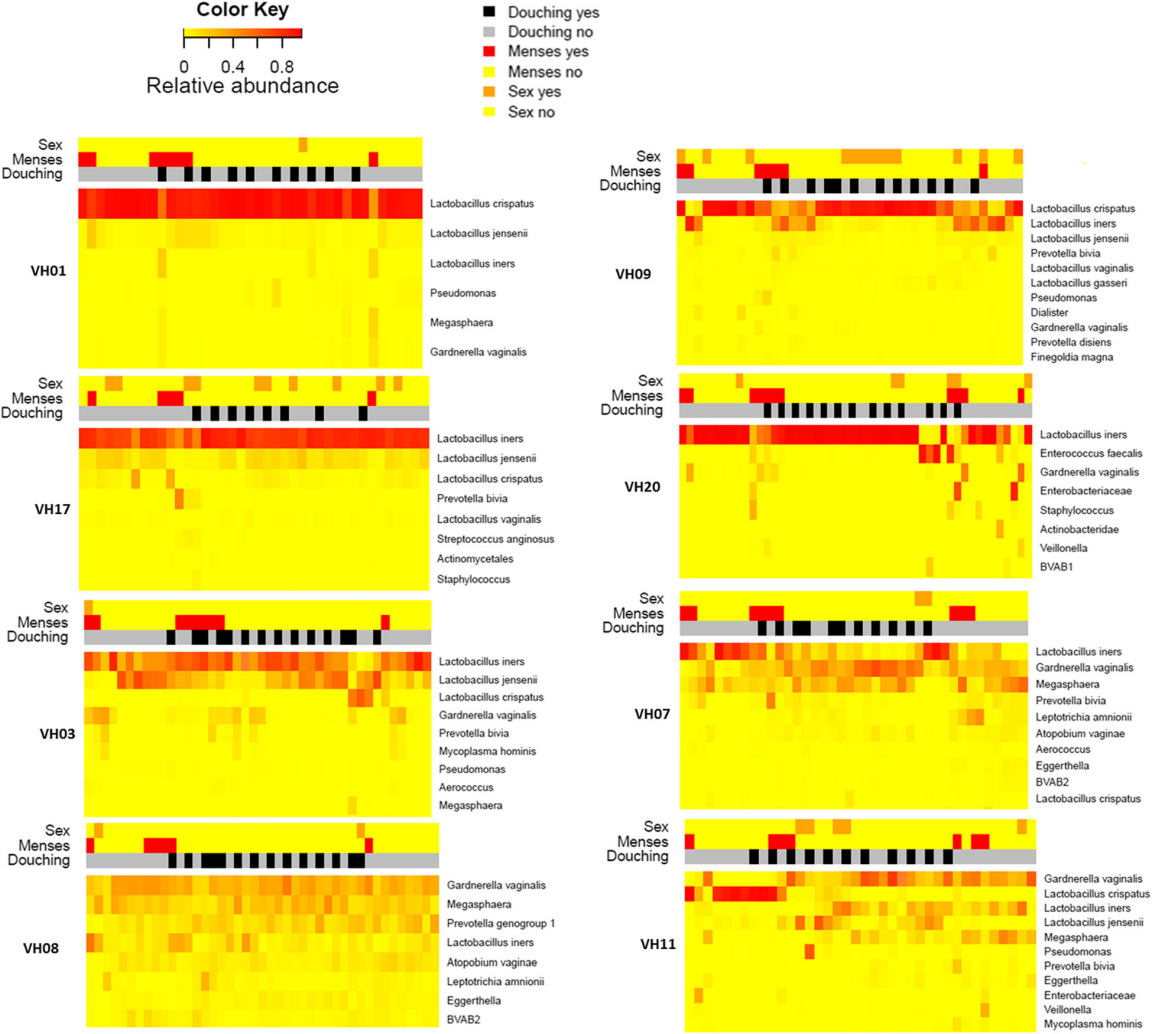
Chronological (from left to right) representation of microbial species (y-axis) of vaginal samples from 8 participants whose VM represent the different types of VM patterns that were observed in this study; VH01: stably *L. crispatus*-dominated VM; VH17: stably *L. iners-*dominated VM; VH03: stably Diverse Lactobaclli; VH09: stably Diverse anaerobes; VH09: temporal microbial shifts associated with menses; VH20, VH07 and VH11: long-lasting microbial shifts. Additional file 3 for heatmaps of all 25 participants. Microbial relative abundance is illustrated by the colour key. The sidebars above the heatmap depict self-reported vaginal intercourse (top bar), menstruation (middle bar) and intra-vaginal douching 1 h prior to sampling (bottom bar)

When assessing the VM compositions per participant over time as visualized in Cleveland dot plots (Fig. 2) and heatmaps (Fig. 3 and Additional file 3) we observed that eight (32%) participants had relatively stable VM compositions throughout the study period: *L. crispatus-*dominated (*n* = 3), *L. iners*-dominated (*n* = 2), and diverse anaerobes (*n* = 3). Fifteen (60%) participants experienced microbial shifts during or around the time of menses, with an increase in the relative abundance observed for *L. iners* (*n* = 7), *L. jensenii* (*n* = 3), other anaerobes (*n* = 3), or aerobes (*n* = 2). Two (8%) participants experienced major VM shifts without reverting back to their original states during the study period. One participant’s *L. iners-*dominated VM in cycle 1 shifted to a *G. vaginalis* and Megaspheara-containing VM in cycle 2 that remained so in cycle 3, although *L. iners* relative abundance increased during menses (participant VH07). Another participant had *L. crispatus-*dominated VM in cycle 1 that shifted to an *L. iners, G. vaginalis* and *Megaspheara* containing polybacterial VM during cycle 2 and this remained so in cycle 3 with no further fluctuations around the time of menses (participant VH11) (Fig. 3).

**Fig. 4:**
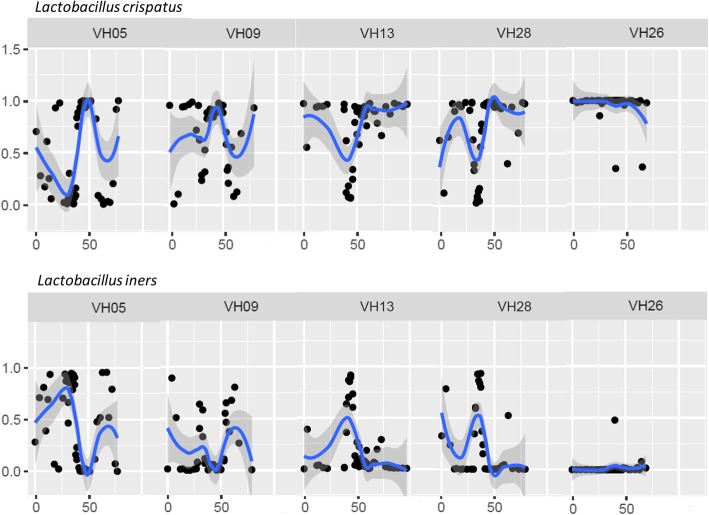
Visualization of the relative abundances (y-axis) of *Lactobacillus crispatus* and *Lactobacillus iners* with polynomial curves using locally weighted smoothed regression with 95% confidence intervals over time (x-axis; day of study) for five participants whose relative abundance of *L. crispatus* and *L. iners* fluctuated swiftly by mutual exclusion. Additional file 4 for several other bacterial species over time of all 25 participants

The relative abundance of single bacterial species was visualized over time (Fig. 4 and Additional file 4). These plots showed that *L. crispatus* and/or *L. iners* were present in all participants, often in high relative abundance (up to 99%). Noteworthy, their relative abundance could fluctuate swiftly and often by mutual exclusion: when the relative abundance of *L. crispatus* increased, the relative abundance of *L. iners* decreased and vice versa (Fig. 4). *L. jensennii* was less common, occurring in 11 participants, and present in lower relative abundance (< 70%). *L. gasseri* was rare, showing low relative abundance (< 40%) in just four participants.

### Association between douching and VM composition

Univariable and multivariable associations between intra-vaginal douching and vaginal microbiota are shown in Table 2. Douching was not significantly associated with VM composition, although increased odds for having diverse anaerobes, compared to *L. crispatus*-dominated VM, were observed during the second (douching; OR = 1.4; 95% CI: 0.9–2.2) and third (douching cessation; OR = 1.7, 95% CI: 0.9–3.1) cycle, compared to the first (before douching) cycle, after having adjusted for menses on the day of sampling (*p* = 0.376). Menses was significantly associated with VM composition (*p* = 0.025) and increased the odds of having *L. iners-*dominated VM (OR = 1.9; 95% CI: 1.1–3.2), diverse lactobacilli (OR = 2.2; 95% CI: 1.0–4.5), and diverse anaerobes (OR = 1.7; 95% CI: 1.0–2.9), each compared to having *L. crispatus-*dominated VM. Douching during menses further increased the odds of having diverse anaerobes (OR = 2.6; 95% CI: 1.0–6.5) compared to *L. crispatus-*dominated VM, at borderline statistical significance (*p* = 0.099). Oral contraceptive use at baseline and vaginal intercourse on the day of sampling were not associated with VM composition. We did not observe any short-term effect of douching on the VM composition by LefSe analysis, i.e. no differentially abundant features were detected between samples taken before and 1 h after douching. Douching did not affect within sample diversity either, whereas menses slightly increased the within sample diversity (χ^2^ (1) =10.48, *p* = 0.001) by increasing the Shannon’s diversity index score by 0.085 ± 0.023 (Additional file 2: Table S5 and Additional file 1: Figure S4).

**Table 2:** Univariable and multivariable multinomial logistic regression analysis of the association between intra-vaginal douching and vaginal microbiota, referenced to *Lactobacillus crispatus-*dominated microbiota, of 25 study participants enrolled at the Public Health Service clinic in Amsterdam, the Netherlands, from September 2015 to January 2017

		Univariable model				Multivariable model			
	No. of samples *N* = 1061	*Lactobacillus iners-*dominated microbiota Univariable OR^a^ (95% CI)	Diverse Lactobacilli Univariable OR^a^ (95% CI)	Diverse anaerobes Univariable OR^a^ (95% CI)	*P*-value	*Lactobacillus iners-*dominated microbiota Multivariable OR^a^ (95% CI)	Diverse Lactobacilli Multivariable OR^a^ (95% CI)	Diverse anaerobes Multivariable OR^a^ (95% CI)	*P*-value
Study Cycle					0.321				0.376
First cycle (no douche)	207	Ref	Ref	Ref		Ref	Ref	Ref	
Second cycle (douching)	650	1.0 (0.7–1.5)	0.8 (0.5–1.3)	1.4 (0.9–2.1)		1.0 (0.7–1.5)	0.8 (0.5–1.4)	1.4 (0.9–2.1)	
Third cycle (wash-out)	204	1.0 (0.6–1.7)	0.6 (0.2–1.7)	1.5 (0.8–2.8)		1.0 (0.6–1.8)	0.6 (0.2–1.8)	1.7 (0.9–3.1)	
Vaginal intercourse on day of sampling	155	1.1 (0.6–2.0)	0.9 (0.6–1.3)	1.0 (0.4–2.4)	0.814				
Menses on day of sampling	208	1.8 (1.1–3.2)	2.2 (1.1–4.4)	1.7 (1.0–2.8)	**0.025**	1.9 (1.1–3.2)	2.2 (1.0–4.5)	1.7 (1.0–2.9)	**0.025**
Combined oral contraceptive use	600	0.9 (0.2–5.2)	0.9 (0.2–3.2)	0.9 (0.1–5.5)	0.997				
Interaction menses and douching					0.099				
No menses	601	Ref	Ref	Ref					
Menses, no douching	376	2.0 (1.2–3.2)	2.0 (0.9–4.2)	1.5 (0.9–2.4)					
Menses and douching	84	2.1 (1.0–4.5)	3.0 (1.0–9.4)	2.6 (1.0–6.5)					

^a^Adjusted for multiple (within subject) measurements using generalized estimating equations and referenced to *Lactobacillus crispatus-*dominated VM

Bold values denote statistical significance at the *p* < 0.05 level

### *Candida albicans* PCR outcome

Increased odds for a positive *C. albicans* PCR outcome were observed after douching, compared with before douching (OR = 3.0 (95% CI: 1.2–7.2); *p* = 0.017): four participants (16%) tested PCR positive for *C. albicans* at the start of cycles 1 and 2, whereas nine participants (36%) tested positive at the end of cycle 2. Three participants (12%) tested positive at the end of cycle 3 (Table 3). Six participants reported yeast-like symptoms in their diaries, of whom five reports coincided with positive test outcomes of our in-house PCR for *C. albicans* (Additional file 2: Table S6).

**Table 3:** Univariable logistic regression analysis of the association between intra-vaginal douching and positive PCR outcome for *Candida albicans*, of 25 study participants enrolled at the Public Health Service clinic in Amsterdam, the Netherlands, from September 2015 to January 2017

	No. of samples	Positive PCR outcome for *Candida albicans* Univariable OR^a^ (95% CI)	*P*-value
Study time-point			0.072
Start of study	4/25	Ref	
Start of Cycle 2	4/25	0.7 (0.2–2.2)	0.563
End of Cycle 2	9/25	3.0 (1.2–7.2)	**0.017**
End of Study	3/25	1.3 (0.5–3.3)	0.562

^a^Adjusted for multiple (within subject) measurements using generalized estimating equations

Bold values denote statistical significance at the *p* < 0.05 level

## Discussion

Over-the-counter lactic-acid containing douches are marketed as products that promote vaginal health by cleansing the vagina while maintaining or lowering the vaginal pH. Our pilot intervention study showed that regular use of a commercial intra-vaginal lactic-acid containing douche (Etos®) did not significantly improve the VM composition (defined by domination of *Lactobacilli*) or influence the vaginal pH. We observed that douching increased the odds of having VM containing diverse anaerobes, especially when douching during menses, although this did not reach statistical significance. Moreover, we found that douching increased the odds of testing positive for *C. albicans,* which is the main causative agent of vulvovaginal candidiasis [23].

Our intervention study provides advancement over previous studies as our comprehensive approach included: 1) multiple methods to assess the VM (Amsel criteria, Nugent scoring and 16 s rRNA gene sequencing); 2) a standardized intervention (all participants used the same douching product and used it with similar frequency); 3) sampling before, during and after douching product use; 4) more frequent sampling than previously reported; and 5) data on potential confounders such as sexual intercourse and menses. Douching *cessation* was previously studied prospectively, and these studies did not detect an overall effect on the VM composition [9, 22, 24], except for women who primarily douched to cleanse after menses [9]. However, these studies relied on self-reported douching events and participants within the same study populations did not all douche with the same product and/or frequency. Moreover, these studies did not study the effect of douching *initiation*, which is important for detecting any benefits douching may have on (dysbiotic) VM. Vaginal hygiene behaviors encompass a wide variety of practices. We focused our study on an intra-vaginal over-the-counter lactic-acid containing product because the use of lactic-acid products was most commonly reported among female visitors of the STI clinic in Amsterdam (personal communication), and we hypothesized that applying the product intra-vaginally would convey the strongest effect.

The women in our study acted as their own controls. Samples collected during cycle 1 were considered baseline samples to which the samples collected during the intervention and wash-out periods were compared. We used generalized estimating equations (GEE) to account for multiple measurements from the same participant. The VM composition outcomes were categorized into four different clinically and biologically relevant VM composition groups. Samples from the same individual could belong to different VM groups within one cycle, but the GEE analysis computed a generalized estimate for each individual per cycle and used this parameter as input for the multinomial logistic regression. As this is a simplification of the data, we built an additional linear mixed effects model using the Shannon diversity index as outcome (on a continuous scale). We obtained similar results for these two analytic methods, and therefore believe that our analyses accurately captured the effect of douching.

Very few prospective intervention studies have studied the effect of a specific intra-vaginal product on the VM. Two trials on the safety of intra-vaginal application of lime juice – a product commonly used by African sex workers – showed that lime juice did not affect vaginal pH or the VM as assessed by Nugent score [25, 26], compared to the baseline measurement. The perceived health benefit of lime juice, and also of lactic-acid containing products, is that the acidity cleanses and helps to protect the vagina against infections. The average physiological lactic acid concentration in neat cervicovaginal fluid from women with *Lactobacillus*-dominated VM is ~ 1% [27, 28]. Biological activities of lactic acid that have been measured (in vitro) i.e. antimicrobial activity (against BV bacteria), and anti-HIV and anti-inflammatory effects on cervicovaginal epithelial cells, are within the physiological concentrations reported in women with *Lactobacillus*-dominated microbiota [27, 28]. At ≥pH 4.2, HIV virucidal activity of lactic acid is dramatically diminished below the threshold concentration of 0.3% lactic acid [28] as well as anti-inflammatory effects on cervicovaginal epithelial cells/tissue [29]. In studies performed by O’Hanlon et al., the in vitro anti-bacterial (BV) effects were minimal at 0.1% lactic acid (pH 4.5) [30]. The lactic acid concentration of the neat Etos® douching product was 0.45% and when diluted in water (1/7) would be 0.06%. This is well below physiological concentrations in women with *Lactobacillus*-dominated microbiota, and below the concentrations for which the biological effects of lactic acid have been observed. The pH of the lactic acid douche that we used in our study was similarly low (pH 3.5) for the neat and diluted douching product, and given the low lactic acid concentrations, i.e. 0.1% lactic acid is reported to acidify culture medium to pH 4.9 ± 0.2 [28], it is unclear what is driving the acidity. We compared the ingredients of the Etos® product to other brands of vaginal hygiene products and observed very similar lists of ingredients (data not shown). Common to cosmetic and hygiene products, the Etos® douching product listed the preservatives caprylyl glycol and butylene glycol both of which have antimicrobial activities. No study has looked at the effect of such compounds on the VM, but their presence could in part explain the trend towards having diverse anaerobic VM that we observed, in addition to the physical action of douching.

That douching may negatively impact the VM composition has long been proposed [31]. In support of this, large-scale prospective observational studies have shown that women who reported douching (defined as “fluid to flush out vagina” [10], with mostly commercial products [21], or with mostly washing cloths [32]) were more likely to develop BV, as assessed by Nugent scoring, than women who did not report douching, independent of other feminine hygiene behaviors [12]. Particularly in Sub-Saharan Africa where intra-vaginal practices are very common [24, 31], intra-vaginal cleansing with soap seems to increase the risk for BV (pooled adjusted OR: 1.24; 95% CI: 1.04–1.47), which in turn may increase susceptibility for HIV acquisition (as summarized by Low et al. [33]). However, causal links could not be made as the reason for douching may still have been to alleviate BV symptoms. Douching behavior was nonetheless discouraged. Intervention studies that have assessed the effect of douching cessation have not observed an overall significant effect of douching cessation on the VM [9, 22, 24]. It should be noted though that two of these studies [22, 24] were primarily designed to evaluate the effectiveness of implementing interventions aimed at reducing douching behavior, whereas our study and that of Brotman et al. [9] had as the main objective to study the effect of douching (cessation) on the VM. Interestingly, in a sub-analysis, Brotman et al. [9] observed that douching cessation significantly reduced BV as assessed by Nugent scoring in women who primarily douched to cleanse after menses (adjusted OR:0.23; 95% CI:0.12–0.44). This supports our finding that douching during menses increased the odds, although not significantly, for having VM containing diverse anaerobes more so than menses alone (OR: 2.6; 95% CI: 1.0–6.5).

Previously it has been suggested that the VM may restore itself as quickly as 30–120 min after administration of an antiseptic vaginal hygiene product [34] and we therefore instructed participants to self-collect vaginal samples just before and 1 h after douching in order to capture any short-term effects of douching. We did not observe significant differences in the relative abundance of any bacterial species in samples taken before and 1 h after douching. Of note, we did not assess the *absolute* abundance, while it has been shown that douching with povidone-iodine solutions decreased the numbers, but not the types, of the cultivable bacterial species in ten healthy volunteers [35]. A reduction of vaginal bacteria, in particular *Lactobacillus* spp*.*, may pave the way for an increase of other organisms.

Several participants reported symptoms suggestive of a yeast infection in their diaries and reporting these symptoms coincided with positive test outcomes of our in-house PCR for *C. albicans*. Moreover, we observed significant increased odds for testing positive for *C. albicans* at the end of the second cycle, compared with the first cycle. Others have also observed a similar association [24, 26, 31, 36], where for example, douching cessation significantly lowered candidiasis prevalence (22 to 7%; *p* = 0.011) as assessed by wet mount microscopy [24].

Menses was significantly associated with changes in the VM, where most commonly increases in the relative abundance of *L. iners* were observed, seconded by increases in the relative abundance of *G. vaginalis*. These shifts were often transient and the VM usually reverted back to its original state within 1 week after menses. That menses alters the VM is well established [37–43] and it is thought that the drop in estrogen levels and the presence of menstrual blood may favor BV-associated bacteria such as *G. vaginalis*, but also *L. iners.* We did not observe an association between VM and having had sexual intercourse on the day of sampling. This might be because most participants reported a steady partner, while new sexual partnerships are often associated with inducing increased VM diversity [44, 45]. We did not observe an association between VM composition and hormonal contraceptive use either. The use of hormonal contraceptives has been associated with having *Lactobacillus-*, in particular *L. crispatus*-, dominated VM [46]. Our study population was possibly too small to observe this association, or perhaps this association is less pronounced in Caucasian populations, where *L. crispatus-*dominated VM are quite common [44, 47, 48].

The number of women included in our study was relatively small. However, by having the participants act as their own controls and by standardizing the intervention, the power to study the effect of this particular type of product was greatly increased. From a survey that was conducted at the Amsterdam STI clinic in 2008/2009, 31% of female clients reported use of lactic acid containing products (DWAR, unpublished results), so use of such products is common and thus applicable to a wide audience. The effect of douching may however differ by study population. In our study, most of the participants were Caucasian, nulli-gravida, reported low sexual risk behavior and were highly educated. Also, the participants in our study might not represent women who regularly douche as none of the participants had reported douching prior to study participation and most had reported that they would not use the douche again or recommend it to a friend because they considered it not healthy and unnecessary as the vagina is capable of cleansing itself.

## Conclusion

In this pilot intervention study we showed that the Etos® intra-vaginal lactic-acid containing douche did not significantly affect the VM composition, although shifts from an “optimal” low diversity *Lactobacillus-*dominated VM to “non-optimal” VM containing diverse anaerobes were observed, especially when douching during menses. Although there seems to be little to no effect on the VM, intra-vaginal douching likely promoted *C. albicans* infections and this, together with previous findings about douching during menses, ought to dissuade women from douching.

## Methods

### Study population

This study was performed at the Public health service (PHS) clinic in Amsterdam, the Netherlands. Ethical approval was obtained for this study from the Medical Ethics Council of the Amsterdam Medical Centre, reference number: METC 2014_413. We recruited women through word of mouth and through advertisements that were placed on the website of the PHS of Amsterdam and in the newsletters of two local universities. An incentive of 100 euros was rewarded at study completion. Women were eligible for the study if they: 1) were 18–36 years old; 2) had a regular menstrual cycle; and 3) tested negative for sexually transmitted infections (STI) (nucleic acid amplification tests for *Chlamydia trachomatis, Neisseria gonorrhoeae,* and *Trichomonas vaginalis* (APTIMA, Hologic, Marlborough, USA)), vulvovaginal candidiasis (based on microscopy), and BV by Amsel criteria at baseline. Women were excluded if they: 1) were pregnant, or planning to become pregnant within the next 3 months; 2) had an intra-uterine device (because some women with copper intra-uterine devices experience heavy menstrual bleeding and some women with Mirena intra-uterine devices experience amenorrhea and/or spotting; hormonal contraceptive pill and NuvaRing were allowed); 3) worked as a sex-worker; 4) had used antibiotics in the last 30 days; or 5) were allergic to any of the ingredients of the study product.

### Intra-vaginal douching product

The ingredients of the Etos® over-the-counter intra-vaginal douche were listed on the package label in the following order: aqua, butylene glycol, lactic acid, caprylyl glycol, sodium pyroglutamic acid, *Zea mays* kernel extract, hydrolyzed milk protein, niacinamide, and adenosine triphosphate. Concentrations per ingredient were not listed. The manufacturer’s instructions for use were as follows: add Etos® liquid and lukewarm water to the Etos® flask in a 1:7 ratio, as indicated by markings on the flask, twist on the nozzle, shake, and squirt slowly into the vagina once; replace the flask after ten uses. We measured the lactic acid concentration (both isomers) of the product (both neat and 1:7 dilution) using a commercial enzyme-based assay for quantitation of lactic acid (Boehringer/Mannheim Cat. No. 11 112 821 035, R-Biopharm, Darmstadt, Germany), following the manufacturer’s instructions. We measured the pH using an electrode.

### Clinical study design

The participants were followed over three menstrual cycles and were instructed to use the douching product three times weekly for the duration of cycle 2, starting on the first day of menses. We chose this frequency because the product insert advises a maximum of three times weekly douching. For each participant, the VM composition was assessed before initiation of product use (cycle 1), during product use (cycle 2), and after cessation of product use (cycle 3) (Additional file 1: Figure S1).

The participants visited the PHS clinic just before the start of each menstrual cycle where they were seen by a nurse who measured the vaginal pH, collected vaginal smears for Nugent scoring [49], and performed a ‘whiff’ test (fishy odor after addition of potassium hydroxide to vaginal smear). The participants were given instructions on how to take a vaginal swab and were instructed to swab every other day during the first and third cycle and daily during the second cycle. On days that the douche was used the participants were instructed to take a swab before douching and 1 h after douching. Self-collected vaginal swabs (Copan dry swabs (COPAN Diagnostics Inc., USA, Murrietta)) were stored in the participants’ home freezers (approximately − 18 °C) and these swabs were handed in during each clinic visit using a cooler box with ice-packs provided by the clinic. The participants were also instructed to take a pH measurement mid cycle using vaginal acidity test gloves (measures pH 4.0–7.0 at 0.3 increments; CarePlan VpH, UK, Bedford). Baseline characteristics were collected through self-administered questionnaires at the baseline visit. All participants kept a daily diary throughout the study period in which they recorded whether they douched, were menstruating, or were sexually active that day and if so, whether they used a condom and/or lubricants. Vaginal swabs were numbered sequentially and the corresponding number was recorded in the daily diary. At the last study visit, the participants were asked to complete an evaluation questionnaire on their perception and experience of using the intra-vaginal douche.

### Vaginal microbiota (VM) composition analysis

Vaginal swabs were stored at − 20 °C at the Public Health Laboratory of the PHS of Amsterdam until further processing. The following selection of swabs were assessed for VM composition: every first and last swab per cycle and every second swab in between, and all swabs collected before and after douching during cycle 2. Additional swabs were included if events of interest were reported in the daily diary, such as vaginal symptoms. For DNA extraction, the swabs were thawed, eluted in 800 μL phosphate buffered saline, and shaken for 30 min at room temperature. Two-hundred microliters of the eluted sample was used for DNA extraction by isopropanol precipitation, and the pellet was dissolved in 50 μl of 10 mM Tris/HCL [50]. Negative extraction controls were included in each batch, which were also sequenced.

The VM compositions were analyzed by targeted sequencing of the 16 s rRNA gene (V3-V4 region), as described previously [8, 51]. In short, pooled and normalized amplified DNA (using universal primers 319F and 806R) was sequenced on the illumina MiSeq platform (illumina, USA, San Diego) using the V3 reagent kit. High quality sequences (> 99% base call accuracy; Trimmomatic [52]) were retained and aligned using PandaSeq [53]. Operational taxonomic units (OTUs) were picked using the Usearch tool in QIIME (version 1.9) [54] and aligned to a vaginal reference package developed by Srinivisan et al. [55] using PPLACER [56]. Samples with less than 100 reads were excluded from further analyses. The Shannon diversity index was calculated per sample using the alpha diversity tool in QIIME [54]. Based on the dissimilarity matrix of relative abundances per OTU per sample (ggplot2 package [57] in R (version 3.2.1) together with microbiological characteristics, clinical meaningful VM groups were formed. We subsequently inspected their ordination using non-metric multidimensional scaling (nMDS) based on the Bray-Curtis dissimilarity metric that considers both taxa presence/absence and relative abundance of species (i.e. diversity) using the vegan and plotly packages in R (version 3.2.1).

### *Candida albicans* SYBR green PCR

An in-house validated SYBR green PCR based on the assay originally described by Zhang et al. [58] was used for the molecular detection of *Candida albicans*. In short, DNA was amplified using the 5.8S-1F and 28S-1R primers (RotoGene, Qiagen, Hilden, Germany), and PCR amplicons that melted in the range of 82.6 °C - 83.6 °C were considered positive for *C. albicans.* Samples from the following four study time points were tested for *C. albicans:* start of cycle 1, start of cycle 2, end of cycle 2 and end of cycle 3.

### Statistical analyses

Univariable logistic regression using generalized estimating equations (GEE) was used to determine the association between douching and vaginal pH (dichotomized to pH < 4.5 and pH > 4.5). Univariable multinomial logistic regression models were constructed to assess the association between douching and VM composition. In these models, the VM composition during cycle 1 (before douching) acted as reference to which the VM compositions during cycle 2 (douching) and cycle 3 (wash-out period) were compared. The ‘healthiest’ VM composition, *L. crispatus-*dominated VM, was set as the reference category to which the three other VM composition categories (*L. iners* dominated, diverse lactobacilli, and diverse anaerobes) were compared. The models accounted for multiple measurements within the same individual by using GEE. We assessed the association between VM composition and the variables: study cycle, combined oral contraceptive use at baseline, vaginal intercourse on the day of sampling, having menses on the day of sampling, and, to capture the effect of douching while menstruating, an interaction term for menses and douching on the day of sampling. Variables significantly associated with VM composition (*p* < 0.05) and that did not display collinearity were included in the multivariable model. All logistic regression analyses were performed using SPSS Statistics software version 21 (IBM, New York, USA).

To assess whether any short-term changes in VM composition occurred after douching, a linear discriminant effect size analysis (LefSe) [59] was performed on samples taken before and 1 h after douching. This analysis separated independent features (i.e. bacterial abundances) that were significantly discriminative between classes (i.e. before or after douching), by subclass (i.e. subject id). The threshold for the logarithmic effect size score for discriminative features was set to 2.0, with alpha set to 0.05.

A linear mixed effects model (LMER) in R using *lme4* [60] was constructed to study whether within-sample bacterial species diversity, i.e. alpha diversity, as defined by the Shannon diversity index, is affected by douching. Douching, menses, and sex on the day of sampling, and combined oral contraceptive use at baseline, were modeled as fixed effects and subject id as a random effect. The model also included by-subject random slopes for each of these variables. Visualization of residual plots showed no obvious deviations from normality. *P*-values were obtained by likelihood ratio tests of the full model with douching against a null-model without douching. A similar model was built to assess the effect of menses on VM species diversity. Median Shannon’s diversity index scores per participant per cycle were visualized using box plots.

The relative abundances of single bacterial species was visualized over time per participant with polynomial curves using locally weighted smoothed regression (loess) with 95% confidence intervals using the ggplot2 package in R [57]. Plots were made for the following species: *Atopobium vaginae,* BVAB 1, BVAB 2, *Gardnerella vaginalis, L. crispatus, L. gasseri, L. iners, L. jensennii, Leptotrichia amnionii,* Megaspheara, *Prevotella bivia,* Prevotella genogroup 1, and Prevotella genogroup 2.

## Additional files

Additional file 1: Graphic depictions of study design, questionnaire responses and clustering of vaginal microbiota compositions. (PDF 318 kb)

Additional file 2: Clinical and microbioligical outcomes. (XLSX 25 kb)

Additional file 3: Heatmaps per participant. (PDF 976 kb)

Additional file 4: Relative abundances per bacterial species. (PDF 1692 kb)

## Data Availability

The datasets used and/or analysed during the current study are available from the corresponding author on reasonable request.

## Abbreviations

BV: Bacterial vaginosis
GEE: Generalized estimating equations
LMER: Linear mixed effect regression
OTU: Operational taxonomic unit
STI: Sexually transmitted infections
VM: Vaginal microbiota
