# Supplementary material for: Effects of an over-the-counter lactic-acid containing intra-vaginal douching product on the vaginal microbiota
Source: BMC Microbiol. 2019 Jul 25;19:168. doi: 10.1186/s12866-019-1545-0 (PMC6659218; doi:10.1186/s12866-019-1545-0)

Figure S1. Study set-up. Healthy, non-pregnant women at reproductive age (18-36 years old) with a regular menstrual cycle were followed over three menstrual cycles and were instructed to use an over-the-counter intra-vaginal lactic-acid-containing douche three times a week for the duration of cycle 2, starting on the first day of menses. The participants visited the PHS clinic just before the start of each menstrual cycle where they were seen by a nurse who performed a vaginal pH measurement, collected vaginal smears and performed a KOH whiff test. At home the participants collected vaginal self-swabs every other day during the first and third cycle and a daily swab during the second cycle and on days that the douche was used, the participants collected a swab before douching and one hour after douching. Furthermore, the participants measured the vaginal pH at mid cycle and recorded sexual activity, douching and menses in a daily diary.

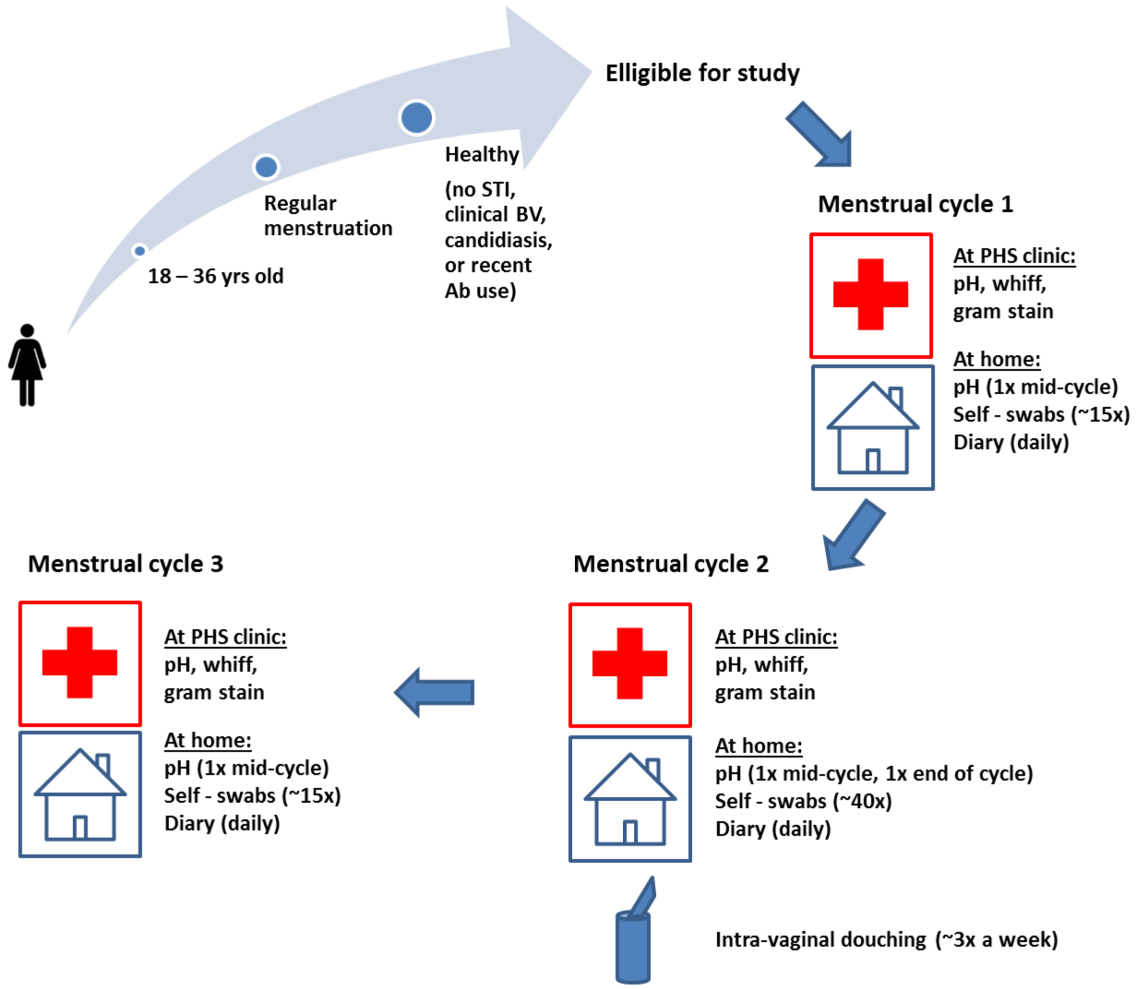

Figure S2 a to e. Participant’s responses in evaluation questionnaire after study completion; n=19 participants.

a. Self reported effect of douching
b. Participants experienced douching as..
c. User friendliness of douching product
d. Would participant use douche in the future?
e. Would participant recommend douching to a friend?

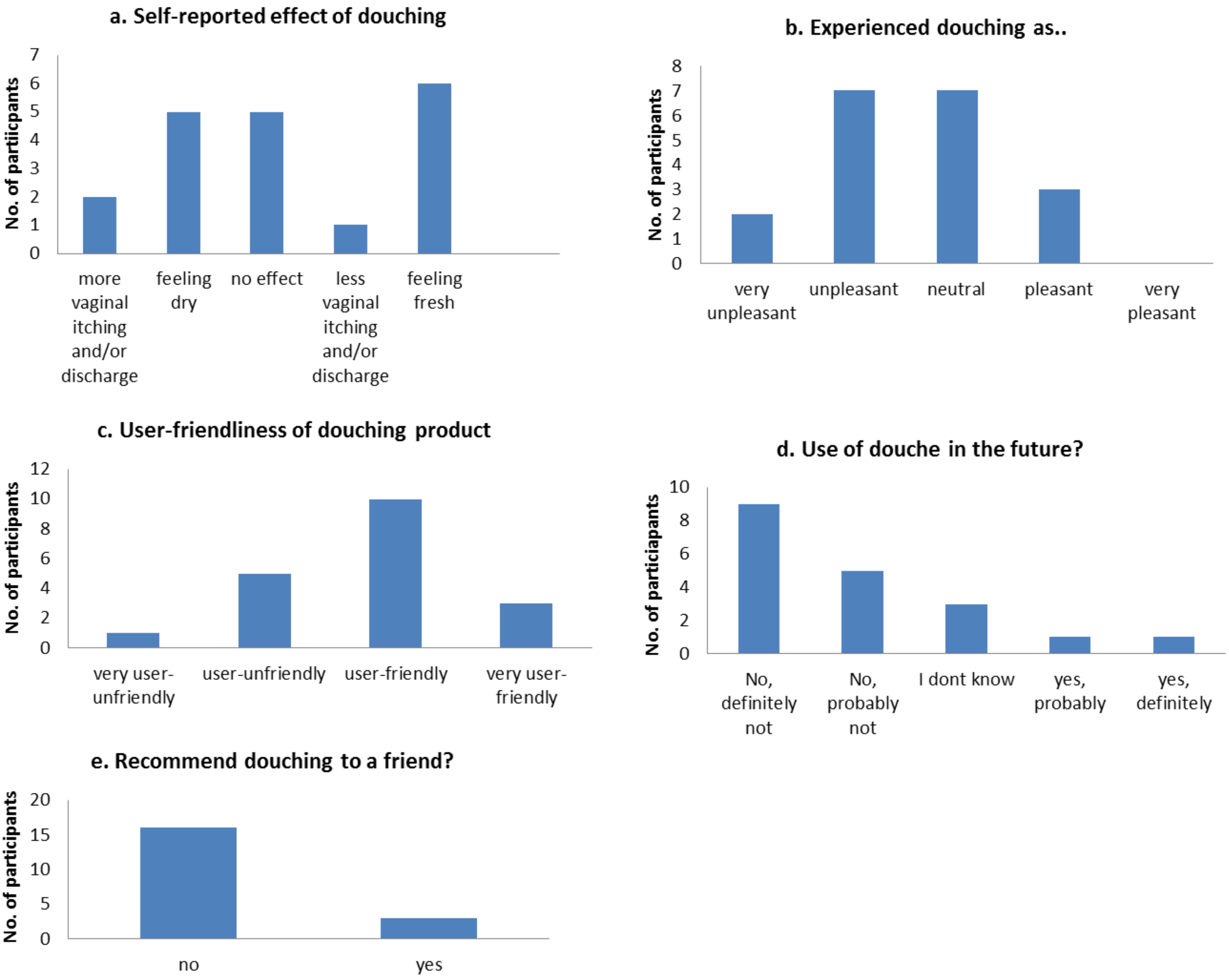

Figure S3. Heatmap depicting microbial species (y-axis) per sample (x-axis) of the total study population of 1061 tested swabs. Microbial relative abundance is illustrated by the color key; the more red a pixel, the higher abundance for that bacterial species. The sidebar above the heatmap depict vaginal microbiome clusters with *k* set to 12.

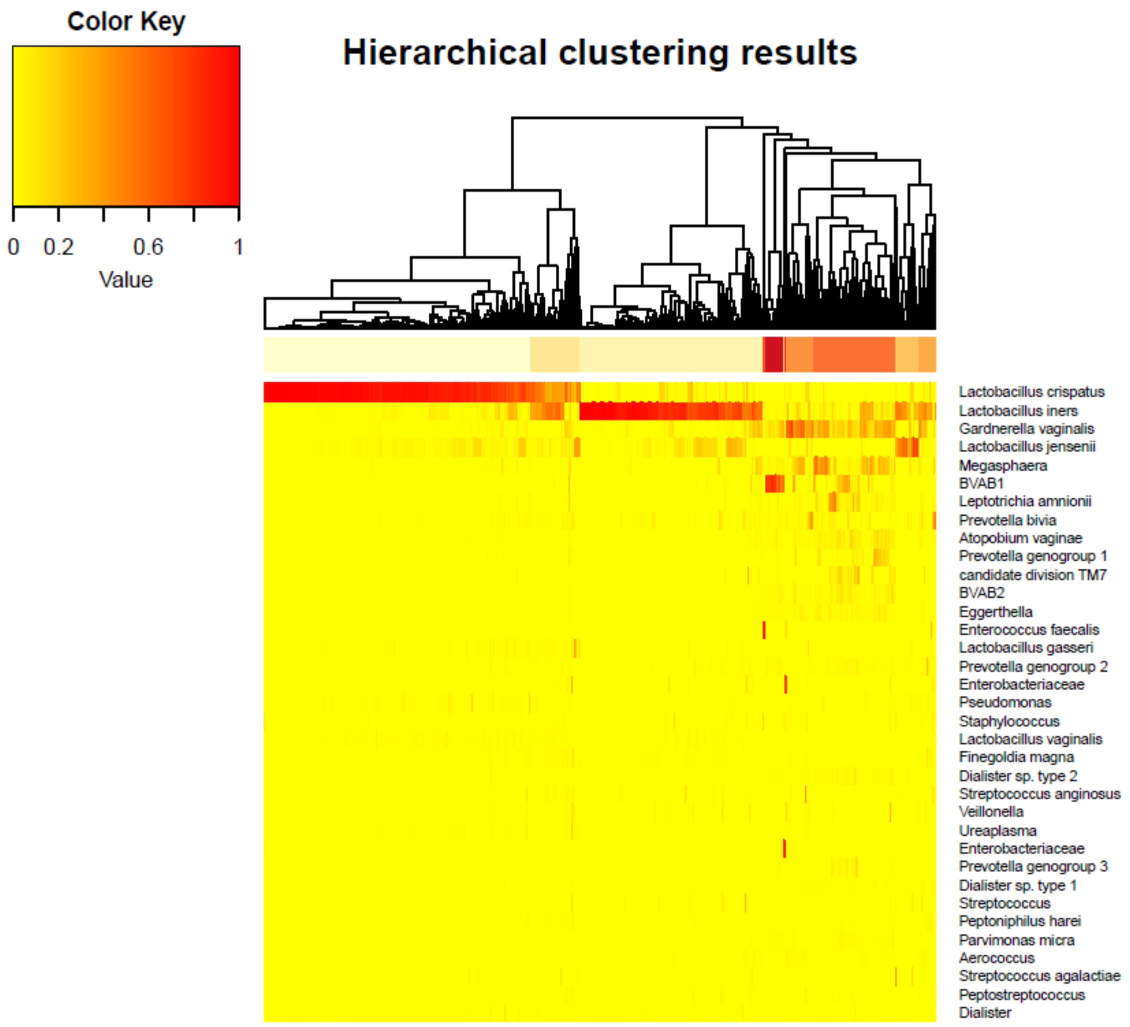

Figure S4. Vaginal microbiota species diversity (Shannon's diversity index) scores per participant per cycle. Boxplots represent median scores with inter quartile range.

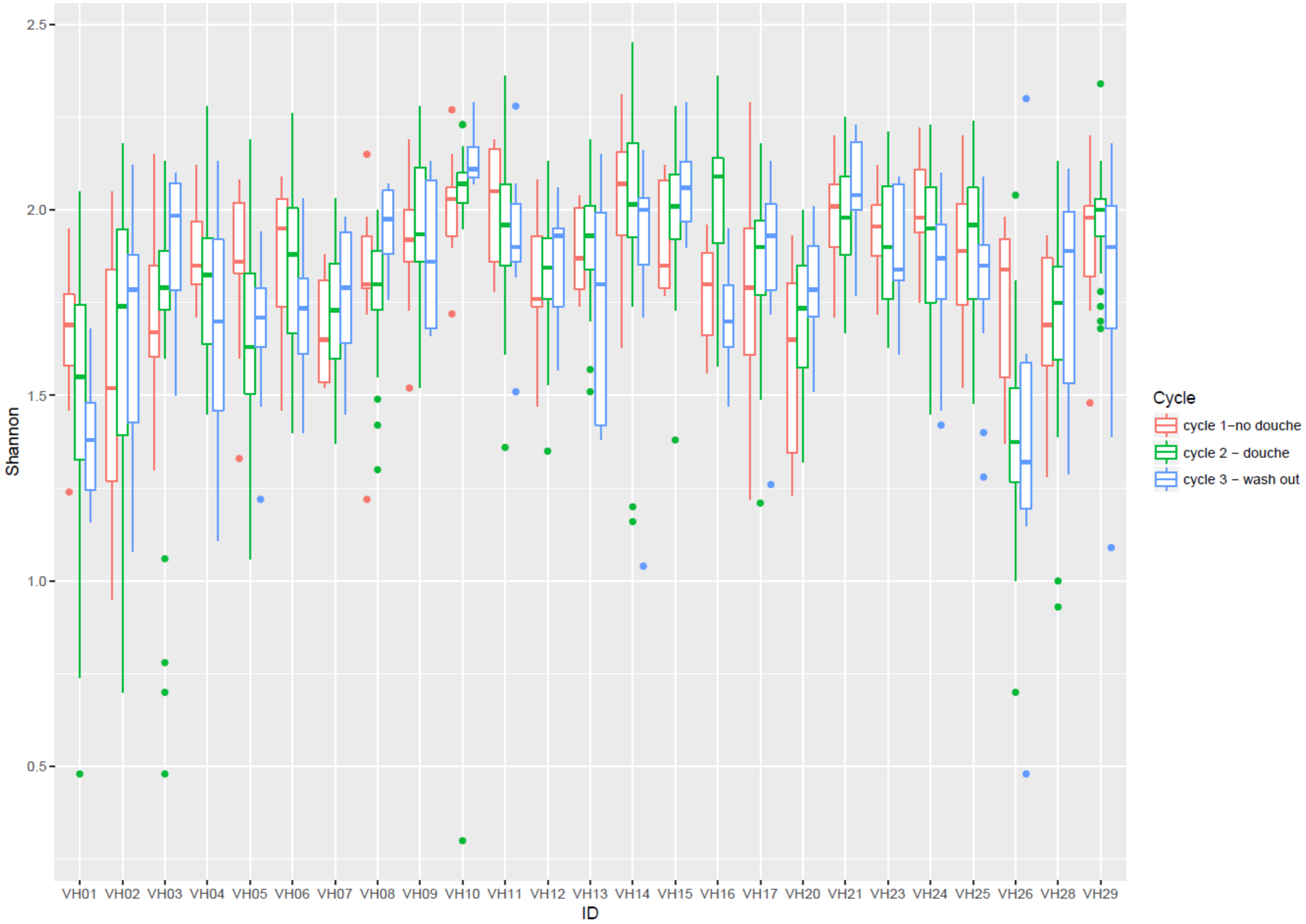

Supplement: Supplementary file 1 — Graphic depictions of study design, questionnaire responses and clustering of vaginal microbiota compositions. (PDF 318 kb) [file 12866_2019_1545_MOESM1_ESM.pdf]
