# Supplementary material for: Effects of an over-the-counter lactic-acid containing intra-vaginal douching product on the vaginal microbiota
Source: BMC Microbiol. 2019 Jul 25;19:168. doi: 10.1186/s12866-019-1545-0 (PMC6659218; doi:10.1186/s12866-019-1545-0)
Supplement: Supplementary file 3 — Heatmaps per participant. (PDF 976 kb) [file 12866_2019_1545_MOESM3_ESM.pdf]

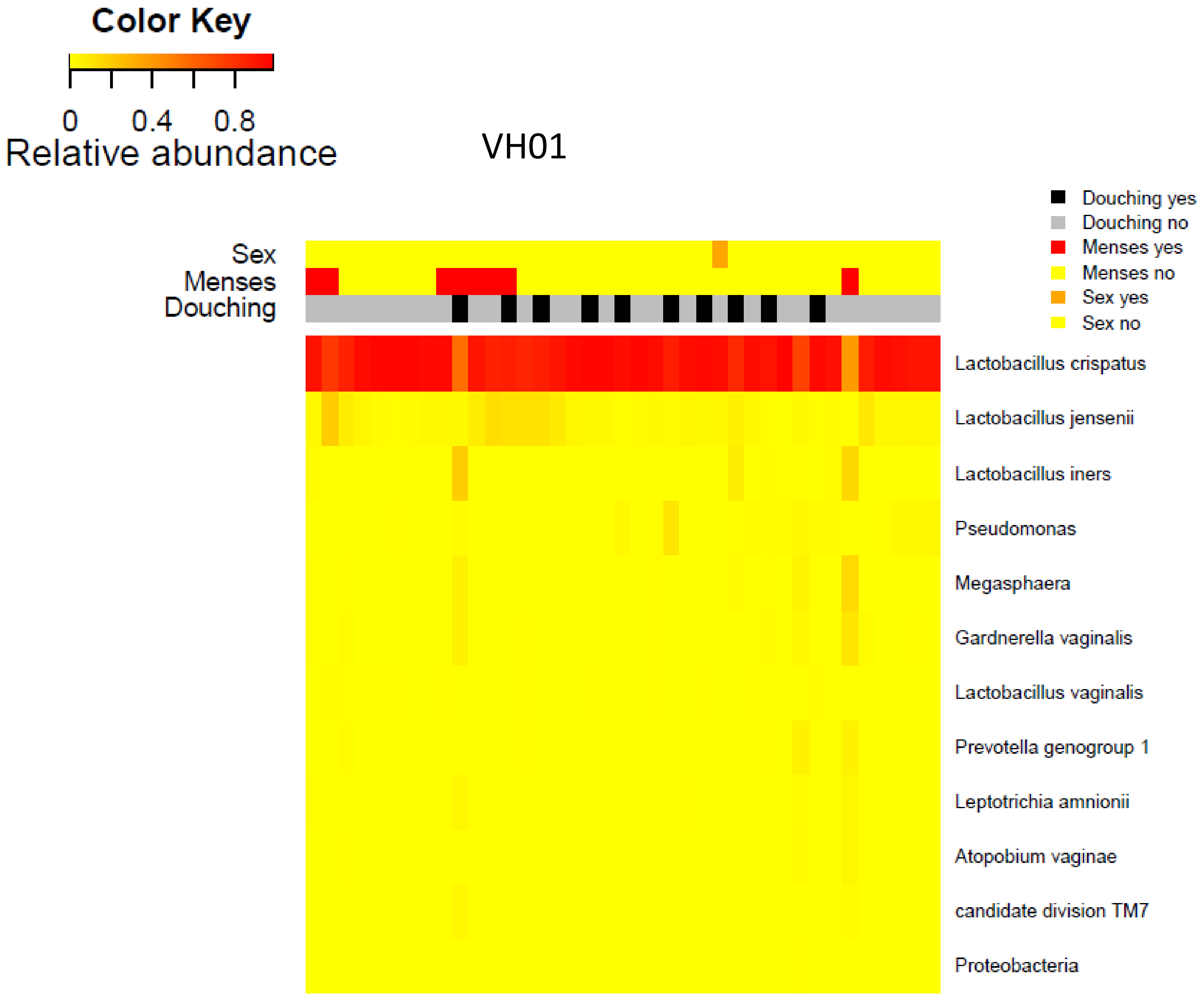

Color Key
0
0.4
0.8
Relative abundance
VH01
Douching yes
Douching no
Menses yes
Menses no
Sex yes
Sex no
Sex
Menses
Douching
Lactobacillus crispatus
Lactobacillus jensenii
Lactobacillus iners
Pseudomonas
Megasphaera
Gardnerella vaginalis
Lactobacillus vaginalis
Prevotella genogroup 1
Leptotrichia amnionii
Atopobium vaginae
candidate division TM7
Proteobacteria

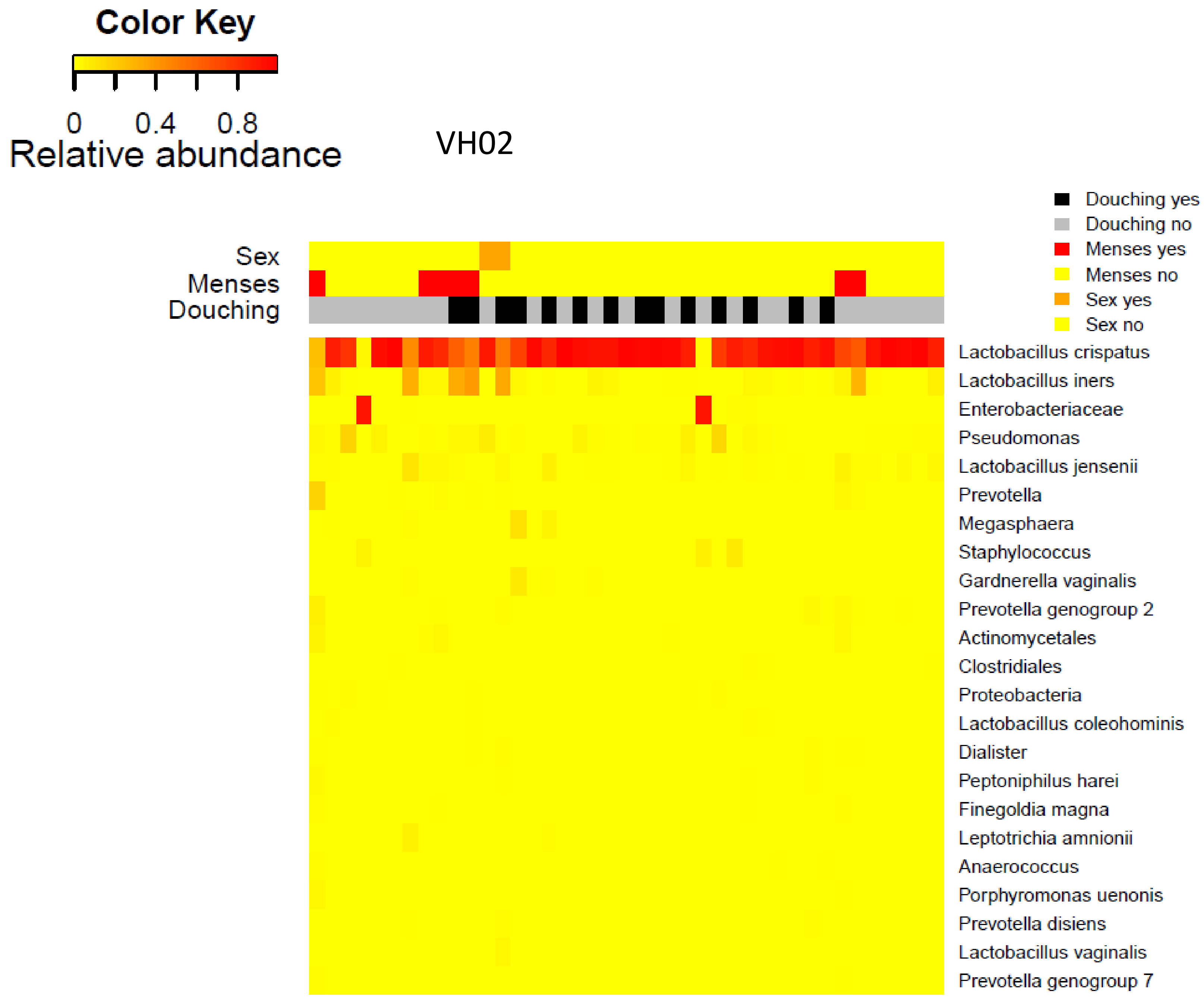

Color Key
0
0.4
0.8
Relative abundance
VH02
Douching yes
Douching no
Menses yes
Menses no
Sex yes
Sex no
Sex
Menses
Douching
Lactobacillus crispatus
Lactobacillus iners
Enterobacteriaceae
Pseudomonas
Lactobacillus jensenii
Prevotella
Megasphaera
Staphylococcus
Gardnerella vaginalis
Prevotella genogroup 2
Actinomycetales
Clostridiales
Proteobacteria
Lactobacillus coleohominis
Dialister
Peptoniphilus harei
Finegoldia magna
Leptotrichia amnionii
Anaerococcus
Porphyromonas uenonis
Prevotella disiens
Lactobacillus vaginalis
Prevotella genogroup 7

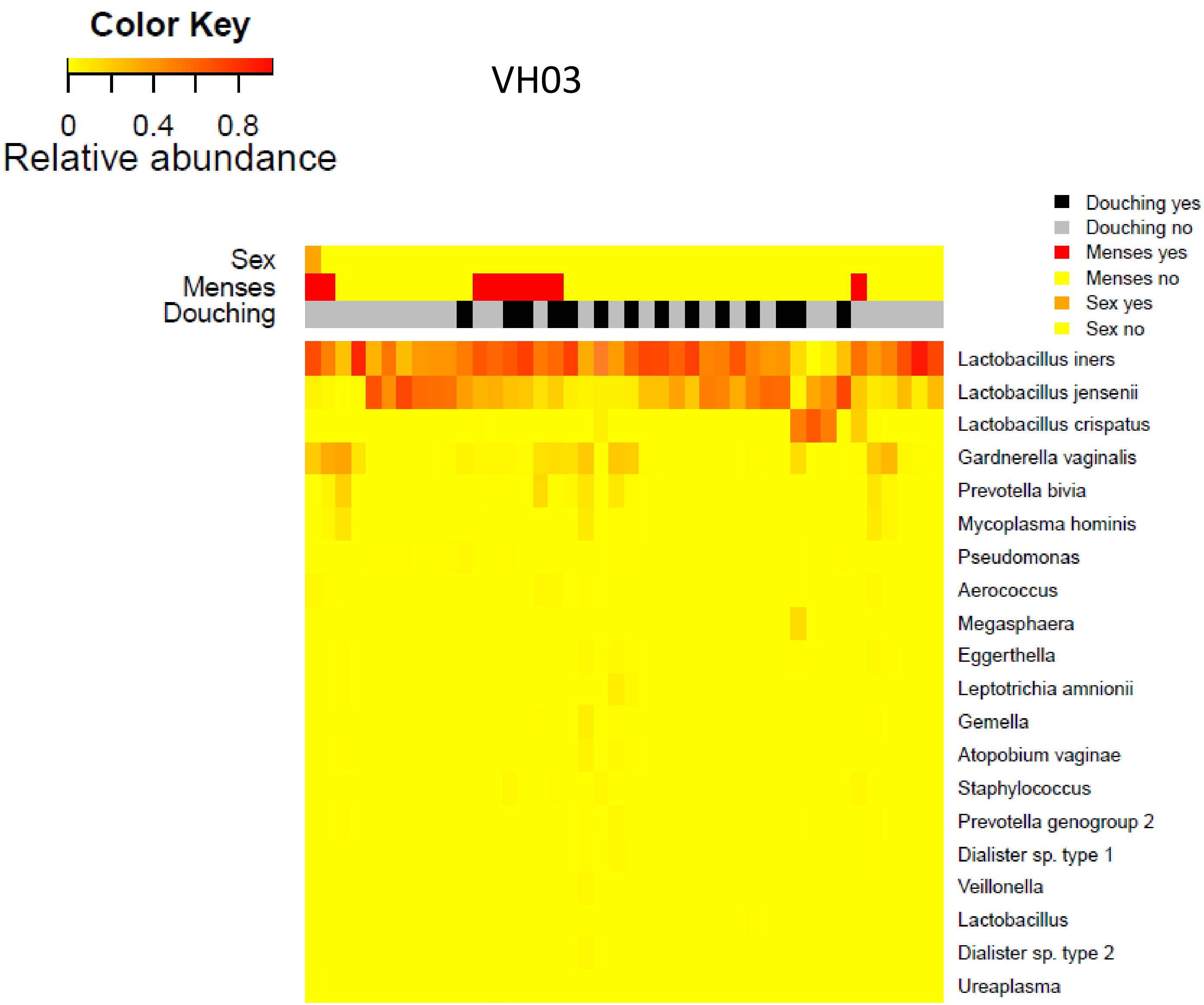
Color Key
0
0.4
0.8
Relative abundance
VH03
Douching yes
Douching no
Menses yes
Menses no
Sex yes
Sex no
Sex
Menses
Douching
Lactobacillus iners
Lactobacillus jensenii
Lactobacillus crispatus
Gardnerella vaginalis
Prevotella bivia
Mycoplasma hominis
Pseudomonas
Aerococcus
Megasphaera
Eggerthella
Leptotrichia amnionii
Gemella
Atopobium vaginae
Staphylococcus
Prevotella genogroup 2
Dialister sp. type 1
Veillonella
Lactobacillus
Dialister sp. type 2
Ureaplasma

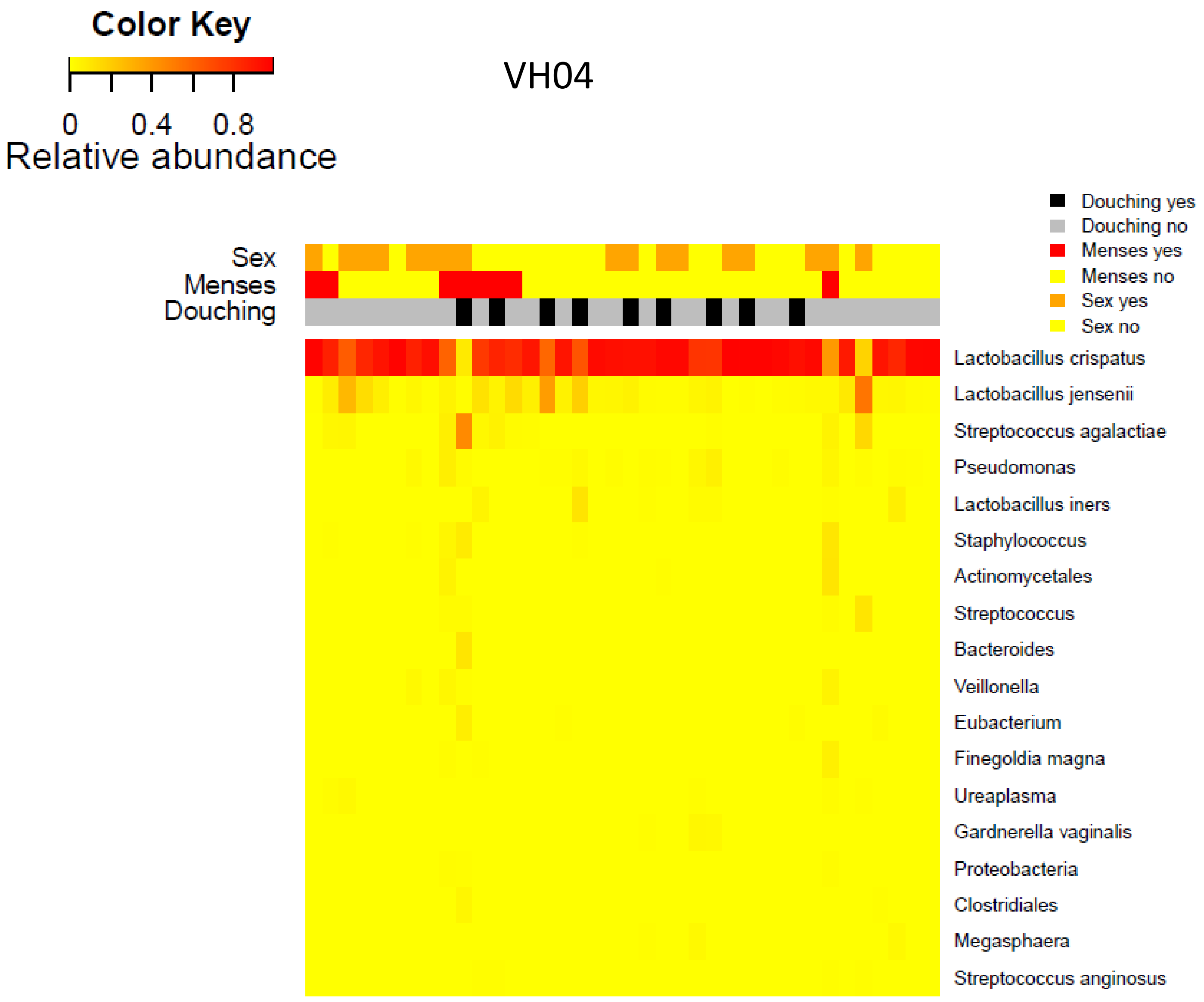

Color Key
0
0.4
0.8
Relative abundance
VH04
Douching yes
Douching no
Menses yes
Menses no
Sex yes
Sex no
Sex
Menses
Douching
Lactobacillus crispatus
Lactobacillus jensenii
Streptococcus agalactiae
Pseudomonas
Lactobacillus iners
Staphylococcus
Actinomycetales
Streptococcus
Bacteroides
Veillonella
Eubacterium
Finegoldia magna
Ureaplasma
Gardnerella vaginalis
Proteobacteria
Clostridiales
Megasphaera
Streptococcus anginosus

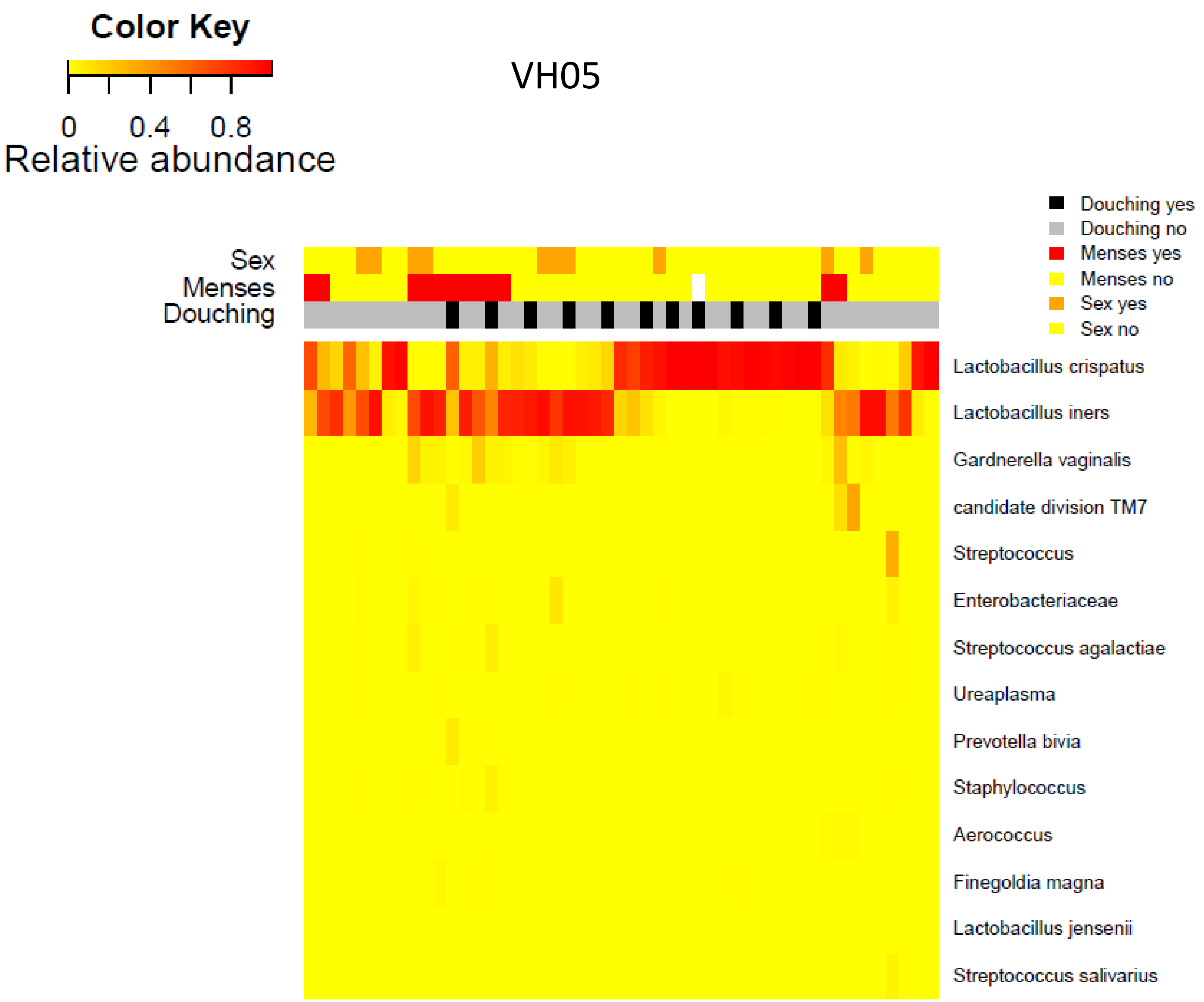
Color Key
0
0.4
0.8
Relative abundance
VH05
Douching yes
Douching no
Menses yes
Menses no
Sex yes
Sex no
Sex
Menses
Douching
Lactobacillus crispatus
Lactobacillus iners
Gardnerella vaginalis
candidate division TM7
Streptococcus
Enterobacteriaceae
Streptococcus agalactiae
Ureaplasma
Prevotella bivia
Staphylococcus
Aerococcus
Finegoldia magna
Lactobacillus jensenii
Streptococcus salivarius

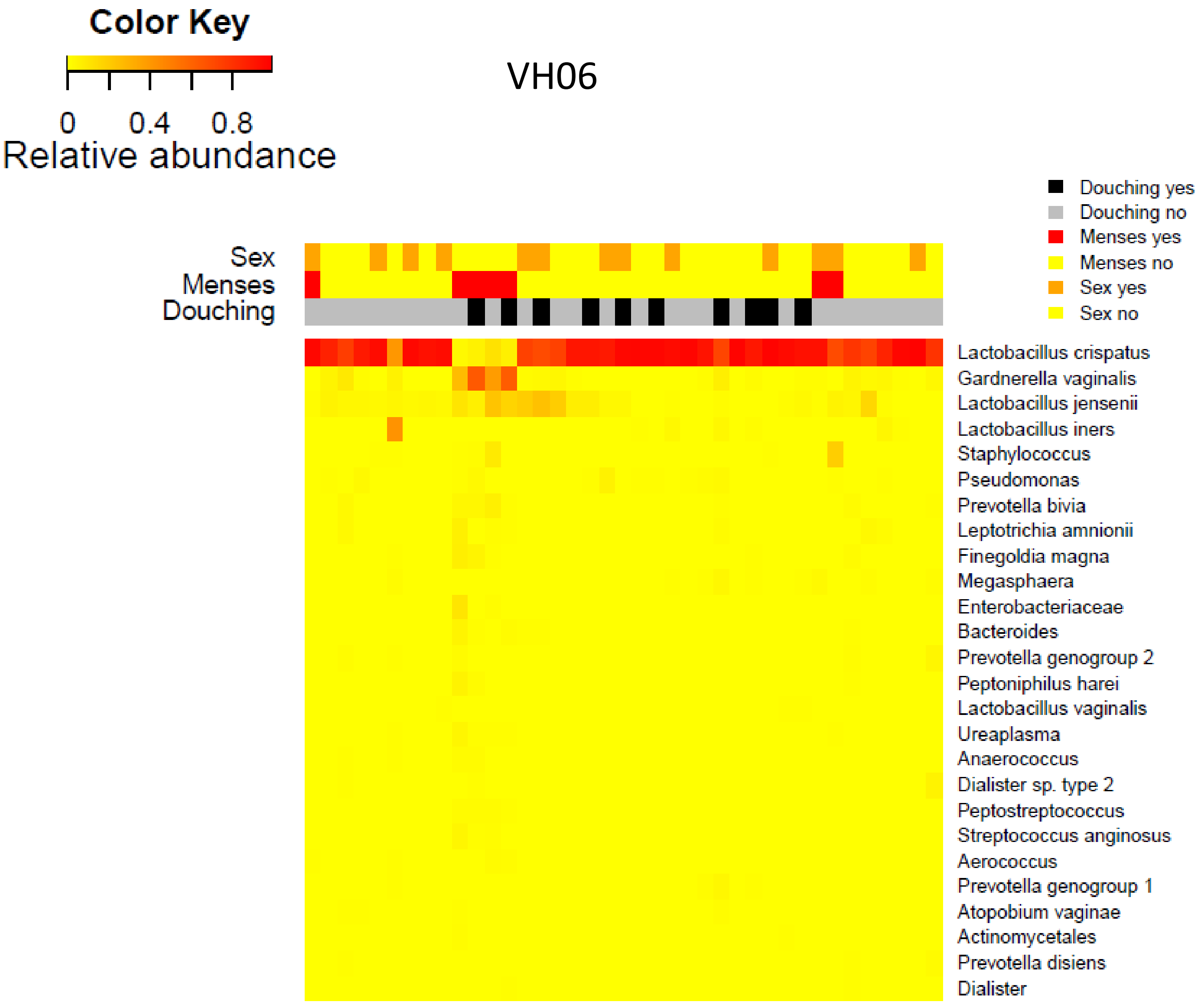

Color Key
0
0.4
0.8
Relative abundance
VH06
Douching yes
Douching no
Menses yes
Menses no
Sex yes
Sex no
Sex
Menses
Douching
Lactobacillus crispatus
Gardnerella vaginalis
Lactobacillus jensenii
Lactobacillus iners
Staphylococcus
Pseudomonas
Prevotella bivia
Leptotrichia amnionii
Finegoldia magna
Megasphaera
Enterobacteriaceae
Bacteroides
Prevotella genogroup 2
Peptoniphilus harei
Lactobacillus vaginalis
Ureaplasma
Anaerococcus
Dialister sp. type 2
Peptostreptococcus
Streptococcus anginosus
Aerococcus
Prevotella genogroup 1
Atopobium vaginae
Actinomycetales
Prevotella disiens
Dialister

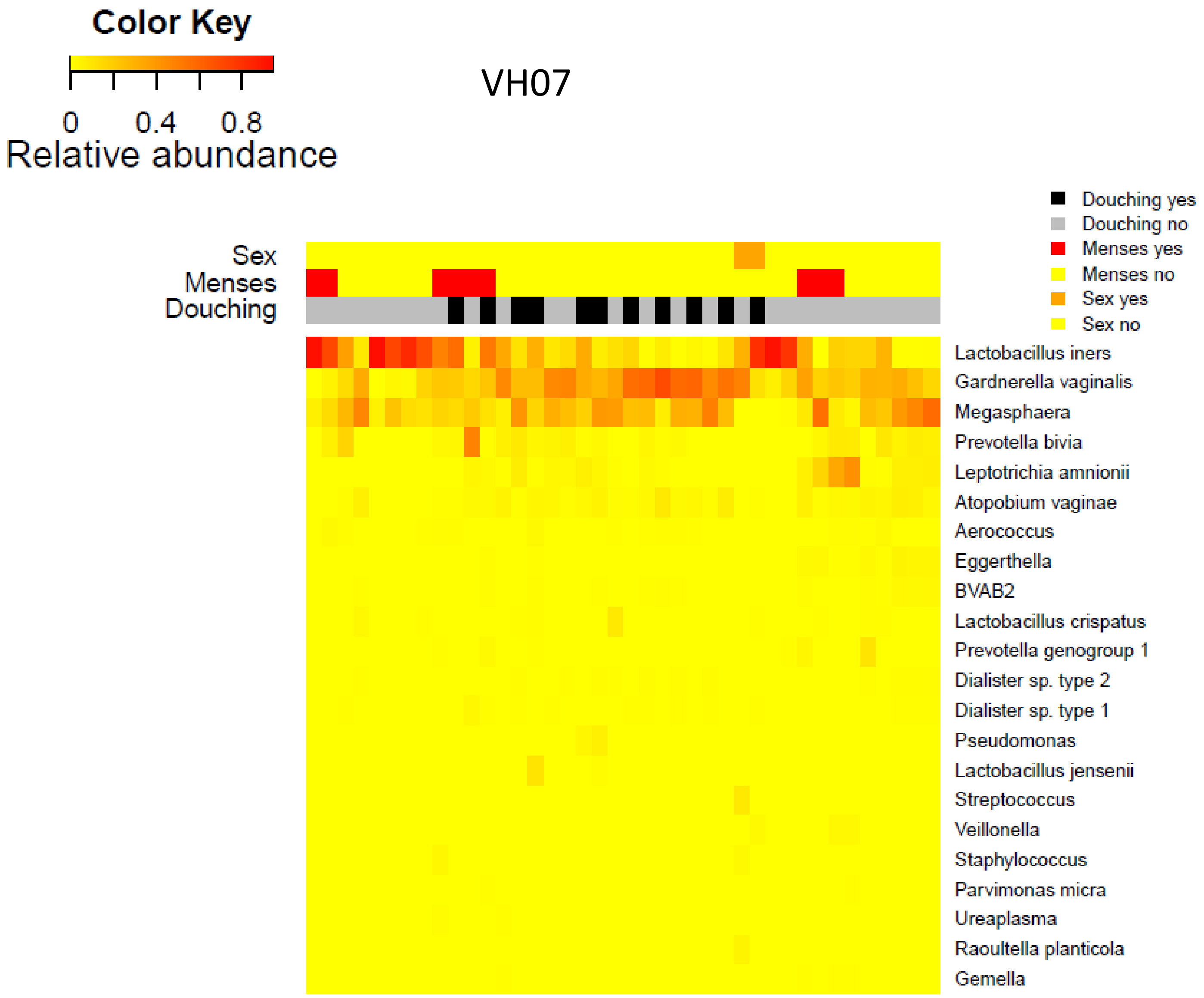
Color Key
0
0.4
0.8
Relative abundance
VH07
Douching yes
Douching no
Menses yes
Menses no
Sex yes
Sex no
Sex
Menses
Douching
Lactobacillus iners
Gardnerella vaginalis
Megasphaera
Prevotella bivia
Leptotrichia amnionii
Atopobium vaginae
Aerococcus
Eggerthella
BVAB2
Lactobacillus crispatus
Prevotella genogroup 1
Dialister sp. type 2
Dialister sp. type 1
Pseudomonas
Lactobacillus jensenii
Streptococcus
Veillonella
Staphylococcus
Parvimonas micra
Ureaplasma
Raoultella planticola
Gemella

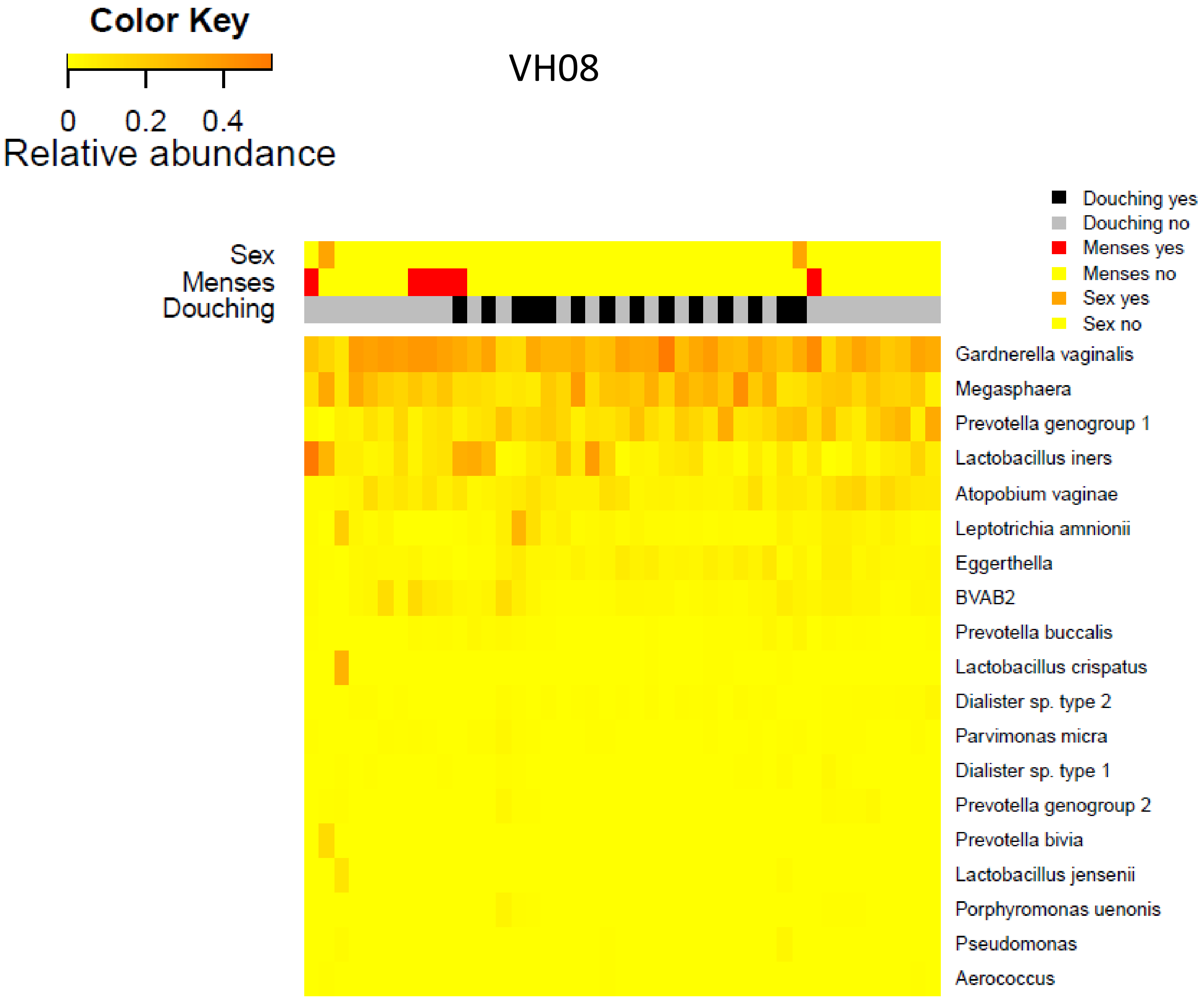

Color Key
0
0.2
0.4
Relative abundance
VH08
Douching yes
Douching no
Menses yes
Menses no
Sex yes
Sex no
Sex
Menses
Douching
Gardnerella vaginalis
Megasphaera
Prevotella genogroup 1
Lactobacillus iners
Atopobium vaginae
Leptotrichia amnionii
Eggerthella
BVAB2
Prevotella buccalis
Lactobacillus crispatus
Dialister sp. type 2
Parvimonas micra
Dialister sp. type 1
Prevotella genogroup 2
Prevotella bivia
Lactobacillus jensenii
Porphyromonas uenonis
Pseudomonas
Aerococcus

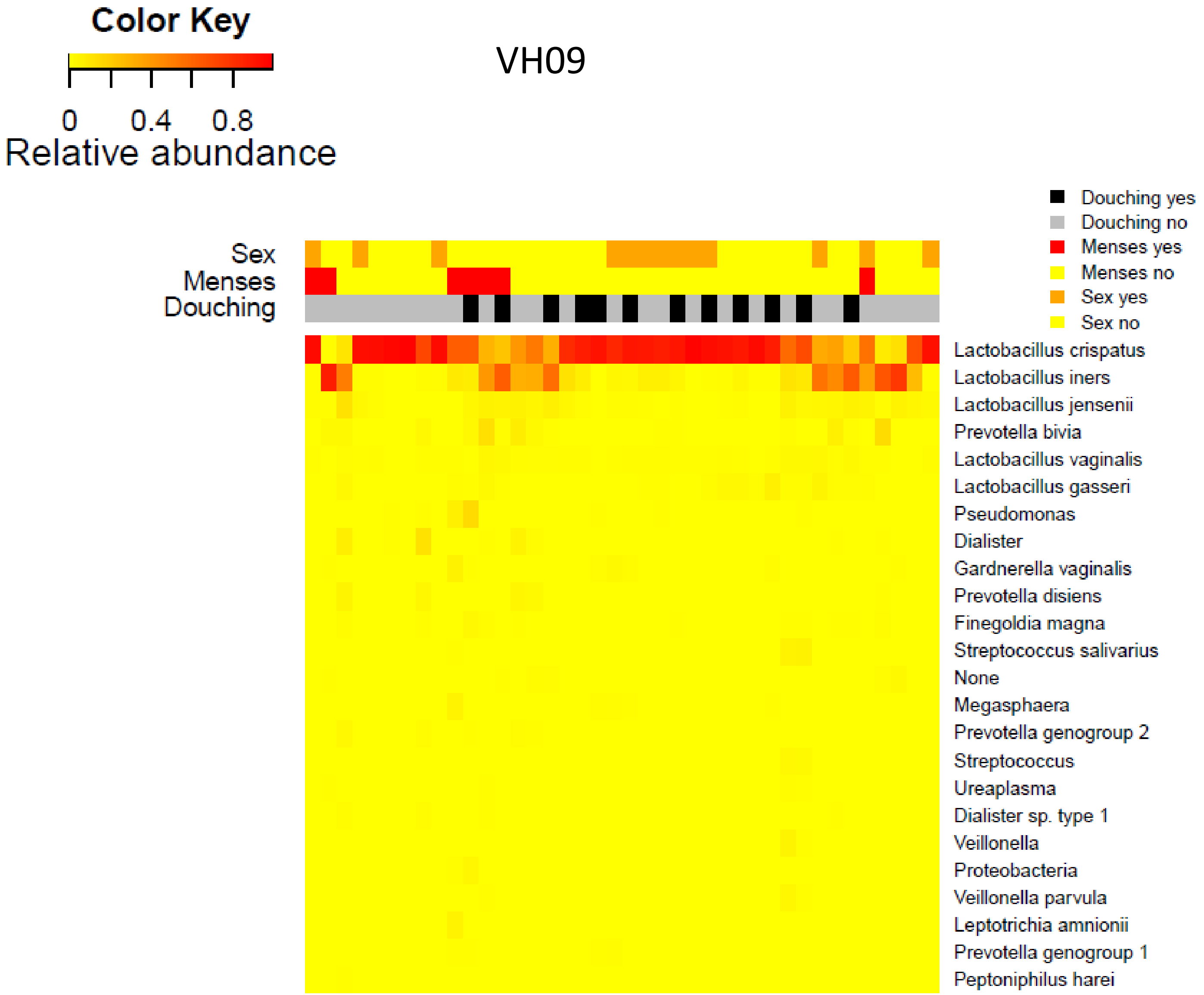

Color Key
0
0.4
0.8
Relative abundance
VH09
Douching yes
Douching no
Menses yes
Menses no
Sex yes
Sex no
Sex
Menses
Douching
Lactobacillus crispatus
Lactobacillus iners
Lactobacillus jensenii
Prevotella bivia
Lactobacillus vaginalis
Lactobacillus gasseri
Pseudomonas
Dialister
Gardnerella vaginalis
Prevotella disiens
Finegoldia magna
Streptococcus salivarius
None
Megasphaera
Prevotella genogroup 2
Streptococcus
Ureaplasma
Dialister sp. type 1
Veillonella
Proteobacteria
Veillonella parvula
Leptotrichia amnionii
Prevotella genogroup 1
Peptoniphilus harei

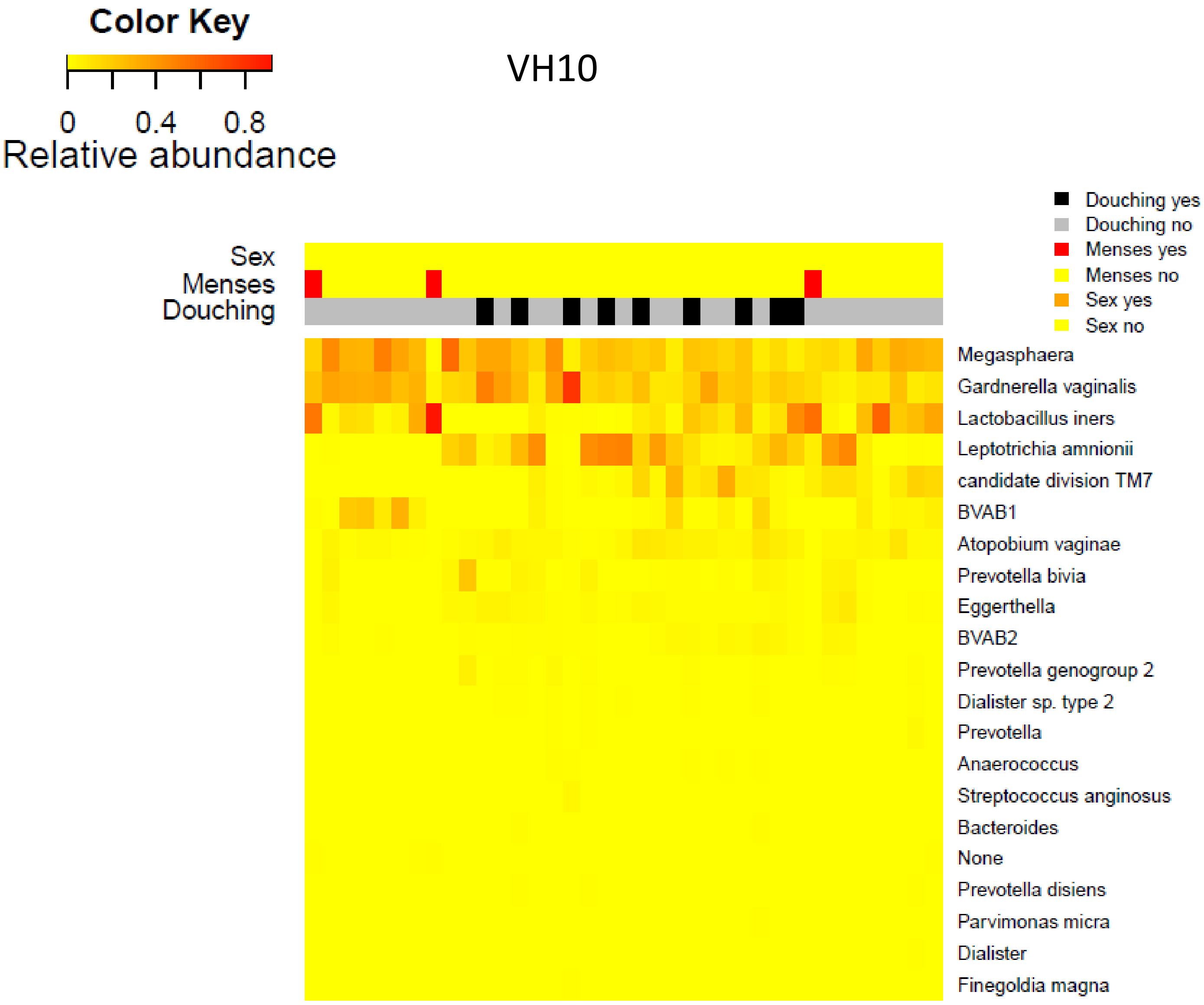
Color Key
0
0.4
0.8
Relative abundance
VH10
Sex
Menses
Douching
Douching yes
Douching no
Menses yes
Menses no
Sex yes
Sex no
Megasphaera
Gardnerella vaginalis
Lactobacillus iners
Leptotrichia amnionii
candidate division TM7
BVAB1
Atopobium vaginae
Prevotella bivia
Eggerthella
BVAB2
Prevotella genogroup 2
Dialister sp. type 2
Prevotella
Anaerococcus
Streptococcus anginosus
Bacteroides
None
Prevotella disiens
Parvimonas micra
Dialister
Finegoldia magna

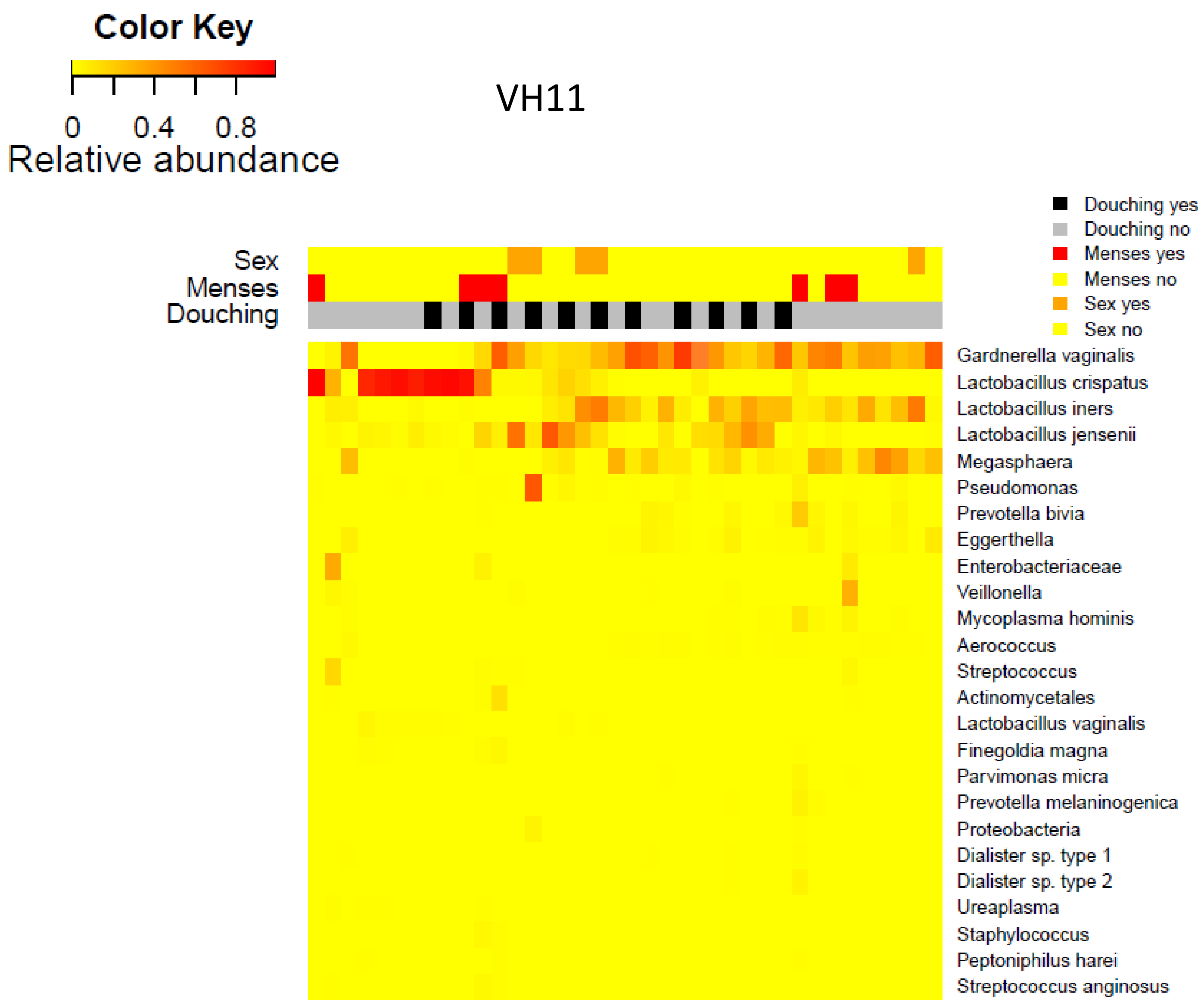
Color Key
0
0.4
0.8
Relative abundance
VH11
Douching yes
Douching no
Menses yes
Menses no
Sex yes
Sex no
Sex
Menses
Douching
Gardnerella vaginalis
Lactobacillus crispatus
Lactobacillus iners
Lactobacillus jensenii
Megasphaera
Pseudomonas
Prevotella bivia
Eggerthella
Enterobacteriaceae
Veillonella
Mycoplasma hominis
Aerococcus
Streptococcus
Actinomycetales
Lactobacillus vaginalis
Finegoldia magna
Parvimonas micra
Prevotella melaninogenica
Proteobacteria
Dialister sp. type 1
Dialister sp. type 2
Ureaplasma
Staphylococcus
Peptoniphilus harei
Streptococcus anginosus

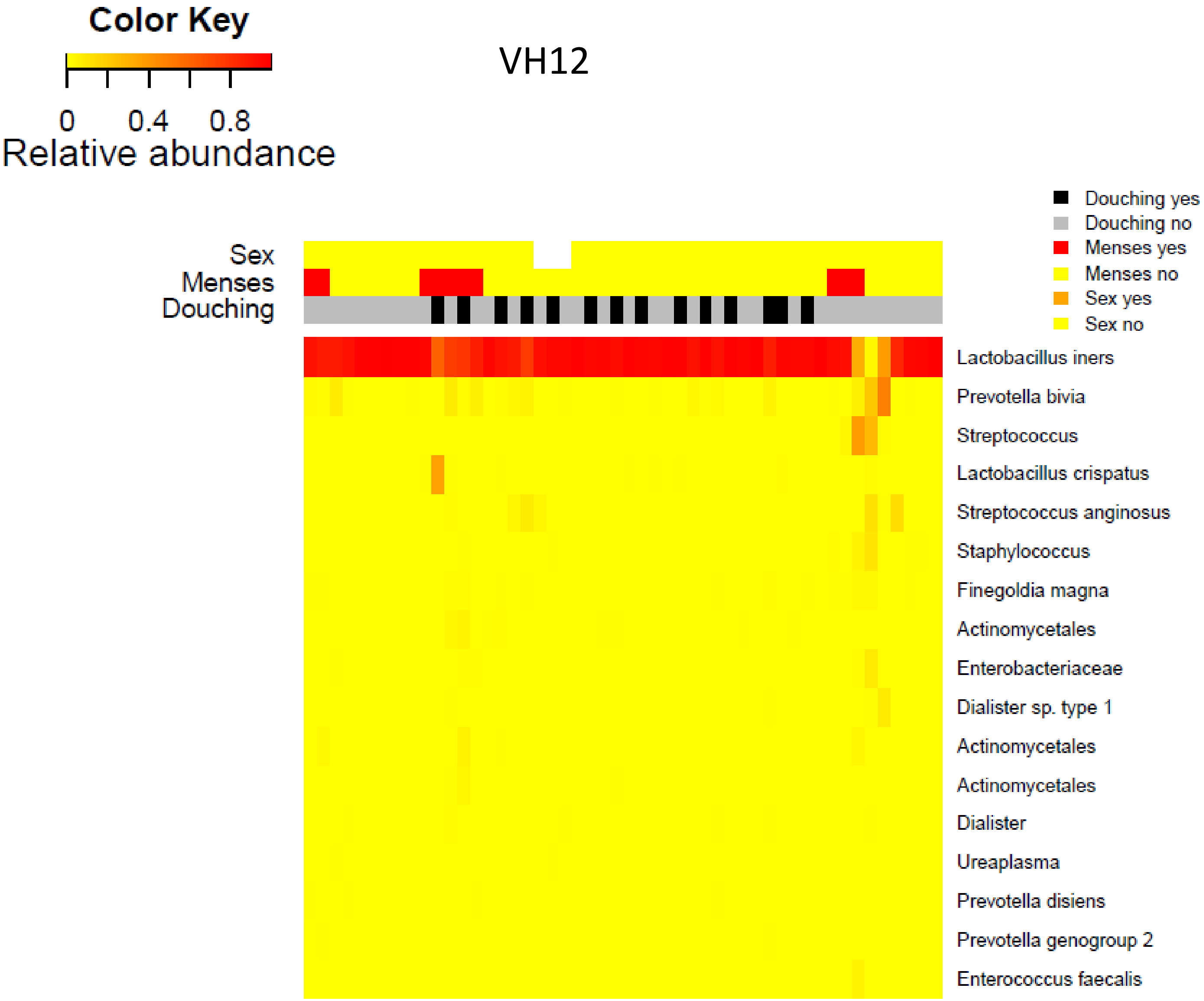

Color Key
0
0.4
0.8
Relative abundance
VH12
Douching yes
Douching no
Menses yes
Menses no
Sex yes
Sex no
Sex
Menses
Douching
Lactobacillus iners
Prevotella bivia
Streptococcus
Lactobacillus crispatus
Streptococcus anginosus
Staphylococcus
Finegoldia magna
Actinomycetales
Enterobacteriaceae
Dialister sp. type 1
Actinomycetales
Actinomycetales
Dialister
Ureaplasma
Prevotella disiens
Prevotella genogroup 2
Enterococcus faecalis

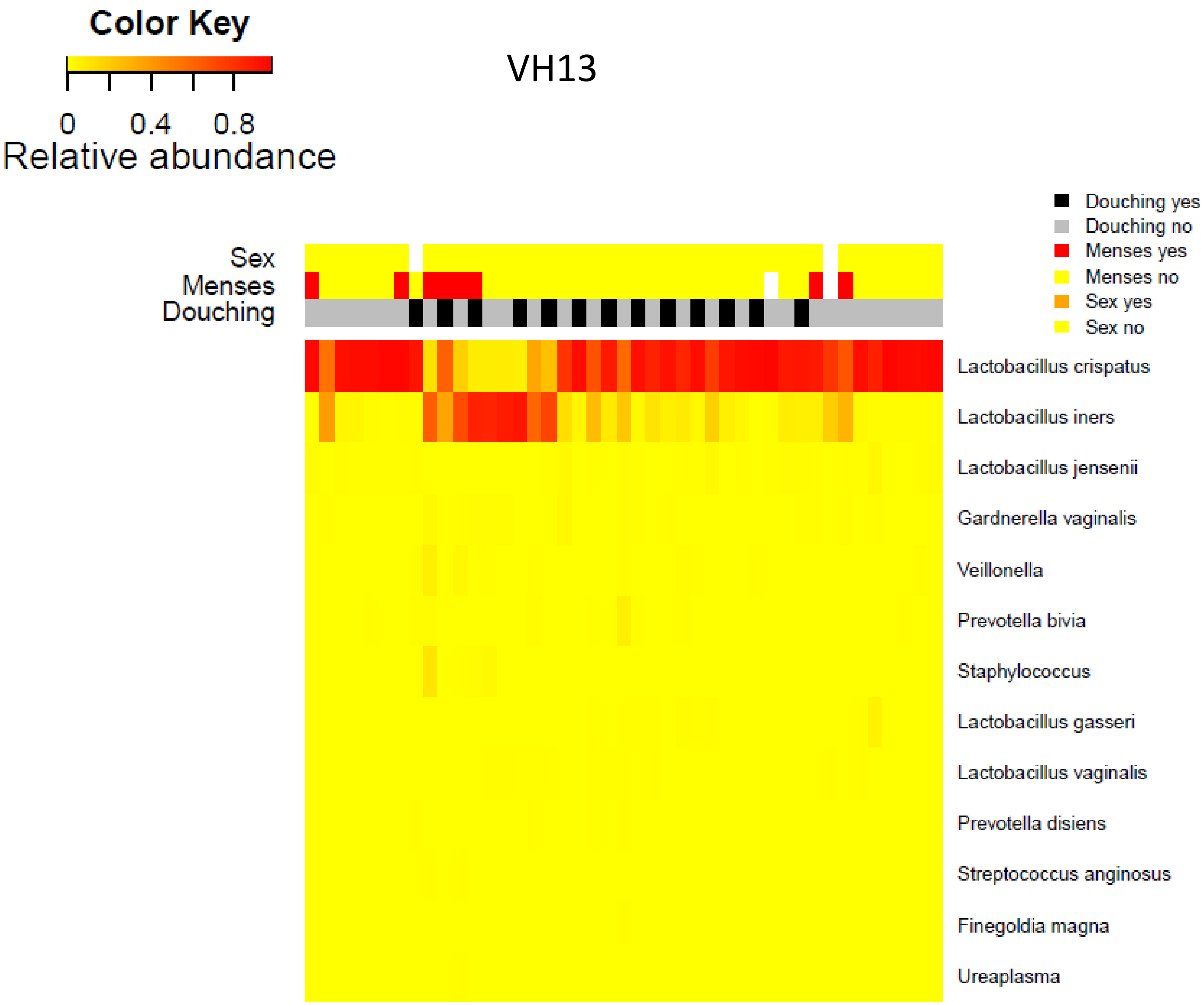
Color Key
0
0.4
0.8
Relative abundance
VH13
Douching yes
Douching no
Menses yes
Menses no
Sex yes
Sex no
Sex
Menses
Douching
Lactobacillus crispatus
Lactobacillus iners
Lactobacillus jensenii
Gardnerella vaginalis
Veillonella
Prevotella bivia
Staphylococcus
Lactobacillus gasseri
Lactobacillus vaginalis
Prevotella disiens
Streptococcus anginosus
Finegoldia magna
Ureaplasma

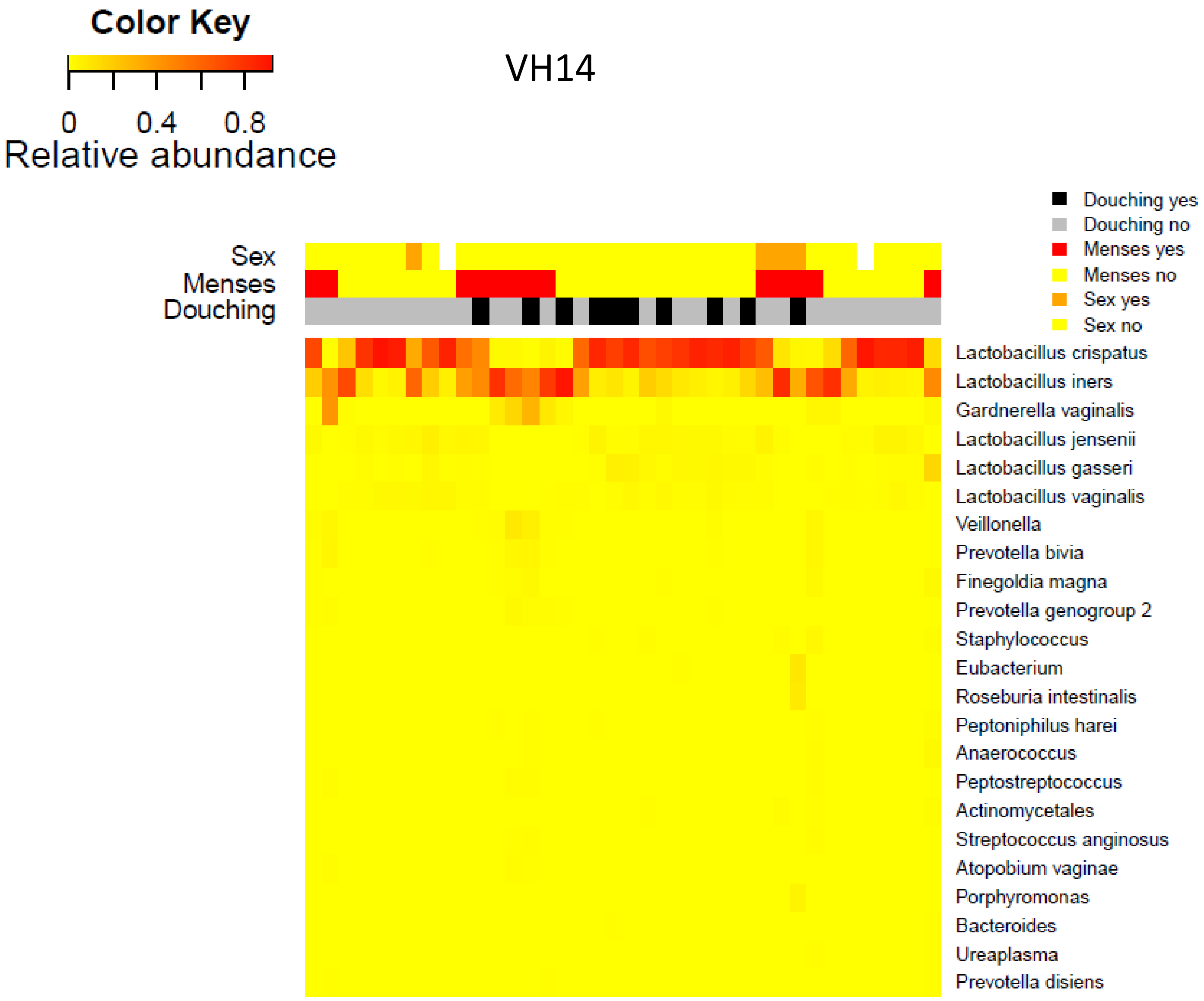

Color Key
0
0.4
0.8
Relative abundance
VH14
Douching yes
Douching no
Menses yes
Menses no
Sex yes
Sex no
Sex
Menses
Douching
Lactobacillus crispatus
Lactobacillus iners
Gardnerella vaginalis
Lactobacillus jensenii
Lactobacillus gasseri
Lactobacillus vaginalis
Veillonella
Prevotella bivia
Finegoldia magna
Prevotella genogroup 2
Staphylococcus
Eubacterium
Roseburia intestinalis
Peptoniphilus harei
Anaerococcus
Peptostreptococcus
Actinomycetales
Streptococcus anginosus
Atopobium vaginae
Porphyromonas
Bacteroides
Ureaplasma
Prevotella disiens

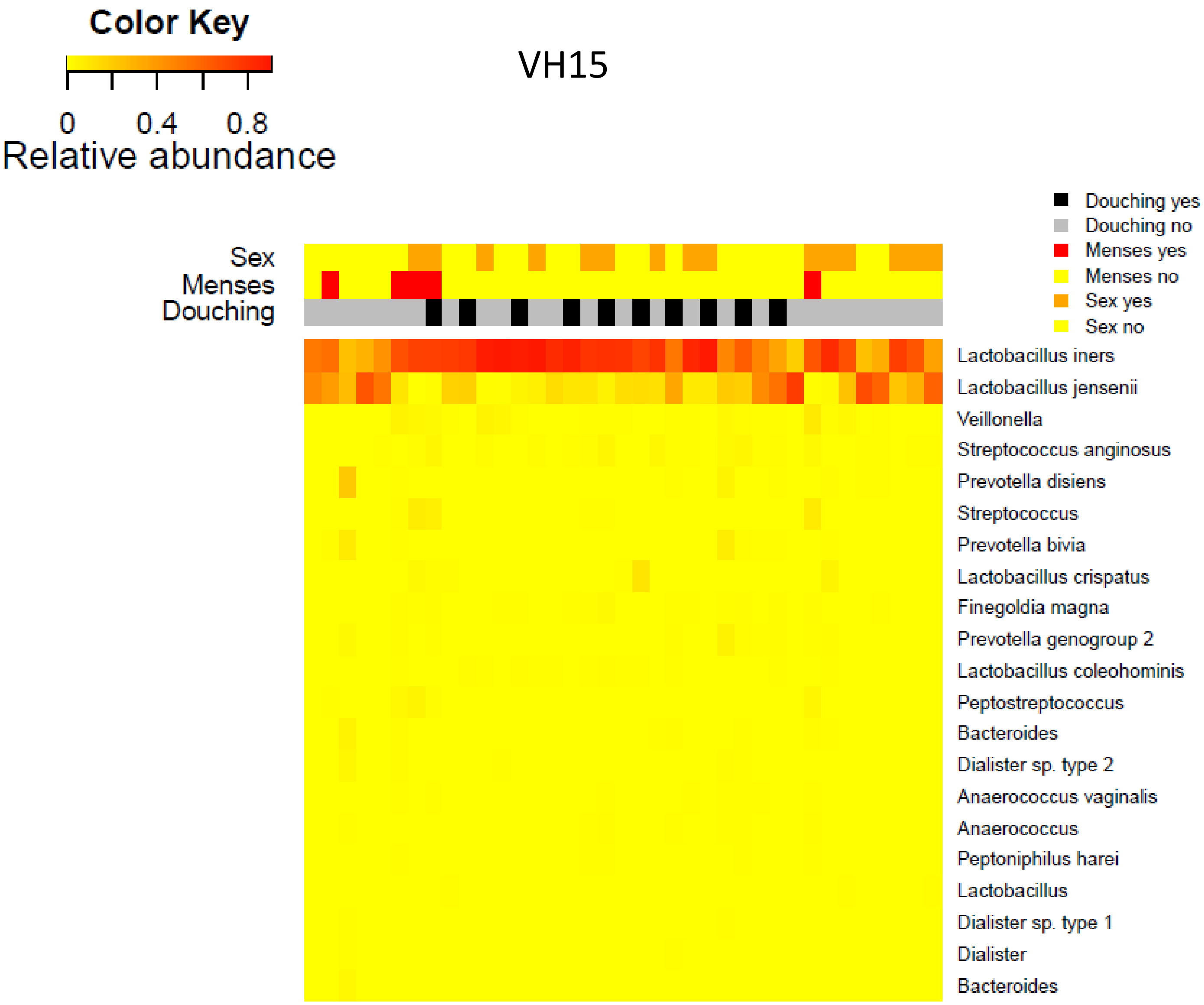
Color Key
0
0.4
0.8
Relative abundance
VH15
Douching yes
Douching no
Menses yes
Menses no
Sex yes
Sex no
Sex
Menses
Douching
Lactobacillus iners
Lactobacillus jensenii
Veillonella
Streptococcus anginosus
Prevotella disiens
Streptococcus
Prevotella bivia
Lactobacillus crispatus
Finegoldia magna
Prevotella genogroup 2
Lactobacillus coleohominis
Peptostreptococcus
Bacteroides
Dialister sp. type 2
Anaerococcus vaginalis
Anaerococcus
Peptoniphilus harei
Lactobacillus
Dialister sp. type 1
Dialister
Bacteroides

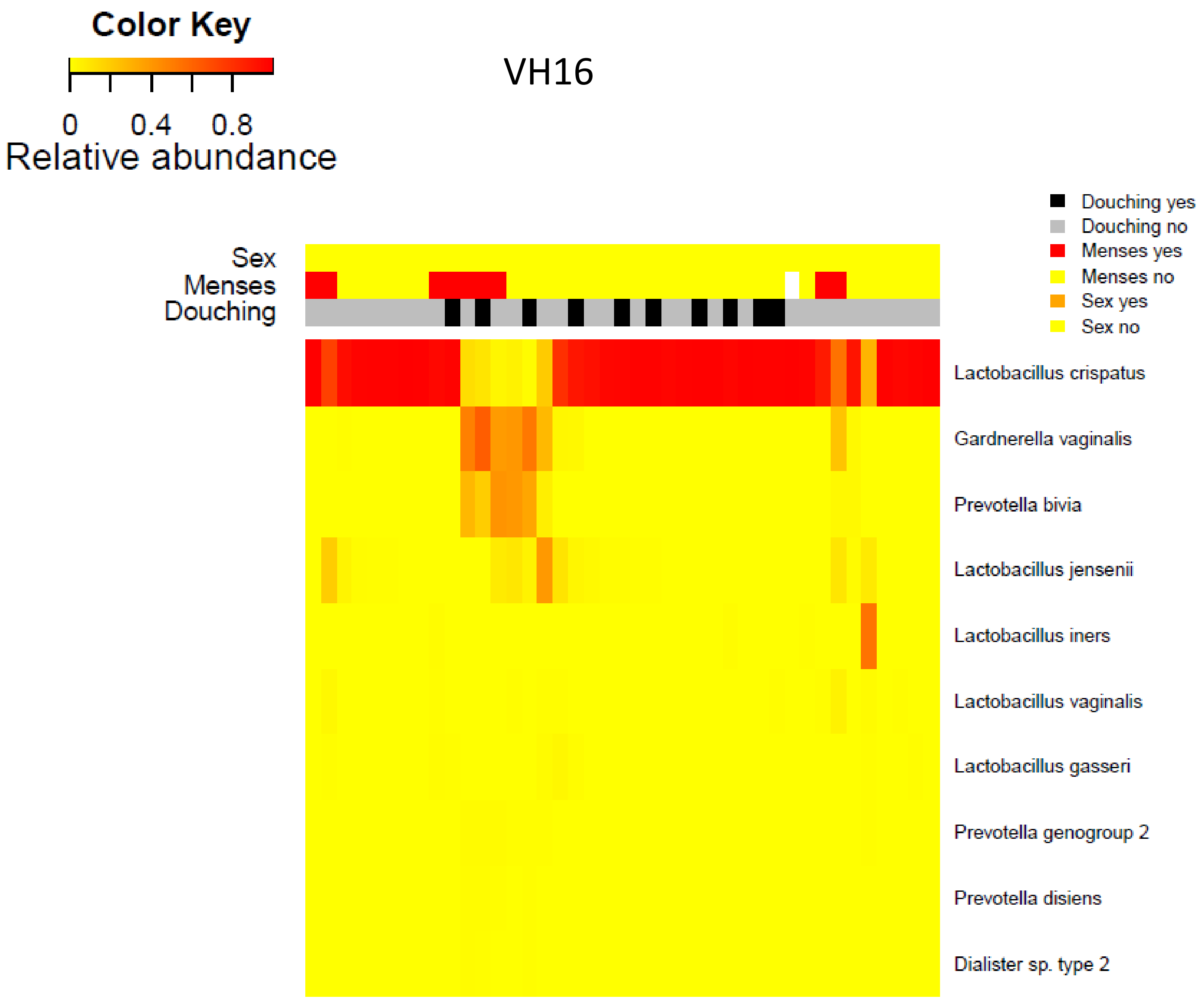

Color Key
0
0.4
0.8
Relative abundance
VH16
Douching yes
Douching no
Menses yes
Menses no
Sex yes
Sex no
Sex
Menses
Douching
Lactobacillus crispatus
Gardnerella vaginalis
Prevotella bivia
Lactobacillus jensenii
Lactobacillus iners
Lactobacillus vaginalis
Lactobacillus gasseri
Prevotella genogroup 2
Prevotella disiens
Dialister sp. type 2

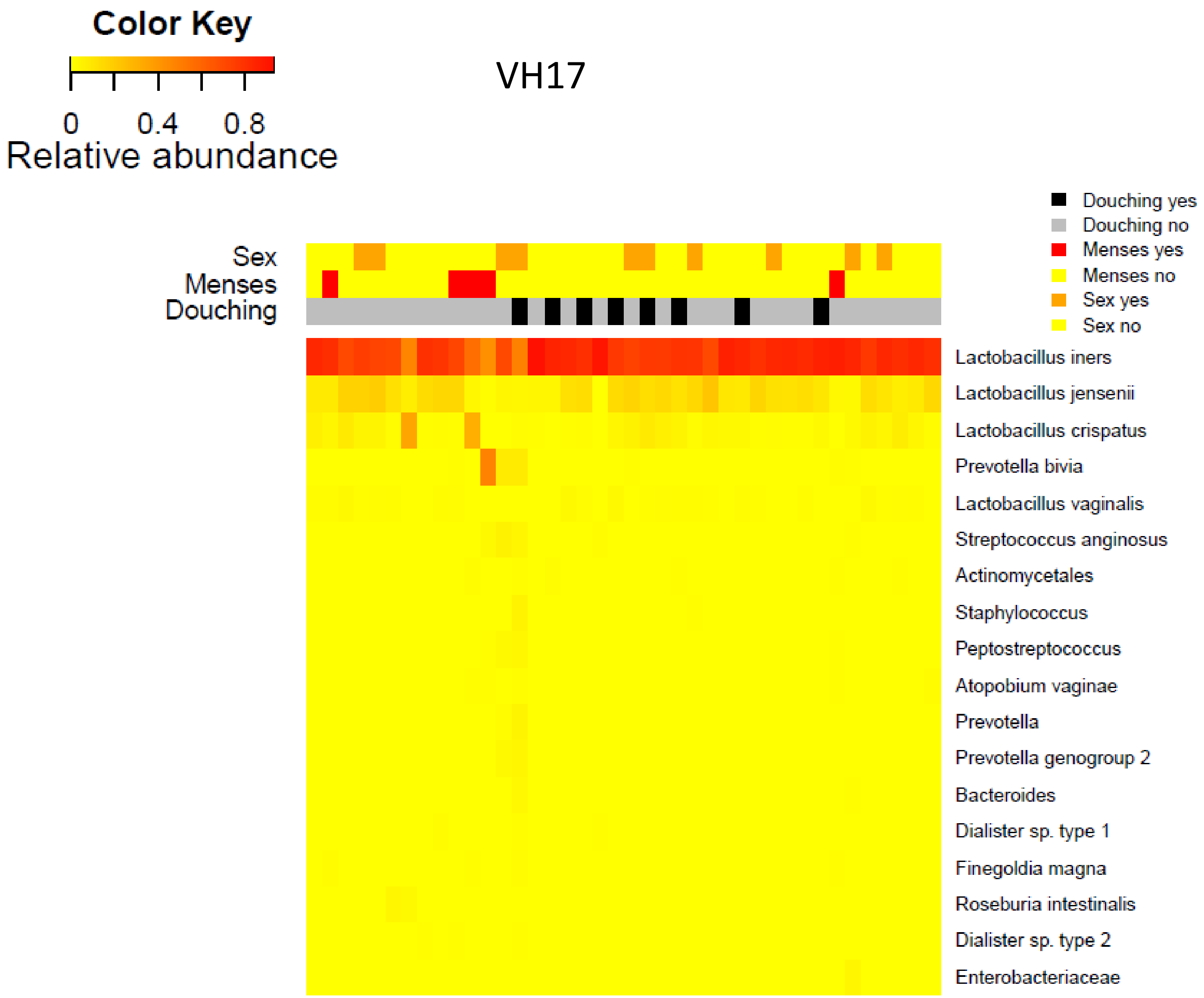
Color Key
0
0.4
0.8
Relative abundance
VH17
Sex
Menses
Douching
Douching yes
Douching no
Menses yes
Menses no
Sex yes
Sex no
Lactobacillus iners
Lactobacillus jensenii
Lactobacillus crispatus
Prevotella bivia
Lactobacillus vaginalis
Streptococcus anginosus
Actinomycetales
Staphylococcus
Peptostreptococcus
Atopobium vaginae
Prevotella
Prevotella genogroup 2
Bacteroides
Dialister sp. type 1
Finegoldia magna
Roseburia intestinalis
Dialister sp. type 2
Enterobacteriaceae

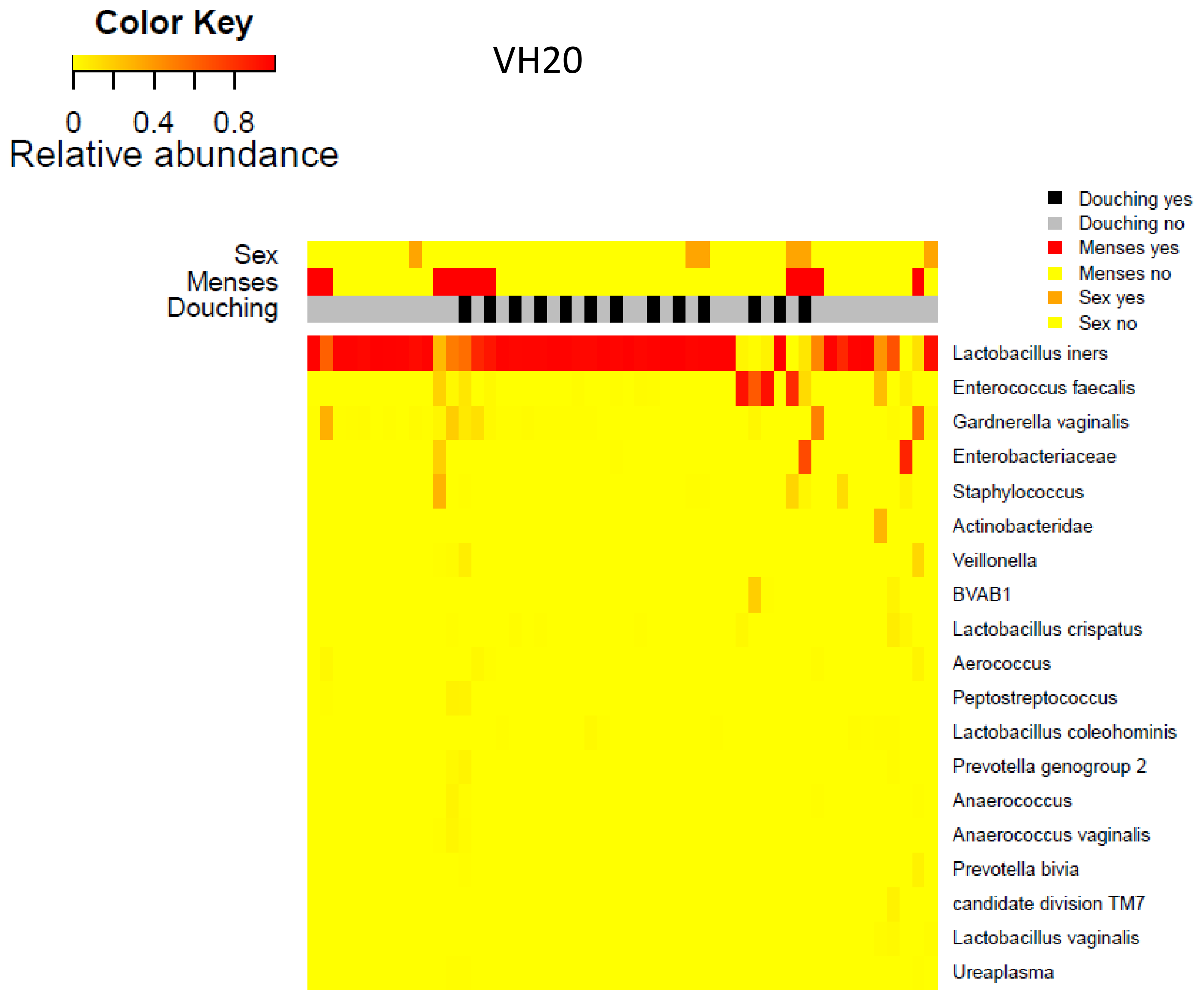

Color Key
0
0.4
0.8
Relative abundance
VH20
Douching yes
Douching no
Menses yes
Menses no
Sex yes
Sex no
Sex
Menses
Douching
Lactobacillus iners
Enterococcus faecalis
Gardnerella vaginalis
Enterobacteriaceae
Staphylococcus
Actinobacteridae
Veillonella
BVAB1
Lactobacillus crispatus
Aerococcus
Peptostreptococcus
Lactobacillus coleohominis
Prevotella genogroup 2
Anaerococcus
Anaerococcus vaginalis
Prevotella bivia
candidate division TM7
Lactobacillus vaginalis
Ureaplasma

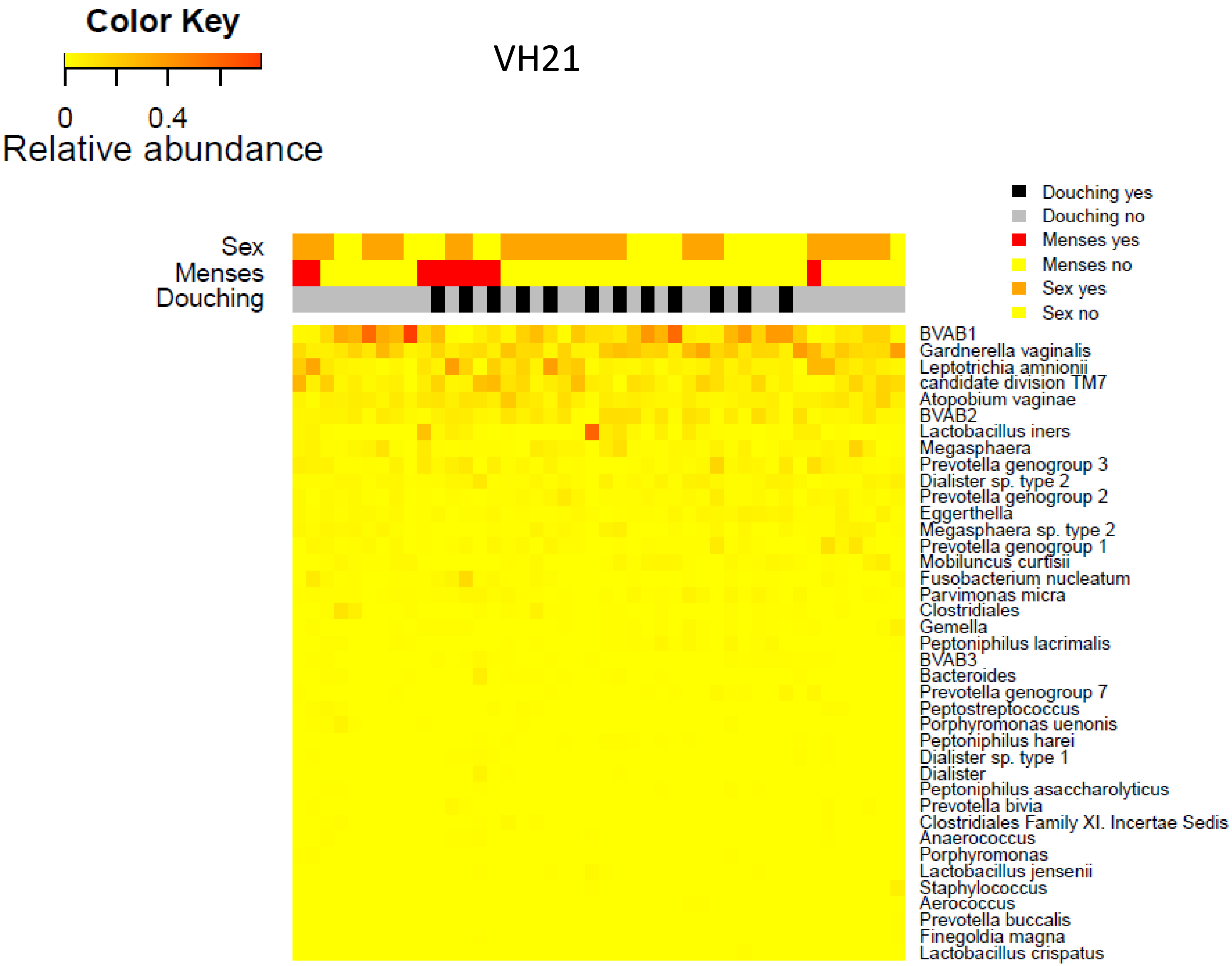

Color Key
0
0.4
Relative abundance
VH21
Douching yes
Douching no
Menses yes
Menses no
Sex yes
Sex no
Sex
Menses
Douching
BVAB1
Gardnerella vaginalis
Leptotrichia amnionii
candidate division TM7
Atopobium vaginae
BVAB2
Lactobacillus iners
Megasphaera
Prevotella genogroup 3
Dialister sp. type 2
Prevotella genogroup 2
Eggerthella
Megasphaera sp. type 2
Prevotella genogroup 1
Mobiluncus curtisii
Fusobacterium nucleatum
Parvimonas micra
Clostridiales
Gemella
Peptoniphilus lacrimalis
BVAB3
Bacteroides
Prevotella genogroup 7
Peptostreptococcus
Porphyromonas uenonis
Peptoniphilus harei
Dialister sp. type 1
Dialister
Peptoniphilus asaccharolyticus
Prevotella bivia
Clostridiales Family XI. Incertae Sedis
Anaerococcus
Porphyromonas
Lactobacillus jensenii
Staphylococcus
Aerococcus
Prevotella buccalis
Finegoldia magna
Lactobacillus crispatus

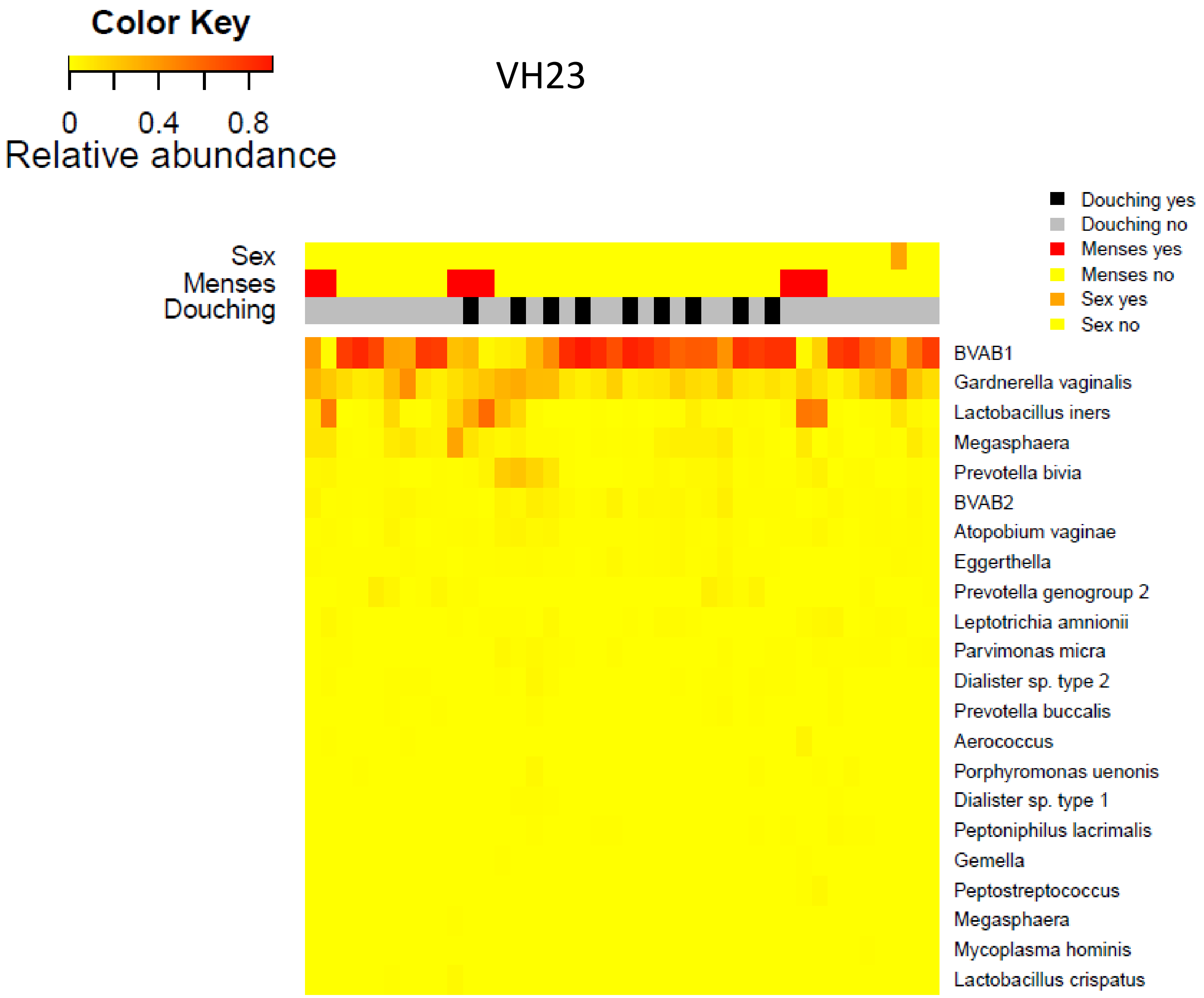
Color Key
0
0.4
0.8
Relative abundance
VH23
Douching yes
Douching no
Menses yes
Menses no
Sex yes
Sex no
Sex
Menses
Douching
BVAB1
Gardnerella vaginalis
Lactobacillus iners
Megasphaera
Prevotella bivia
BVAB2
Atopobium vaginae
Eggerthella
Prevotella genogroup 2
Leptotrichia amnionii
Parvimonas micra
Dialister sp. type 2
Prevotella buccalis
Aerococcus
Porphyromonas uenonis
Dialister sp. type 1
Peptoniphilus lacrimalis
Gemella
Peptostreptococcus
Megasphaera
Mycoplasma hominis
Lactobacillus crispatus

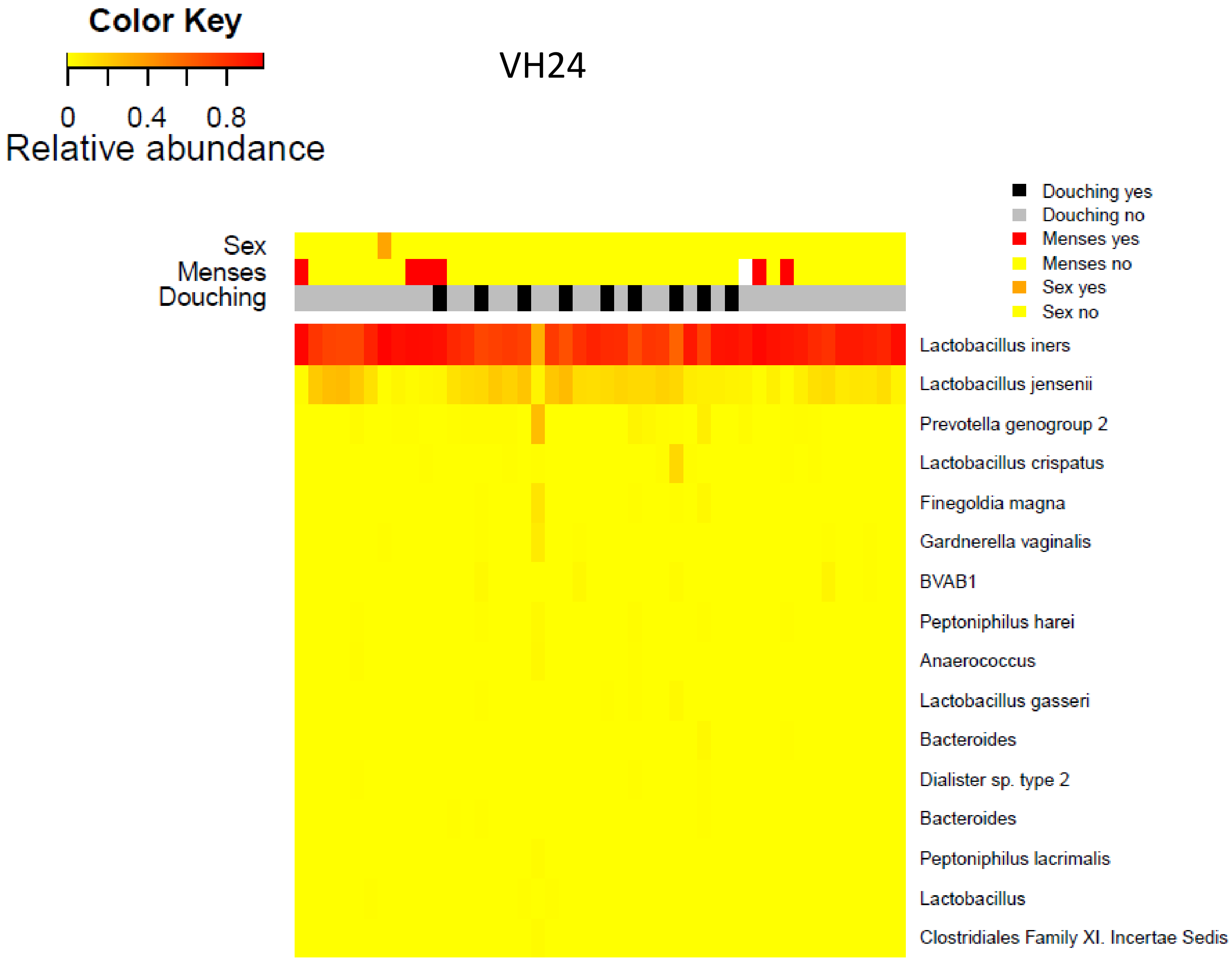

Color Key
0
0.4
0.8
Relative abundance
VH24
Douching yes
Douching no
Menses yes
Menses no
Sex yes
Sex no
Sex
Menses
Douching
Lactobacillus iners
Lactobacillus jensenii
Prevotella genogroup 2
Lactobacillus crispatus
Finegoldia magna
Gardnerella vaginalis
BVAB1
Peptoniphilus harei
Anaerococcus
Lactobacillus gasseri
Bacteroides
Dialister sp. type 2
Bacteroides
Peptoniphilus lacrimalis
Lactobacillus
Clostridiales Family XI. Incertae Sedis

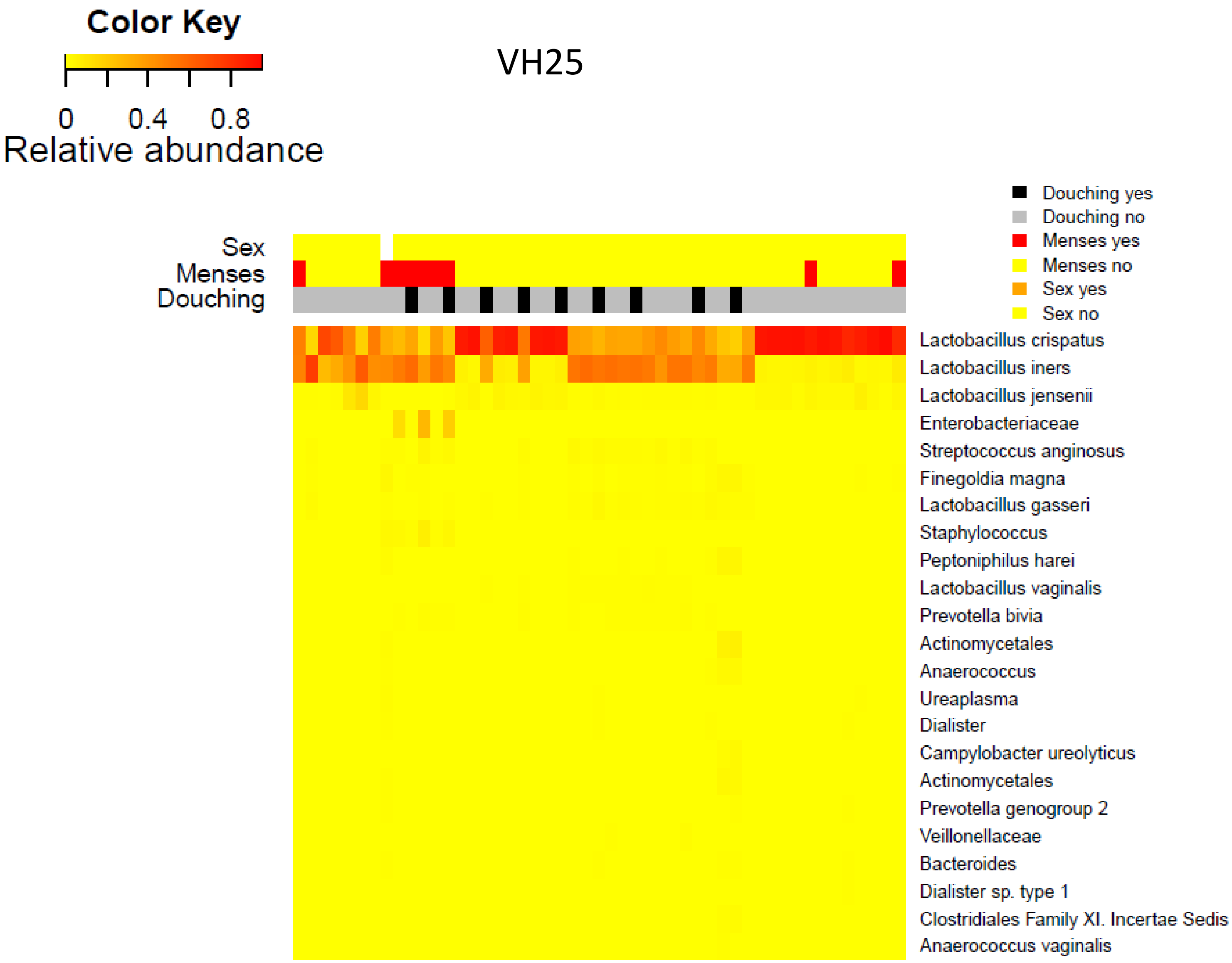

Color Key
0
0.4
0.8
Relative abundance
VH25
Sex
Menses
Douching
Douching yes
Douching no
Menses yes
Menses no
Sex yes
Sex no
Lactobacillus crispatus
Lactobacillus iners
Lactobacillus jensenii
Enterobacteriaceae
Streptococcus anginosus
Finegoldia magna
Lactobacillus gasseri
Staphylococcus
Peptoniphilus harei
Lactobacillus vaginalis
Prevotella bivia
Actinomycetales
Anaerococcus
Ureaplasma
Dialister
Campylobacter ureolyticus
Actinomycetales
Prevotella genogroup 2
Veillonellaceae
Bacteroides
Dialister sp. type 1
Clostridiales Family XI. Incertae Sedis
Anaerococcus vaginalis

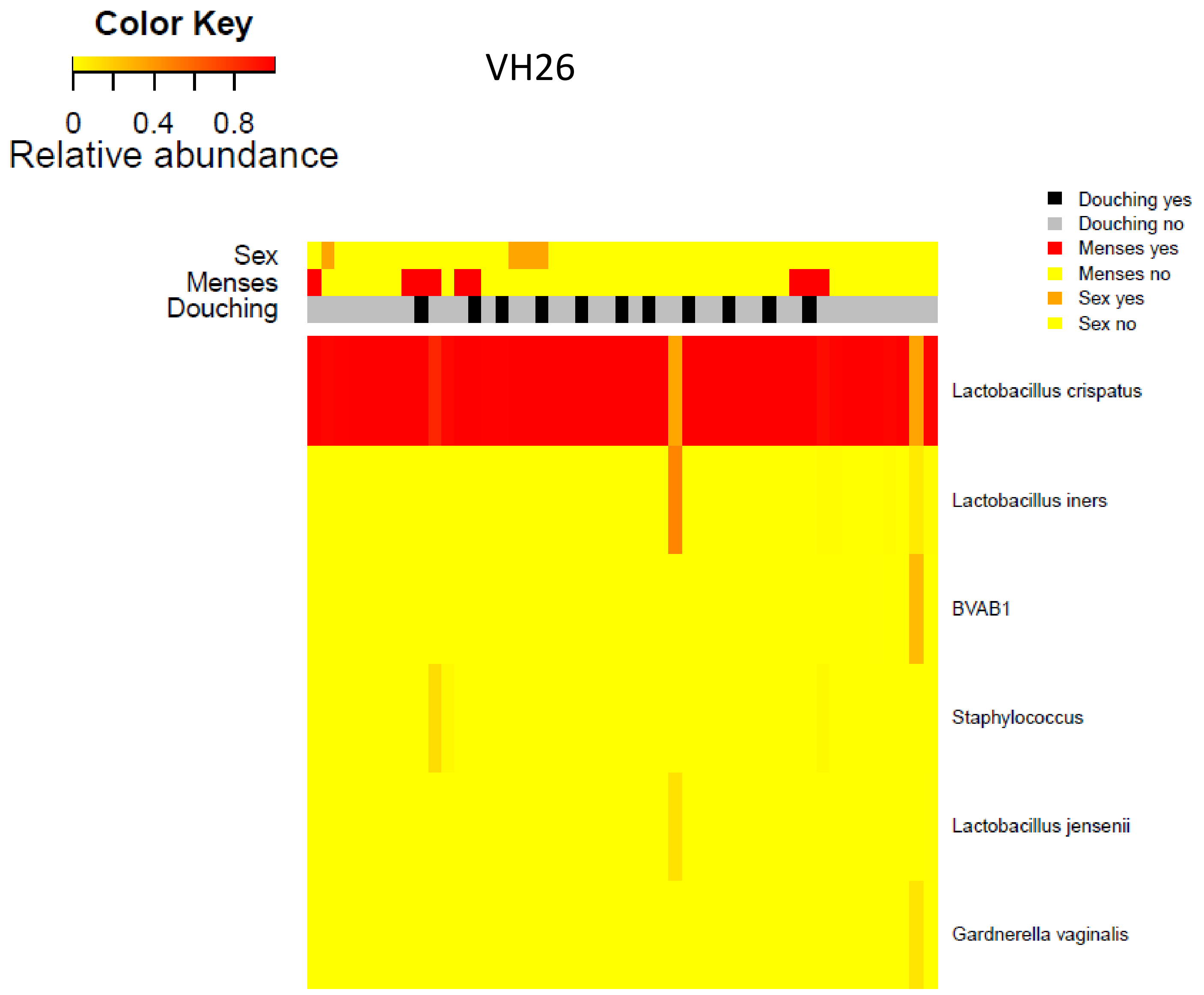
Color Key
0
0.4
0.8
Relative abundance
VH26
Sex
Menses
Douching
Douching yes
Douching no
Menses yes
Menses no
Sex yes
Sex no
Lactobacillus crispatus
Lactobacillus iners
BVAB1
Staphylococcus
Lactobacillus jensenii
Gardnerella vaginalis

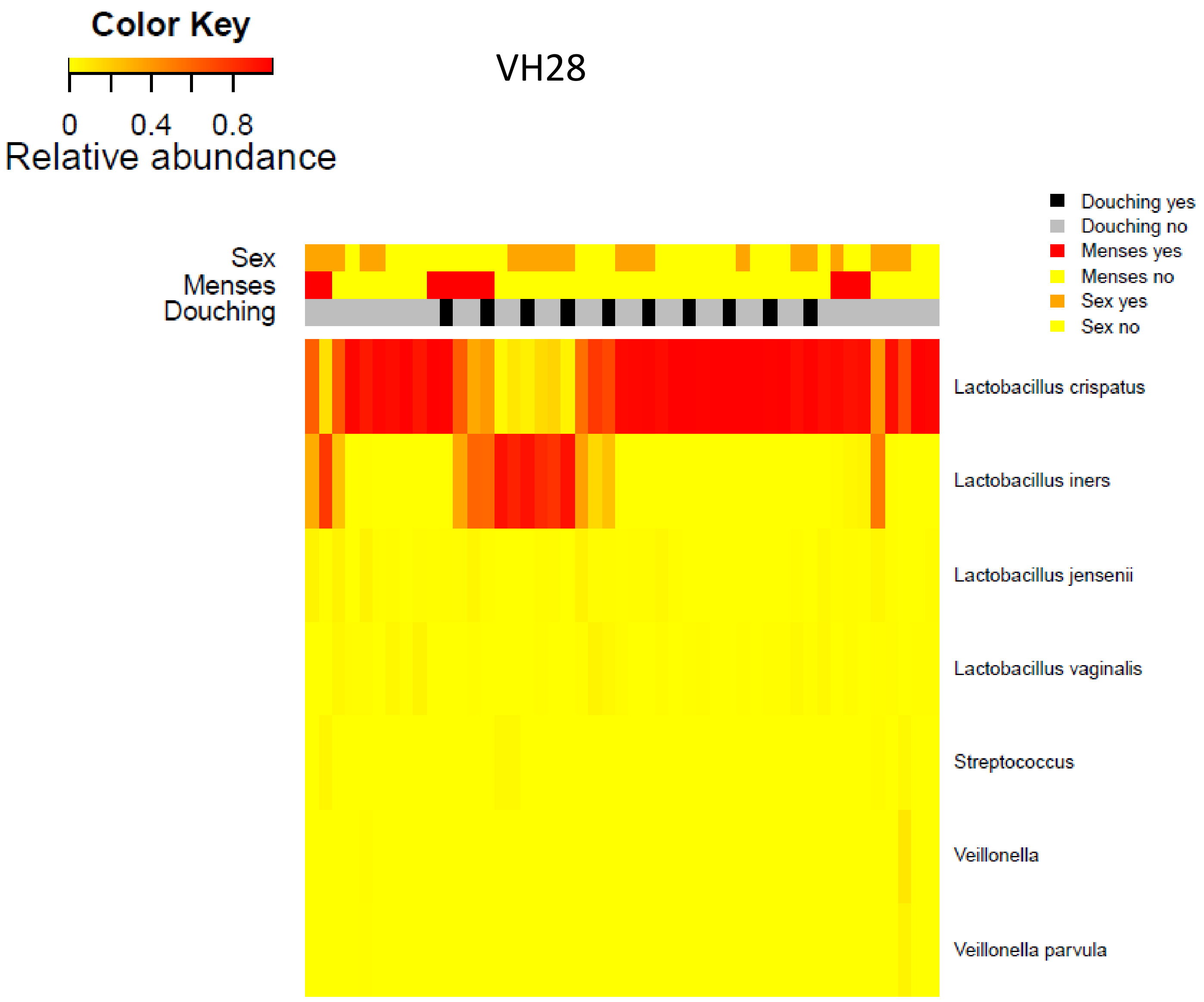
Color Key
0
0.4
0.8
Relative abundance
VH28
Sex
Menses
Douching
Douching yes
Douching no
Menses yes
Menses no
Sex yes
Sex no
Lactobacillus crispatus
Lactobacillus iners
Lactobacillus jensenii
Lactobacillus vaginalis
Streptococcus
Veillonella
Veillonella parvula

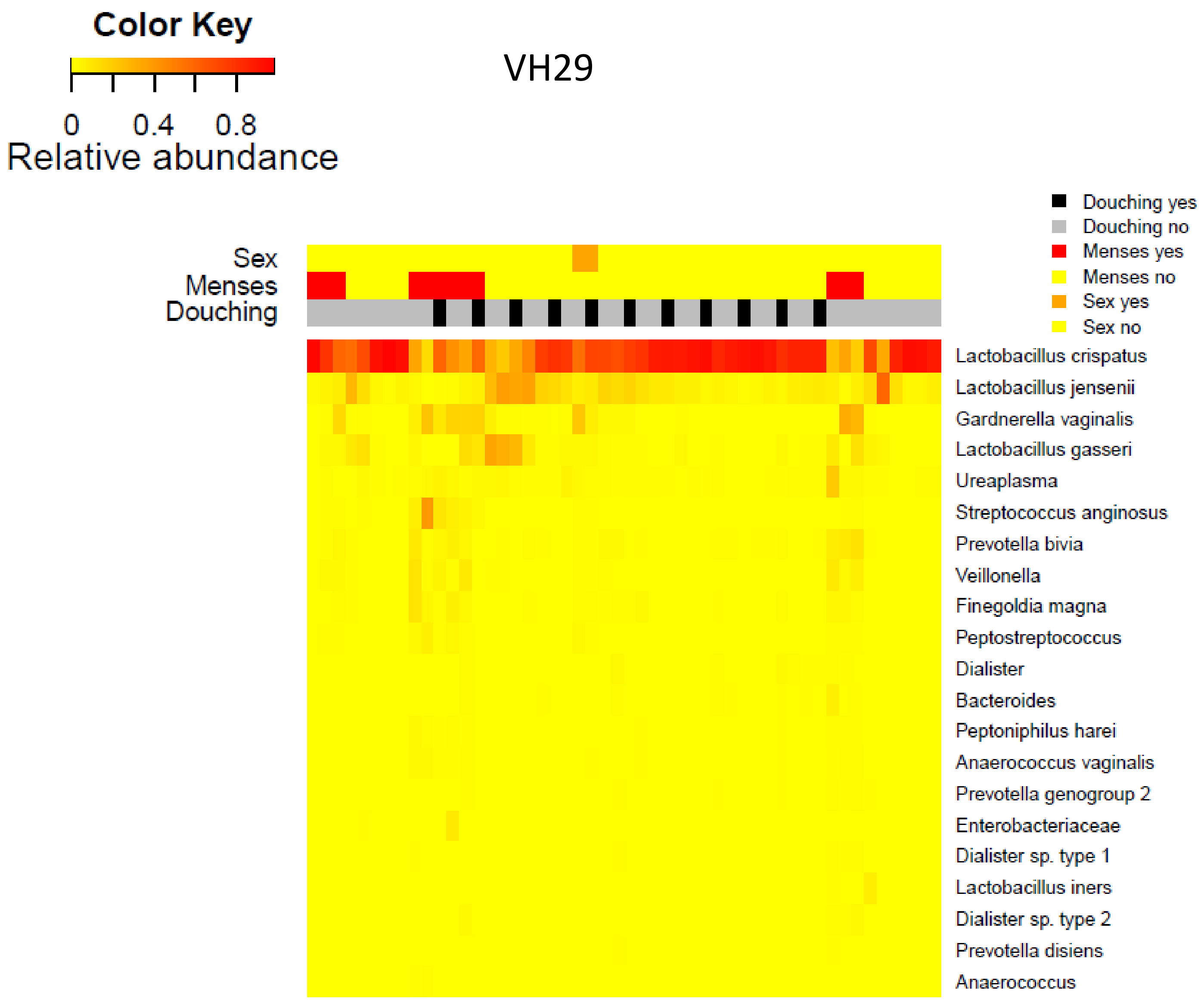
Color Key
0
0.4
0.8
Relative abundance
VH29
Sex
Menses
Douching
Douching yes
Douching no
Menses yes
Menses no
Sex yes
Sex no
Lactobacillus crispatus
Lactobacillus jensenii
Gardnerella vaginalis
Lactobacillus gasseri
Ureaplasma
Streptococcus anginosus
Prevotella bivia
Veillonella
Finegoldia magna
Peptostreptococcus
Dialister
Bacteroides
Peptoniphilus harei
Anaerococcus vaginalis
Prevotella genogroup 2
Enterobacteriaceae
Dialister sp. type 1
Lactobacillus iners
Dialister sp. type 2
Prevotella disiens
Anaerococcus
