# Supplementary figures and images for: Effects of an over-the-counter lactic-acid containing intra-vaginal douching product on the vaginal microbiota
Source: BMC Microbiol. 2019 Jul 25;19:168. doi: 10.1186/s12866-019-1545-0 (PMC6659218; doi:10.1186/s12866-019-1545-0)

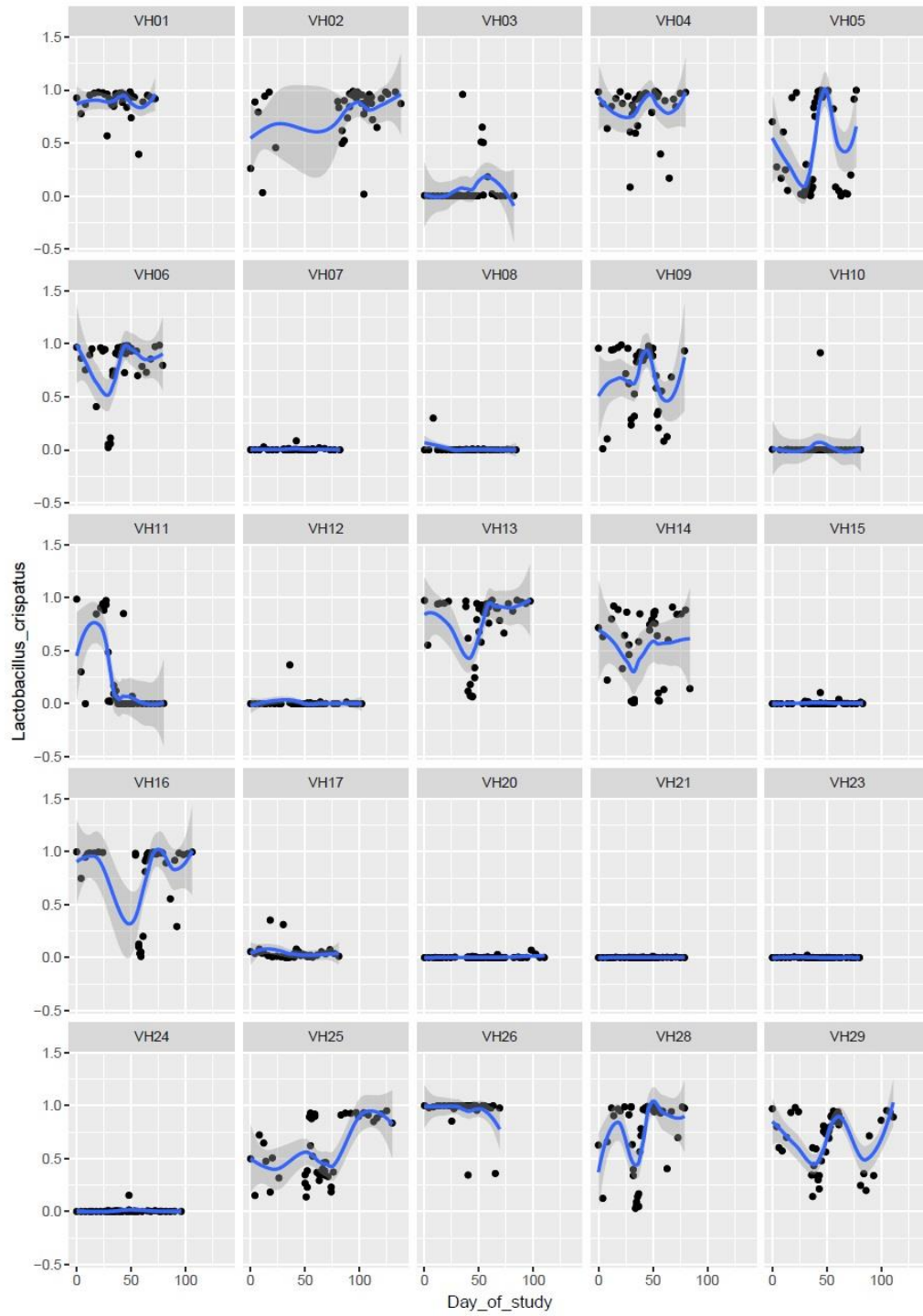

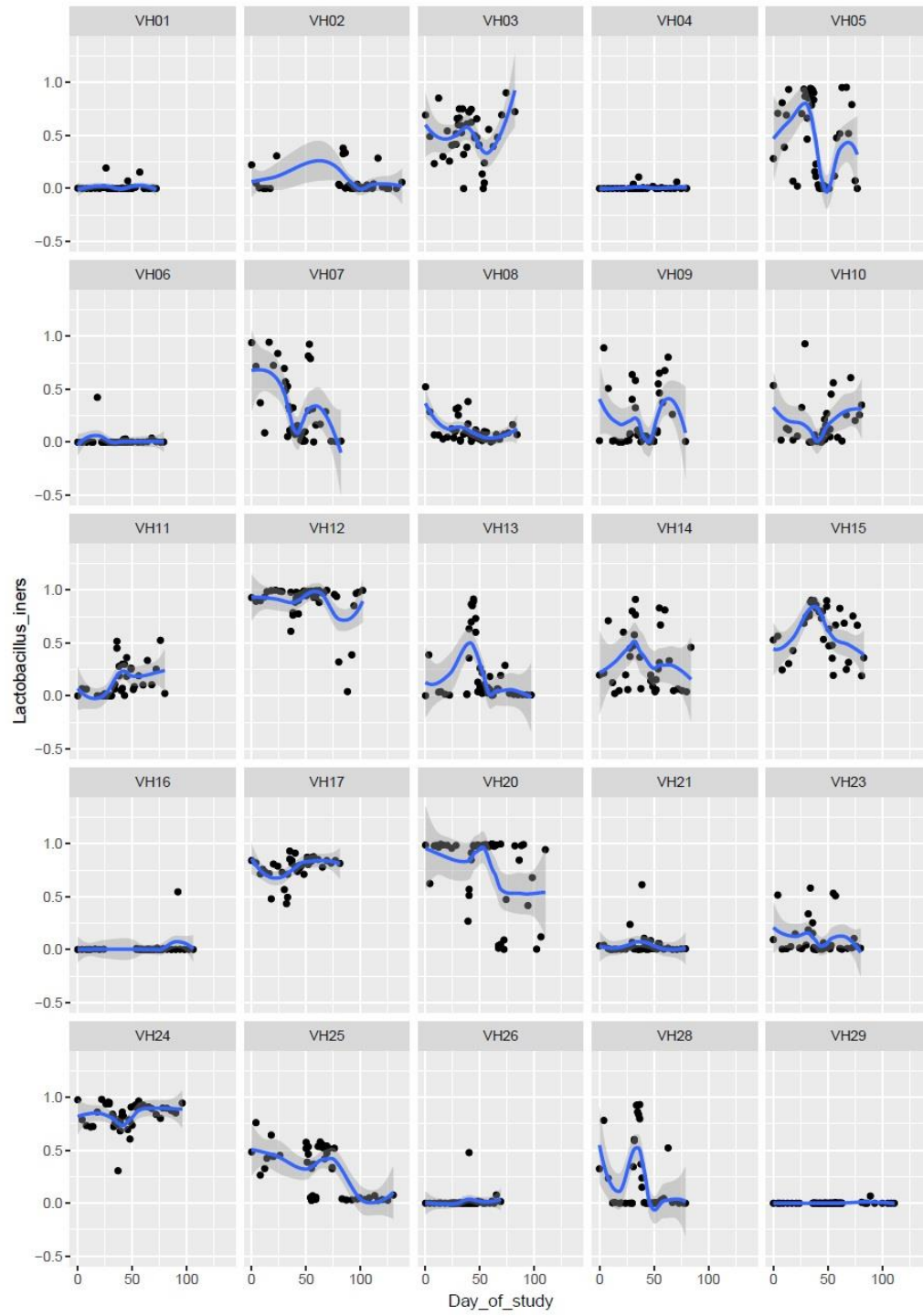

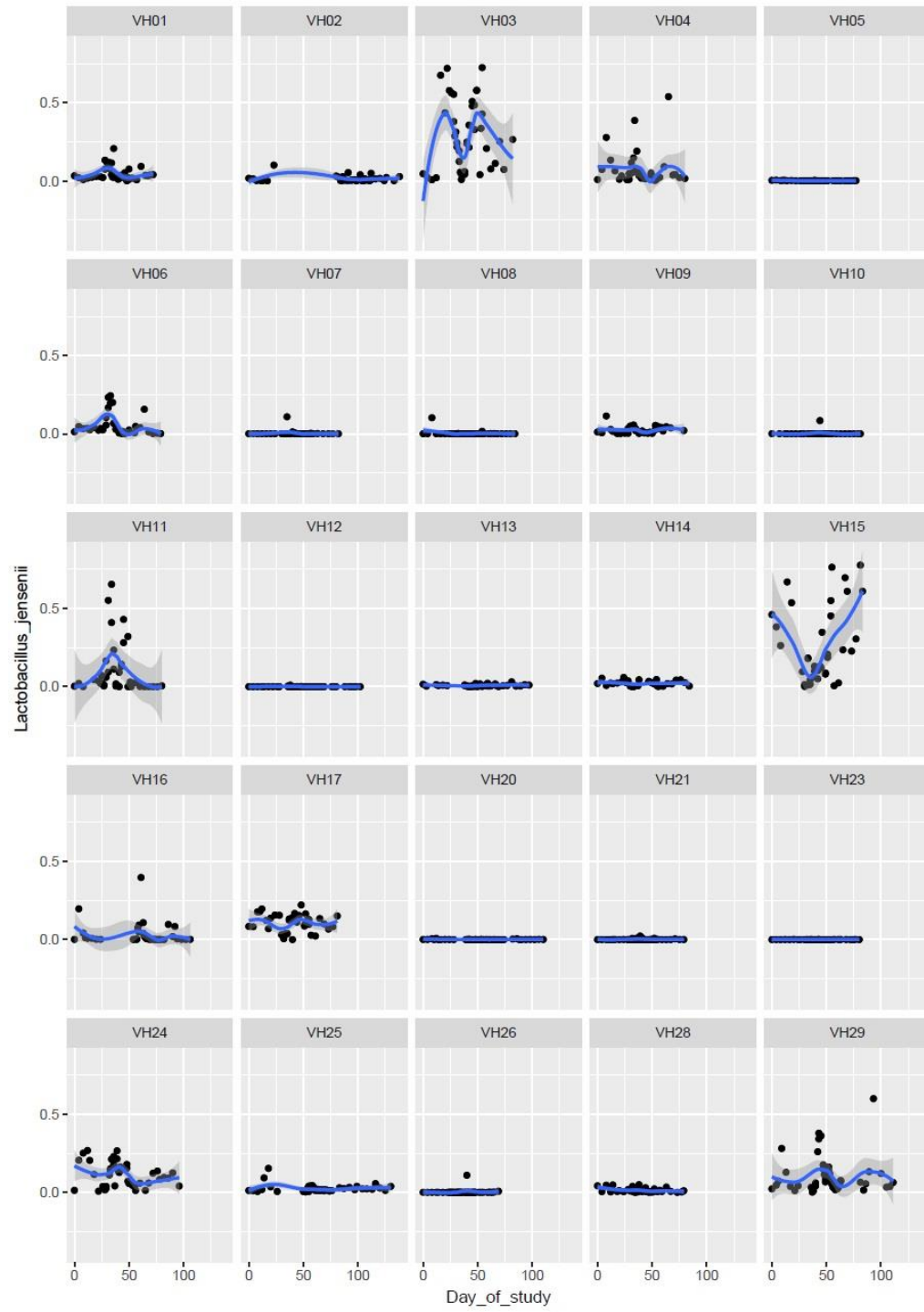

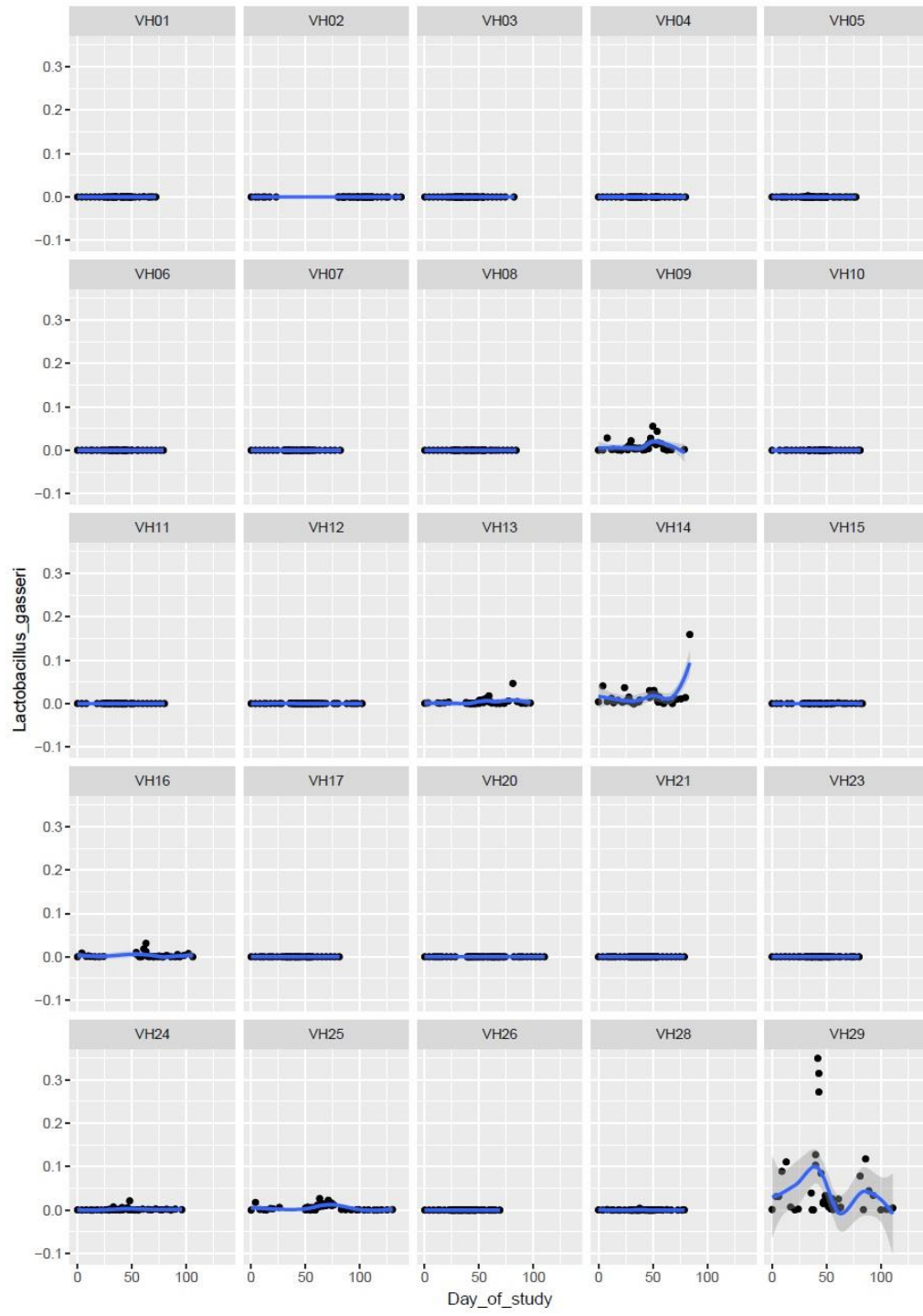

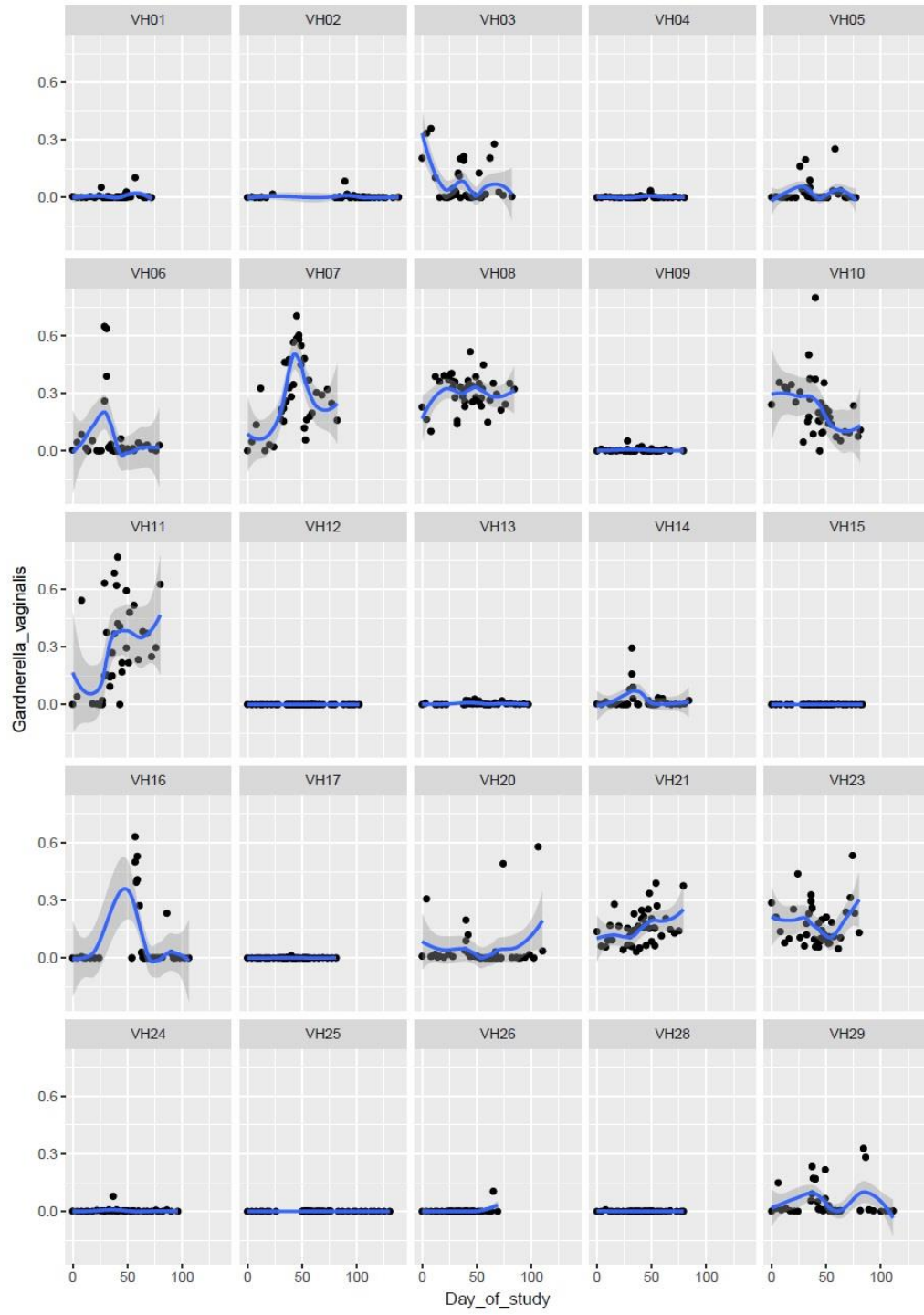

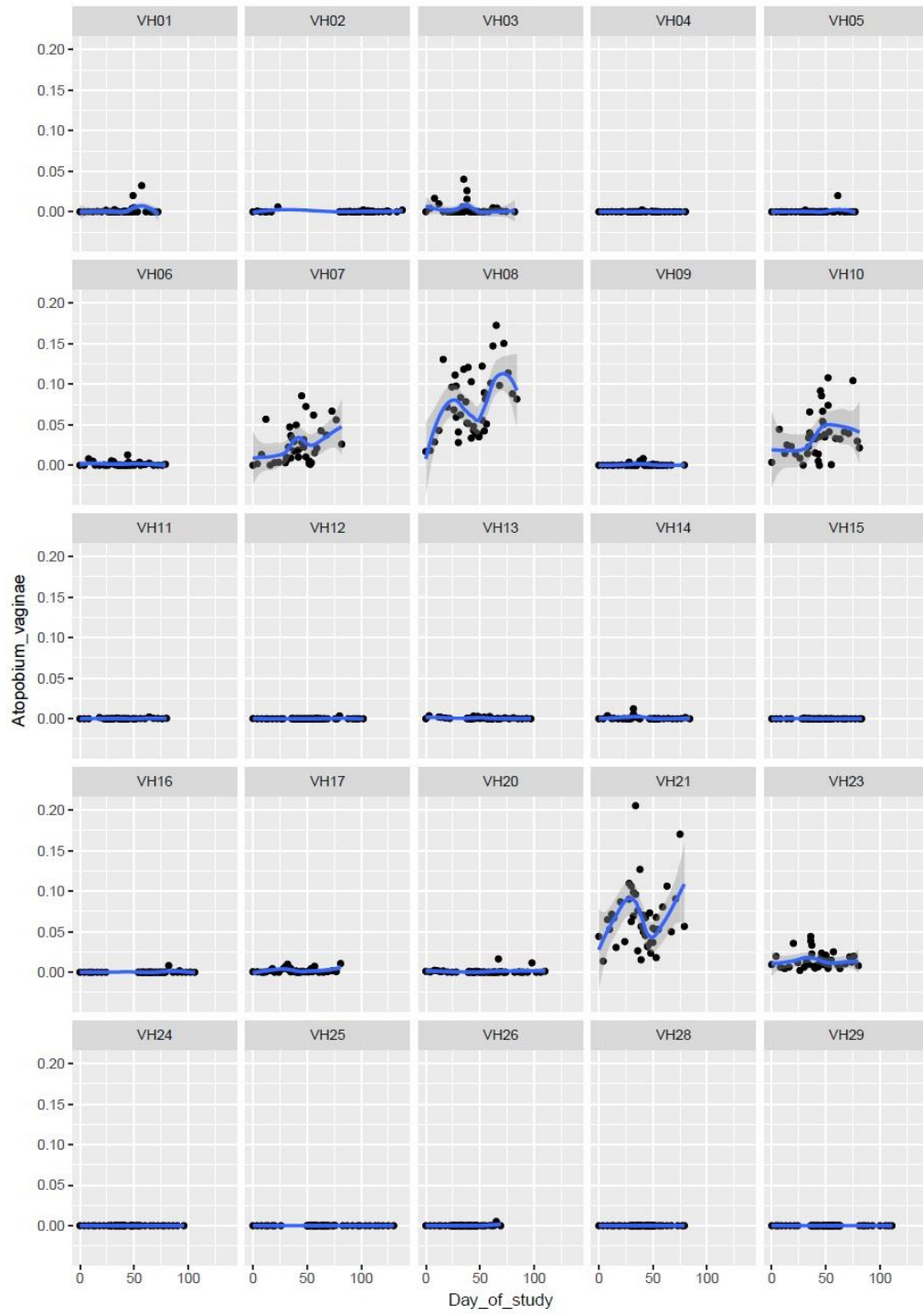

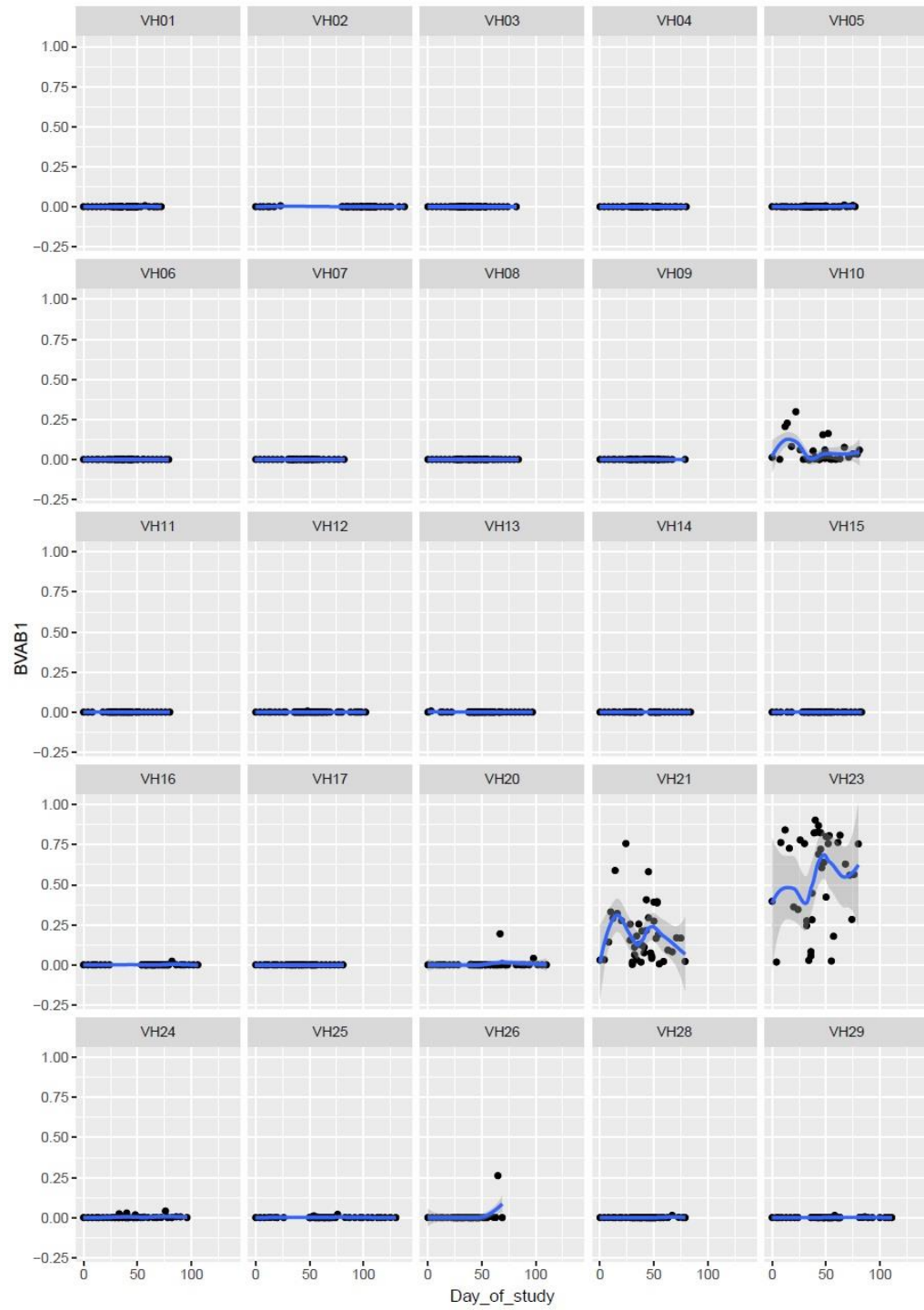

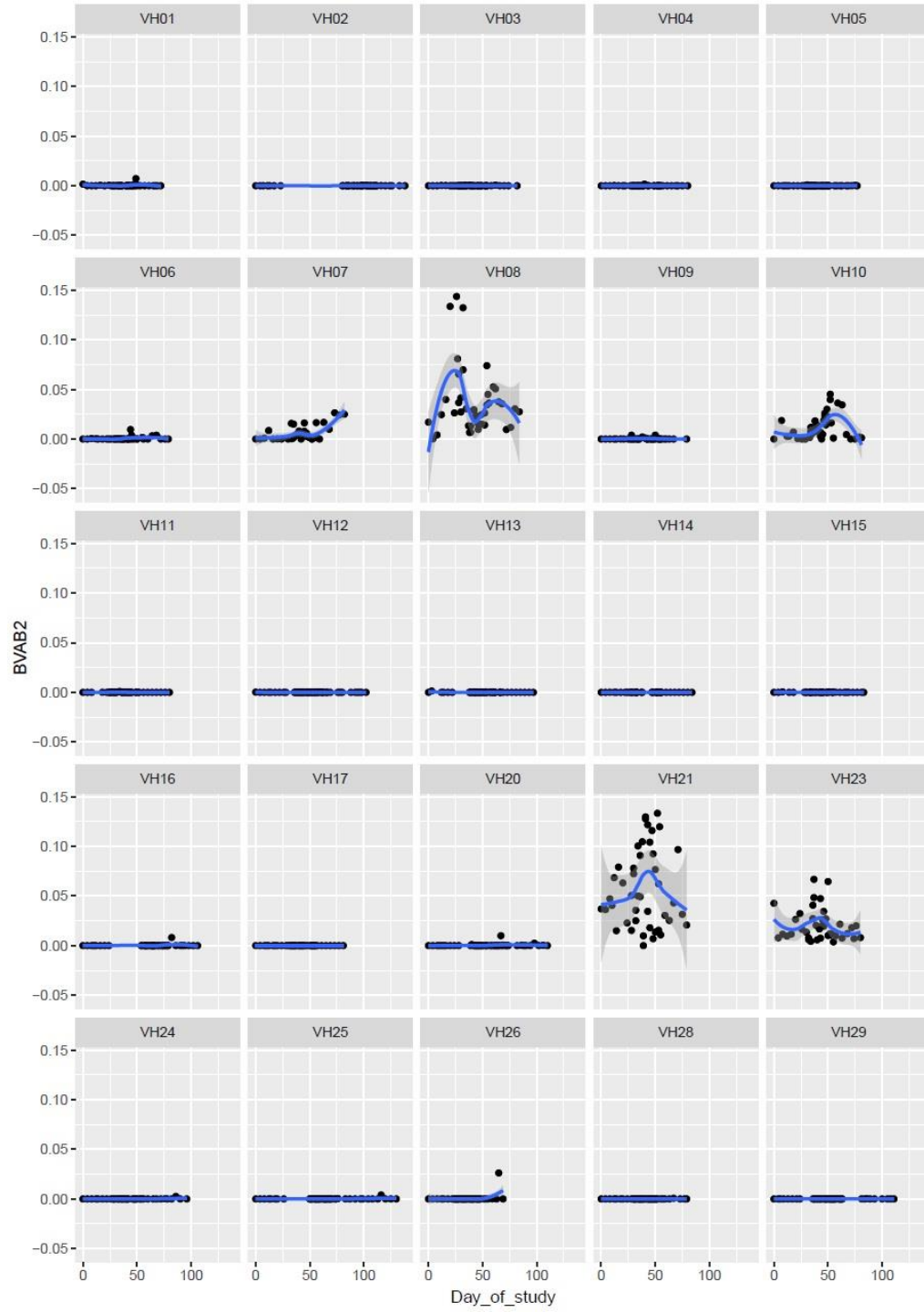

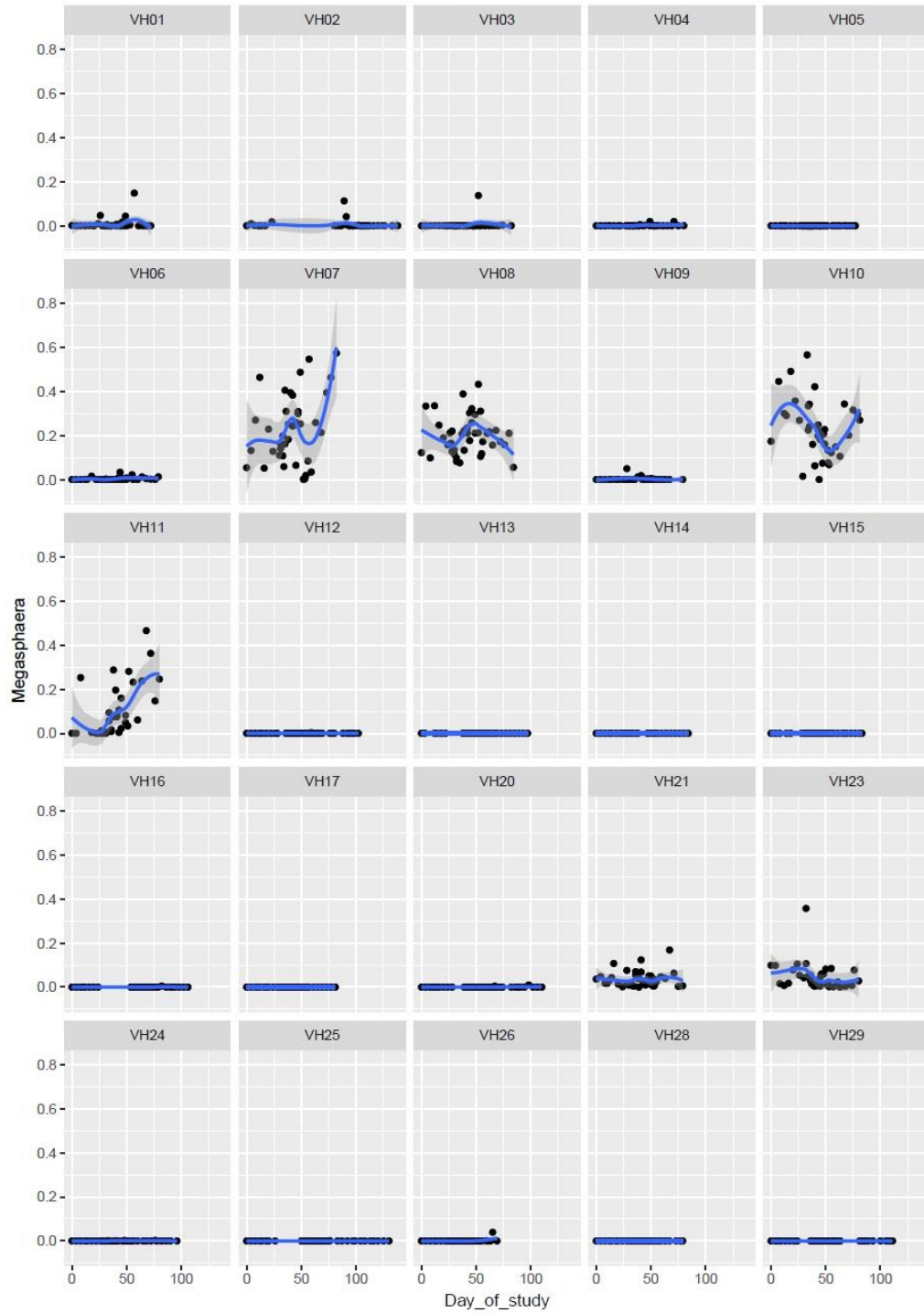

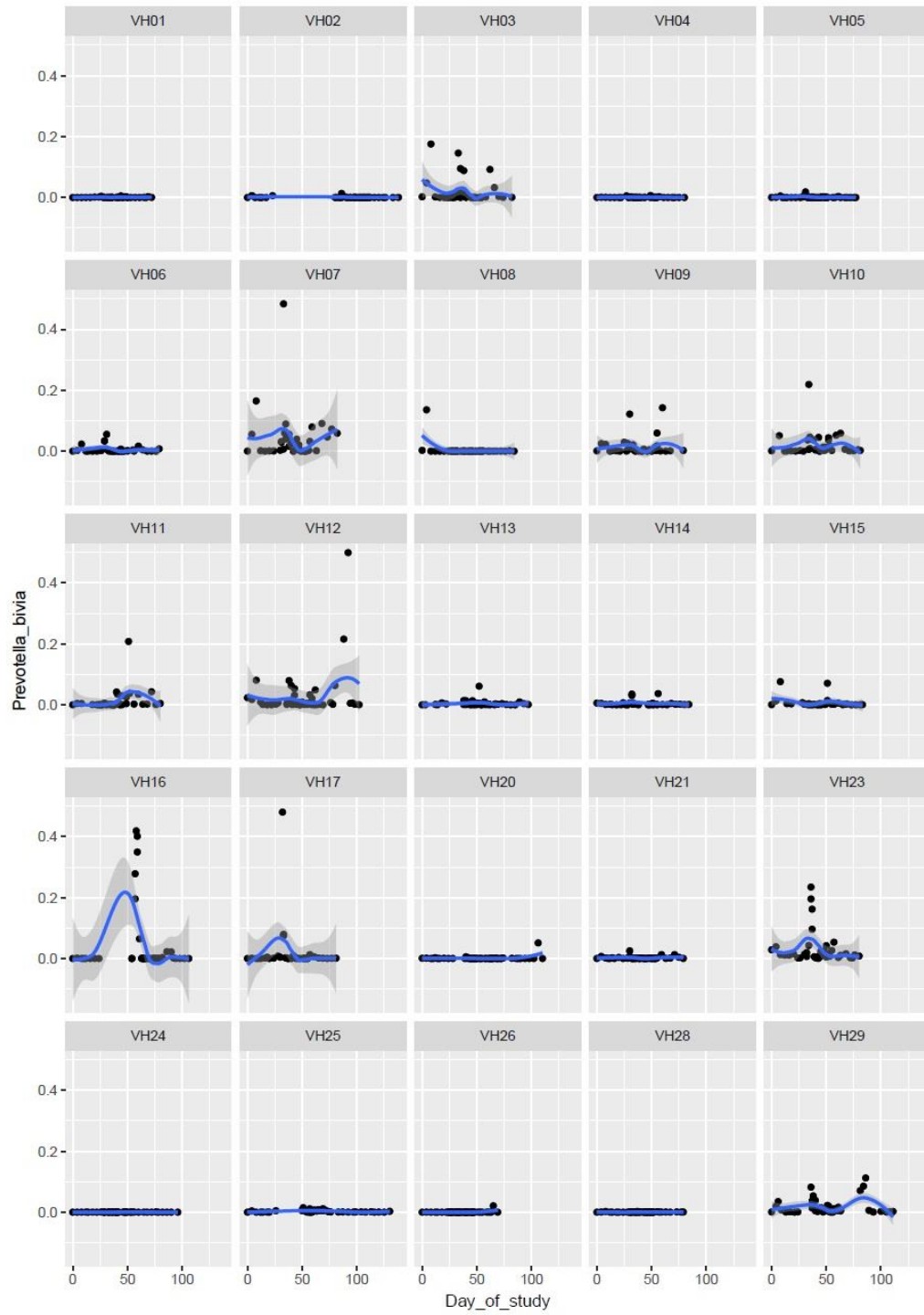

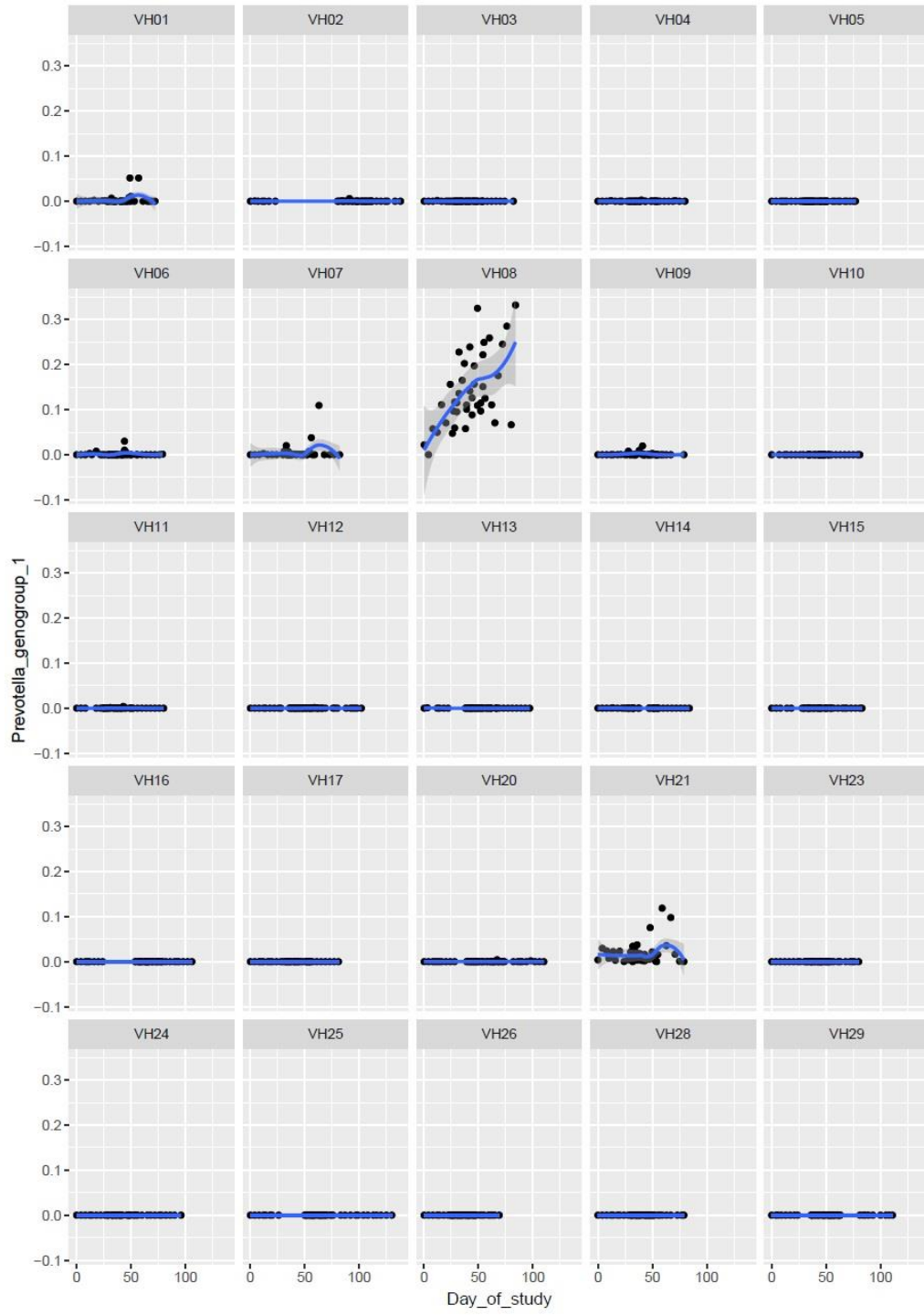

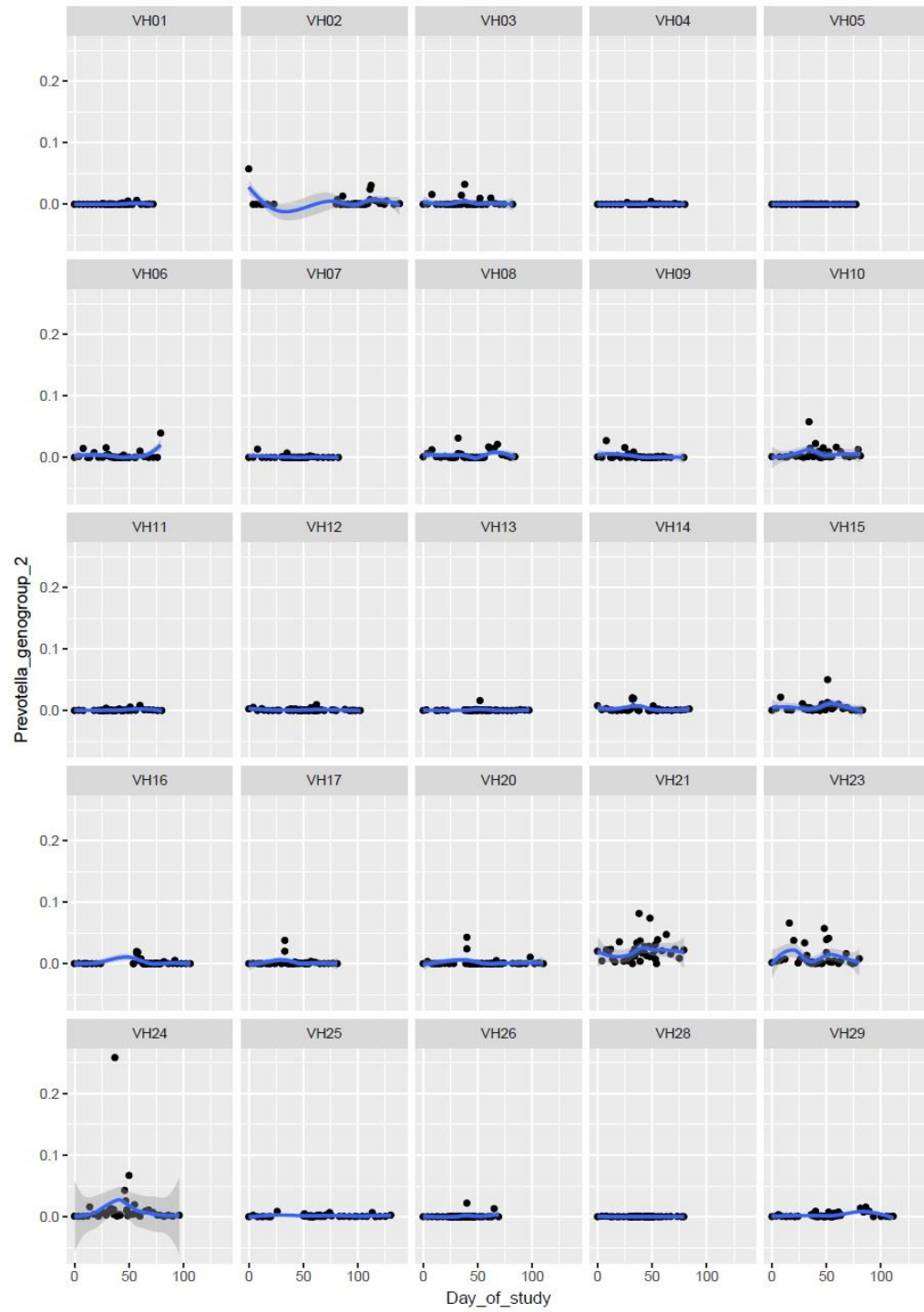

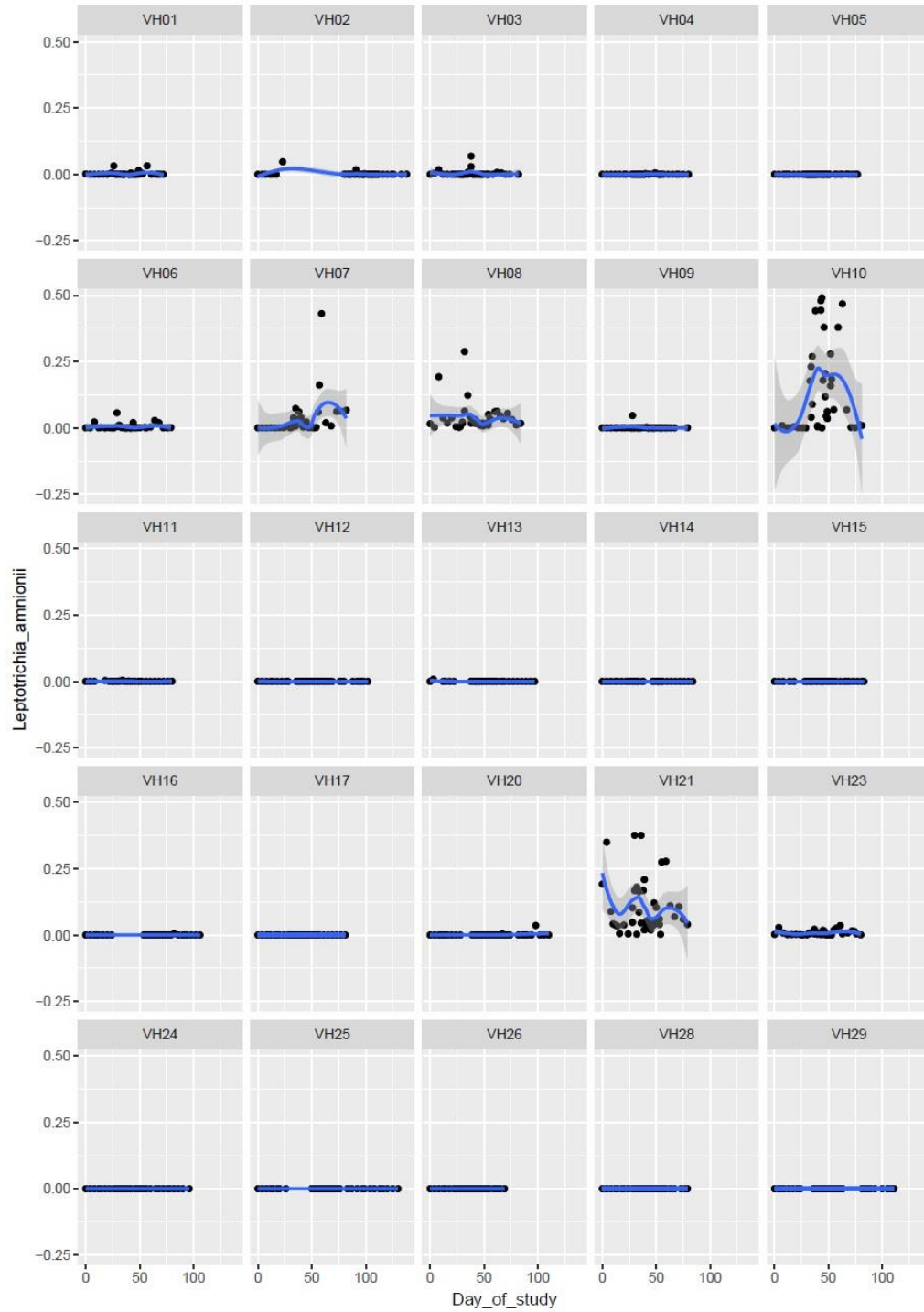

Supplement: Supplementary file 4 — Relative abundances per bacterial species. (PDF 1692 kb) [file 12866_2019_1545_MOESM4_ESM.pdf]
